# Exploring Novel Drug Combinations: The Therapeutic Potential of Selanyl Derivatives for Leishmania Treatment

**DOI:** 10.3390/molecules28155845

**Published:** 2023-08-03

**Authors:** Andreina Henriquez-Figuereo, Esther Moreno, Carmen Sanmartin, Daniel Plano

**Affiliations:** 1 Department of Pharmaceutical Technology and Chemistry, Faculty of Pharmacy and Nutrition, University of Navarra, 31008 Pamplona, Spain; ahenriquez.3@alumni.unav.es (A.H.-F.); emorenoa@unav.es (E.M.); 2 Institute of Tropical Health (ISTUN), University of Navarra, 31008 Pamplona, Spain; 3 Navarra Institute for Health Research (IdisNA), 31008 Pamplona, Spain

**Keywords:** selenium, *Leishmania*, selanyl, drug-combination, synergy

## Abstract

This work describes the design, synthesis, and biological activities of new selenoester derivatives and its homologs thioesters. Thirty-two compounds were developed following an economical synthetic route, achieving small molecules, with structural characteristics similar to those present in antileishmanial drugs such as miltefosine (MIL) and paromomycin (PMN). These compounds were tested in vitro against strains of *Leishmania major* (*L. major*) and *Leishmania infantum* (*L. infantum*). The *L. infantum* strain (causative agent of visceral leishmaniasis) exhibited the highest sensitivity. Thus, four selanylacetic acid derivatives (**A4, A5, A6** and **A8**) presented IC_50_ values below 40 µM in this strain. These derivatives also demonstrated low toxicity and high selectivity in PMA-differentiated THP-1 macrophages. The **A4**–**A6** and **A8** derivatives were evaluated in order to determine their pharmacological behavior, using drug combination studies with the reference drugs amphotericin B (AMB), MIL and PMN. Compounds **A6** and **A8** presented a potent synergistic interaction with MIL, which is the only oral drug available for the treatment of visceral leishmaniasis. Therefore, compounds **A6** and **A8** present significant potential as therapeutic candidates for the treatment of leishmaniasis based on their remarkable leishmanicidal characteristics and pharmacological synergism.

## 1. Introduction

*Leishmania* is a genus of protists comprising more than 17 different species that cause the disease known as leishmaniasis [1]. The disease affects impoverished populations around the World and is mainly associated with malnutrition, population displacement, poor housing conditions, environmental factors, and a general lack of resources [2,3]. The disease has remained as a highly neglected health problem and has become a serious obstacle to the socio-economic development of the affected countries [4]. *Leishmania* parasites have a complex life cycle as dysmorphic protozoan organisms [5]. The parasite has two forms, each one representing an adaptation to the environmental conditions that the parasite confronts in each of its two hosts: the mammal (amastigote) and its insect vector (promastigote) [6]. The clinical manifestations of leishmaniasis depend on the strain and the immune system status of the host, and include cutaneous leishmaniasis (CL), the most common form of leishmaniasis characterized by ulcerative lesions [7]; mucocutaneous leishmaniasis (MCL), which produces lesions preferentially located in the mucous membranes of the upper respiratory tract [8]; and visceral leishmaniasis (VL), which is the most severe form of leishmaniasis in which hepatomegaly, splenomegaly, anemia, leucopenia, and thrombocytopenia occur [9]. Currently, the drugs available for the treatment of leishmaniasis have serious limitations such as high toxicity, high cost, limited efficacy, long treatment duration, and development of drug resistance [10].

Selenium (Se) and sulfur (S) are important elements with similar chemical characteristics [11]. They have equivalent oxidation states, covalent radii, electronegativity, and the ability to form multiple bonds. Both elements play important roles in a variety of biological processes. Sulfur compounds have demonstrated cytotoxic and chemopreventive potential, in particular the S derivatives of garlic, which inhibit tumor development in multiple organs [12]. Selenium is essential as a micronutrient and acts as a cofactor for enzymes involved in antioxidant defense and thyroid hormone metabolism [13]. It also exhibits chemopreventive properties and regulates immune function. Selenium compounds are metabolized in more reduced states compared to sulfur derivatives, e.g., selenols, unlike thiols, are more nucleophilic. Comparative studies consistently show that organoselenic compounds have higher antineoplastic and/or chemopreventive activity compared to sulfur analogs [14,15,16]. Our research group has confirmed these findings, observing a significant decrease in antitumor activity when selenium is substituted by sulfur [15,17].

The relationship between Se and parasites, especially in the context of leishmaniasis, has become a captivating area of scientific research, which has been explored over the years by our research group [18,19,20]. The remarkable leishmanicidal activity of Se resides in its influence on several critical factors for the survival and replication of parasites [21]. Through its interaction with the host immune system, Se increases the production of reactive oxygen species and promotes the activation of immune cells, resulting in an enhanced anti-parasitic response [21]. This dual action of Se, targeting both the parasite and the host immune system, positions it as a potent therapeutic agent against leishmaniasis [22].

**Figure 1:**
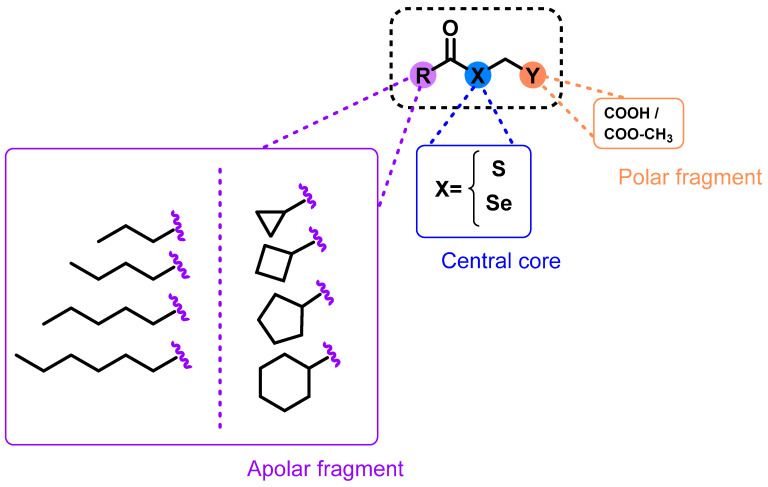
Design and general structure for the proposed compounds.

Bearing this in mind, we report the synthesis and evaluation of 32 novel derivatives containing either Se or S functional groups (Figure 1). For this purpose, different carboxylic acid (linear and cyclic) was used in order to study how structural modifications such as the presence of different bioactive cores, affects the biological activity of these compounds. This strategy can be considered a suitable approach to obtained new compounds, delay the development of resistance, and shorten treatment. The obtained derivatives were evaluated on promastigotes of *Leishmania major* (*L. major*) and *Leishmania infantum* (*L. infantum*). The most active compounds against *Leishmania* parasites were tested against PMA-stimulated THP-1-derived macrophages to determine their toxicity and selectivity. Drug combination studies were also carried out to determine their interaction with the reference drugs amphotericin B (AmB), miltefosine (MIL), and paramomincin (PMN).

## 2. Results and Discussion

### 2.1. Drug Design

Based on our group’s previous experience in the development of selenocompounds, we employed the chemical strategy known as fragment-based design [23] in order to develop 32 novel molecules. The central core of these molecules consisted of selenium (Se) or sulfur (S) atoms linked to a polar head containing carboxylic acid or methyl-carboxylate groups. In addition, an apolar moiety formed by carbo- or heterocyclic structures was incorporated. The introduction of polar and apolar endings in the design (Figure 1) was proposed given that most of the leishmanicidal drugs, such as miltefosine and edelfosine, present this structural feature. This innovative design strategy allowed us to investigate the biological effects resulting from the introduction of Se or S atoms, the incorporation of cyclic or linear chains, and the presence of acid or ester functional groups. In addition, the main objective of this design was to streamline synthetic processes and facilitate the development of new compounds. To the best of our knowledge, the design and biological evaluation of this type of structural modifications had not been studied.

### 2.2. Chemistry

**Figure 2:**
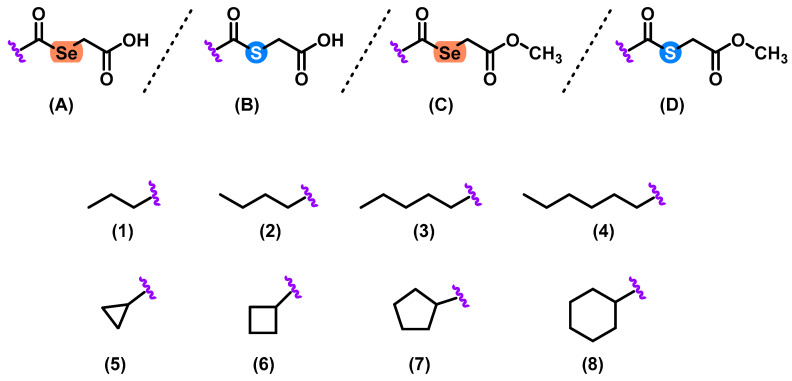
General structures of the 32 Se and S derivatives. Compounds were arranged in 4 series (**A**–**D**) and eight different substituents (**1**–**8**) were used.

Herein, we described the synthesis of 32 selenoester and thioether derivatives (Figure 1). These compounds were obtained by a simple, modular, and efficient one-step synthetic procedure. Se and S atoms were used as central cores and decorated with different linear and cyclic aliphatic substituents. The obtained compounds were grouped in four series (**A1–8**, **B1–8**, **C1–8**, and **D1–8**) (Figure 2) which contained different substituents common to all the series.

**Figure 3:**
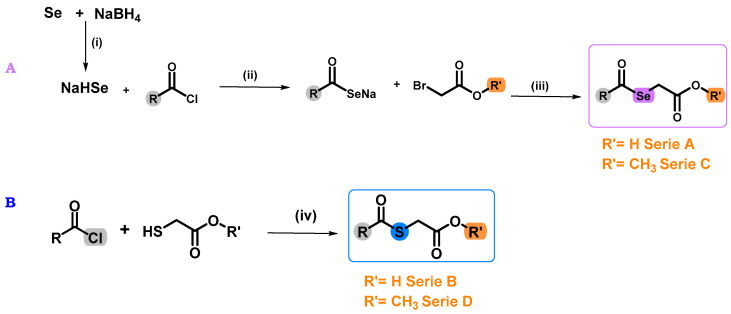
Synthesis of the target Se (compounds **A1**–**A8** and **C1**–**C8**) and S (compounds **B1**–**B8** and **D1**–**D8**) derivatives. Reagents and conditions: (**A**): (i) EtOH, N_2_, 15 min, 0 °C. (ii) THF, room temperature (r.t.). (iii) r.t. for 2 h. (**B**): (iv) DCM, TEA, r.t. for 2 h.

In order to obtain the target compounds, previously published protocol was followed (Figure 3) [24]. For the synthesis of the Se derivatives (series **A** and **C**), EtOH was used as solvent, due to the low water solubility of the acid chlorides used. The exothermic nature of the reaction required the use of an ice bath and a nitrogen (N_2_) atmosphere in order to avoid the degradation of the sodium hydrogen selenite (NaHSe) formed. This intermediate was not isolated, so it was directly reacted with the different chlorides and bromides (Figure 3). In the case of the S derivatives (series **B** and **D**), the synthesis was carried out without form the corresponding S-counterpart, using dichloromethane (DCM) and tetrahydrofuran (THF) as solvents due to the low solubility of the compounds and triethylamine (TEA). This reaction allowed the isolation of the final compounds by evaporation under reduced pressure of the solvent, with no further purification methods. In the case of **A1**–**A8** and **C1**–**C8** derivatives, purification by chromatographic column was necessary. For this reason, the yields of these derivatives are lower than the one obtained for their corresponding S-counterparts. Both Se and S derivatives were stable at room temperature. The structural properties and purity of all compounds were determined by nuclear magnetic resonance (NMR) and spectra are available in the Supplementary Materials (Figures S1–S80).

### 2.3. Leishmanicidal Activity against Leishmania Promastigotes

**Figure 4:**
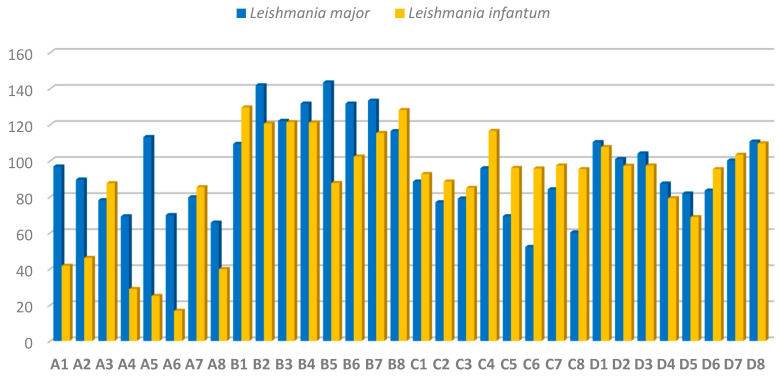
Antileishmanial activity of the 32 derivatives against promastigotes of *L. major* and *L. infantum* expressed as IC_50_ values (mean ± SD) in µM.

The obtained compounds were screened against cultured promastigotes of *L. major* and *L. infantum* based on a previously established protocol [25]. This evaluation was aimed at identifying the lead compounds of this study. Leishmanicidal activity was calculated as the inhibitory concentration 50 (IC_50_), which represents the concentration required to inhibit 50% of promastigotes. Se derivatives (**A1**–**A8** and **C1**–**C8** series) showed higher leishmanicidal activity than their corresponding S counterparts (**B1**–**B8** and **C1**–**C8** series), except for compounds **C4** and **D4**. The detailed results (Figure 4) indicate that the inclusion of Se correlated with a significant increase in the activity of **A1**–**A8** and **C1**–**C8** series, most of which showed an IC_50_ below 100 µM in both strains, except for compounds **A5** (IC_50_ = 113.1 µM) in *L. major* and **C4** (IC_50_ = 116.5 µM) in *L. infantum*. S derivatives (series **B1**–**B8**) showed a different tendency with similar activity (IC_50_ > 100 µM) in both *L. major* and *L. infantum* strains, except for **B5** (IC_50_ = 87.6 µM) against *L. infantum*. Compounds **D1**–**D8** (S derivatives) showed similar leishmanicidal activity against *L. infantum* and *L. major* promastigotes, compounds **D4** (IC_50_ = 87.3 and 79.2 µM), **D5** (IC_50_ = 81.8 and 68.7 µM) and **D6** (IC_50_ = 83.4 and 95.3 µM) being more active towards *L. major* and *L. infantum,* respectively.

*L. infantum* promastigotes were the most sensitive, especially against the selanylacetic acid derivatives **A4**–**A6** and **A8** (IC_50_ below 40 µM) and the thiomethylated derivatives **D2**–**D6** (IC_50_ below 100 µM). These findings suggest that: (i) the nature of the apolar aliphatic fragment (open or cyclic chains) does not significantly influence the potency of the derivatives; (ii) there are no notable differences between the carboxylic acid and methyl ester groups in the polar fragments; (iii) the only clear SAR is that the substitution of the Se atom by S, leads to a decrease in the leishmanicidal effect of the tested derivatives. Compounds **A4**–**A6** and **A8**, were the lead compounds outperforming their S counterparts being 2- to 5-fold more active towards *L. infantum* parasites (Figure 4). These results suggest that the presence of the Se atom, cause an enhancement of the anti-leishmanial activity of the derivatives **A4**–**A6** and **A8**.

### 2.4. In Vitro Toxicity and Selectivity in THP-1 Macrophages

After a first screening against promastigotes of *L. major* and *L. infantum*, those derivatives presenting IC_50_ values below 40 µM were selected to determine their selectivity index (SI) using macrophages (Table 1). SI values represent the ratio of the CC_50_ values calculated in macrophages and the IC_50_ values of parasites. Each compound tested showed superior SI than the reference drugs.

Compounds **A4**–**A6** and **A8** were tested on PMA-stimulated THP-1-derived macrophages to assess their toxicity and selectivity. According to Table 2, these lead compounds (**A4**–**A6** and **A8**) showed low toxicity and high selectivity, outperforming the reference drugs MIL and PMN. These compounds (**A4**–**A6** and **A8**) were 2 or even 3 times more selective than MIL and PMN against *L. major*, and up to 5 times more selective against *L. infantum*. Notably, compound **A5** (cyclopropane selanylacetic acid) was the most prominent, with a CC_50_ = 211.3 µM, while the **A6** derivative (cyclobutane selanylacetic acid) (SI = 11) was the most selective compound evaluated, being 15 and 5 times more selective than MIL and PMN in *L. major*, and 28- and 5-fold more selective than MIL and PMN in *L. infantum*, respectively.

### 2.5. Drug Combination Assay on L. infantum Promastigotes

In view of the current resistance to anti-leishmanial drugs, the current treatment regimen needs to be thoroughly updated and new therapeutic agents are required. Under these circumstances, drug combination is an attractive and effective approach to address this situation. This involves the identification of interactions between two substances, whose combined effects exceed their individual effects. The aim of this approach is to increase treatment efficacy, improve tolerability, reduce treatment duration, reduce treatment costs, and delay the development of resistance. In this regard, we reported the study of pharmacological interaction between aliphatic selanylacetic acid molecules in combination with the standard drugs AMB, MIL, and PMN. Interactions between the lead compounds (**A4**–**A6** and **A8**) and the reference drugs were determined in terms of their combined and individual effect against *L. infantum* promastigotes (Table 2).

To determine the nature of the interaction, it is crucial to choose the appropriate doses. Thus, the evaluation of the combination was carried out by a dose–response linear dilution experiment, for which the IC_50_ value of each substance alone was determined and multiplied by 0.2×, 0.4×, 0.6×, 0.8×, 1×, 1.2×, 1.4×, 1.6×, 1.8×, and 2× times, in order to obtain the different dilutions. Compound **A4** (ƩFICI > 0.60) and **A5** (ƩFICI > 0.60) showed non-interaction combined with the reference drugs AMB, MIL, and PMN. Compound **A6** stood out in this experiment, with synergistic interactions in combination with MIL (ƩFICI = 0.44), borderline synergistic interaction combined with PMN (ƩFICI = 0.54), but without interaction when combined with AMB (ƩFICI = 0.70). Derivative **A8** showed the most outstanding behavior due to the borderline synergistic interaction when combined with AMB (ƩFICI = 0.52) and PMN (ƩFICI = 0.52) and presented a synergistic interaction with MIL (ƩFICI = 0.40) (Table 3). Both **A6** and **A8** were the most outstanding compounds, when combined with fixed ratio solutions of MIL with compounds **A6** and **A8**, exhibited potent synergistic interaction, with a reduction in the IC_50_ of **A6,** decreasing from 16.7 to 5.2 μM, in the case of **A8**, the IC_50_ decreased from 39.9 to 12.39 µM. A similar effect presented MIL combined with **A6** and **A8**, which showed a decrease in the IC_50_ from 31.9 to 6.55–9.29 μM against *L. infantum* promastigotes, respectively.

### 2.6. Theorical ADME and Lipinski Properties

The compounds were screened using the pkCSM software (https://biosig.lab.uq.edu.au/pkcsm/, accessed on 18 July 2023). In addition, Lipinski’s rule of five values were analyzed. Compounds **A4**–**A6** and **A8**, such as MIL, do not violate any of Lipinski’s 5 rules, which is not the case for PMN (3 Lipinski violations). These compounds, during their theorical analysis, showed low skin sensitization and low skin absorbability.

Their predicted high aqueous solubility may be related to the presence of the terminal acid group. Table 3 showed how increase in the number of carbons causes a decrease in solubility, with compound **A5** (−0.92) showing the highest solubility, which decreased in the other compounds, without reaching the solubility of the drugs MIL (−6.15) and PMN (−2.38). Intestinal absorption was high for all compounds, which is considered a valid indicator of drug absorption, outperforming the reference compounds. Overall, selanylacetic acid derivatives appear to share comparable or even superior bioavailability, metabolic stability, and transport properties than the drugs available for the treatment of leishmaniasis.

## 3. Materials and Methods

### 3.1. Chemistry

All reagents were commercially available. The reactions were monitored by thin-layer chromatography (TLC) and the spots were visualized under UV light. The ^1^H,^13^C and ^77^Se-NMR spectra were recorded on a Bruker Avance Neo 400 MHz (Billerica, MA, USA) operating at 400, 100 and 76 MHz, respectively, using deuterated solvent CDCl_3_. Chemical shifts (δ) are reported in parts per million (ppm) and the coupling constants (*J*) are expressed in Hertz. Elemental analyses for carbon, hydrogen and nitrogen were performed on a Thermo Fisher FlashSmart™ Elemental Analyzer (Waltham, MA, USA). Melting points (mp) were determined with a Mettler FP82 + FP80 apparatus (Greifensee, Switzerland). The synthesis of the derivatives of series **A1**–**A8** and **C1**–**C8** has sodium hydrogen selenide as a common intermediate, which was synthesized following the previously mentioned protocol.

### 3.2. General Procedure for the Preparation of Selanylacetic Acid Derivatives (**A1**–**A8**)

In a flask under ice and nitrogen atmosphere, 125 mL 0.012 mmol (1 g) elemental selenium was dissolved in 30 mL absolute ethanol. Then, 2 equivalents (eq.) of sodium borohydride (NaBH_4_) were slowly added. The mixture became exothermic and was stirred continuously in order to obtain a homogeneous, bubble-free mixture (15 min). Then, bromoacetic acid was added (moles) followed by the corresponding acid chloride. THF (5 mL) was added due to the low solubility of these compounds, which caused a change in the color of the reaction from yellow to greyish. This reaction was stirred overnight at room temperature. After this time, reaction was stopped, and a greyish precipitated solid was removed by vacuum filtration and the residue was washed with H_2_O (3 × 30 mL) and extracted with DCM (3 × 30 mL). A silica gel chromatographic column with hexane/ethyl acetate in a 1:9 ratio was used as a purification method. Oily compounds were obtained, whose purity and structural characteristics were confirmed by elemental analysis and NMR.

**2-(butyrylselanyl)acetic acid (A1).** From: sodium hydrogen selenide, bromoacetic acid and butyryl chloride. Appearance: Yellow oil. Yield: 15%. ^1^H NMR (400 MHz, CDCl_3_) δ 0.98 (t, *J* = 7.4 Hz, 3H, Alif), 1.73 (h, *J* = 7.4 Hz, 2H, Alif), 2.65 (t, *J* = 7.4 Hz, 2H, Alif), 3.64 (s, 2H, CH_2_–Se) ppm. ^13^C NMR (101 MHz, CDCl_3_) δ = 13.72, 25.23, 49.32, 61.61, 170.34 (–COOH), 199.28 (–COSeR) ppm. ^77^Se NMR (76 MHz, CDCl_3_) δ 561.06 ppm. Anal. Calcd for C_6_H_10_O_3_Se (%): C, 34.46; H, 4.82; N, 0.00. Found: C, 34.55; H, 4.72; N, 0.05.

**2-(pentanoylselanyl)acetic acid (A2).** From: sodium hydrogen selenide, bromoacetic acid, and pentanoyl chloride. Appearance: Colorless oil. Yield: 13%. ^1^H NMR (400 MHz, CDCl_3_) δ 0.92 (t, *J* = 7.3 Hz, 3H, Alif), 1.38 (h, *J* = 7.4 Hz, 2H, Alif), 1.67 (p, *J* = 7.5 Hz, 2H, Alif), 2.67 (t, *J* = 7.5 Hz, 2H, Alif), 3.64 (s, 2H, CH_2_–Se) ppm. ^13^C NMR (101 MHz, CDCl_3_) δ = 13.68, 25.26, 27.37, 47.24, 61.63, 170.36 (–COOH), 199.41(–COSeR) ppm. ^77^Se NMR (76 MHz, CDCl_3_) δ 559.86 ppm. Anal. Calcd for C_7_H_12_O_3_Se (%): C, 37.68; H, 5.42; N, 0.00. Found: C, 37.49; H, 5.50; N, 0.03.

**2-(hexanoylselanyl)acetic acid (A3).** From: sodium hydrogen selenide, bromoacetic acid, and hexanoyl chloride. Appearance: Yellow oil. Yield: 12%. ^1^H NMR (400 MHz, CDCl_3_) δ 0.83 (m, 3H, Alif), 1.19 (t, *J* = 7.1 Hz, 1H, Alif), 1.62 (m, 2H, Alif), 2.59 (t, *J* = 7.5 Hz, 2H, Alif), 3.57 (s, 2H, CH_2_–Se) ppm. ^13^C NMR (101 MHz, CDCl_3_) δ = 13.82, 22.27, 25.01, 25.25, 47.48, 61.62, 170.35(–COOH), 199.41 (–COSeR) ppm. ^77^Se NMR (76 MHz, CDCl_3_) δ 559.29 ppm. Anal. Calcd for C_8_H_14_O_3_Se (%): C, 40.51; H, 5.95; N, 0.00. Found: C, 40.72; H, 5.65; N, 0.11.

**2-(heptanoylselanyl)acetic acid (A4).** From: sodium hydrogen selenide, bromoacetic acid, and heptanoyl chloride. Appearance: Green oil. Yield: 18%. ^1^H NMR (400 MHz, CDCl_3_) δ 0.88 (t, *J* = 6.7 Hz, 3H, Alif), 1.28 (m, 6H, Alif), 1.68 (p, *J* = 7.4 Hz, 2H, Alif), 2.66 (t, *J* = 7.5 Hz, 2H, Alif), 3.64 (s, 2H, CH_2_–Se) ppm. ^13^C NMR (101 MHz, CDCl_3_) δ = 22.41, 28.48, 31.38, 47.53, 170.36(–COOH), 199.43 (–COSeR) ppm. ^77^Se NMR (76 MHz, CDCl_3_) δ 559.75 ppm. Anal. Calcd for C_9_H_16_O_3_Se (%): C, 43.03; H, 6.42; N, 0.00. Found: C, 42.899; H, 6.51; N, 0.09.

**2-((cyclopropanecarbonyl)selanyl)acetic acid (A5).** From: sodium hydrogen selenide, bromoacetic acid, and cyclopropanecarbonyl chloride. Appearance: Colorless oil. Yield: 15%. ^1^H NMR (400 MHz, CDCl_3_) δ 1.05 (m, 2H, Alif), 1.27 (d, *J* = 2.8 Hz, 2H, Alif), 2.14 (m, 1H, Alif), 3.66 (s, 2H, CH_2_–Se) ppm. ^13^C NMR (101 MHz, CDCl_3_) δ = 11.68, 25.33, 61.64, 170.31 (–COOH), 198.89 (–COSeR) ppm. ^77^Se NMR (76 MHz, CDCl_3_) δ 555.77 ppm. Anal. Calcd for C_6_H_8_O_3_Se (%): C, 34.80; H, 3.89; N, 0.00. Found: C, 34.91; H, 4.01; N, 0.07.

**2-((cyclobutanecarbonyl)selanyl)acetic acid (A6).** From: sodium hydrogen selenide, bromoacetic acid, and cyclobutanecarbonyl chloride. Appearance: Colorless oil. Yield: 32%. ^1^H NMR (400 MHz, CDCl_3_) δ 1.05 (s, 2H, Alif), 1.25 (m, 4H, Alif), 2.14 (m, 1H, Alif), 3.66 (s, 2H, CH_2_–Se) ppm. ^13^C NMR (101 MHz, CDCl_3_) δ = 11.68, 14.09, 25.33, 25.84, 61.64, 170.31(–COOH), 198.89(–COSeR) ppm. ^77^Se NMR (76 MHz, CDCl_3_) δ 555.77 ppm. Anal. Calcd for C_7_H_10_O_3_Se (%): C, 38.02; H, 4.56; N, 0.00. Found: C, 37.89; H, 4.50; N, 0.08.

**2-((cyclopentanecarbonyl)selanyl)acetic acid (A7).** From: sodium hydrogen selenide, bromoacetic acid, and cyclopentanecarbonyl chloride. Appearance: Colorless oil. Yield: 17%. ^1^H NMR (400 MHz, CDCl_3_) δ 1.61 (m, 2H, Alif), 1.71 (m, 2H, Alif), 1.89 (m, 4H, Alif), 3.06 (q, *J* = 7.8 Hz, 1H, Alif), 3.62 (s, 2H, CH_2_–Se) ppm. ^13^C NMR (101 MHz, CDCl_3_) δ = 25.12, 25.70, 30.14, 56.48, 61.51, 170.39 (–COOH), 202.77 (–COSeR) ppm. ^77^Se NMR (76 MHz, CDCl_3_) δ 542.62 ppm. Anal. Calcd for C_8_H_12_O_3_Se (%): C, 40.86; H, 5.14; N, 0.00. Found: C, 41.01; H, 5.25; N, 0.10.

**2-((cyclohexanecarbonyl)selanyl)acetic acid (A8).** From: sodium hydrogen selenide, bromoacetic acid, and cyclohexanecarbonyl chloride. Appearance: Yellow oil. Yield: 22%. ^1^H NMR (400 MHz, CDCl_3_) δ 1.26 (m, 4H, Alif), 1.48 (m, 2H, Alif), 1.80 (m, 2H, Alif), 1.98 (m, 2H, Alif), 2.56 (tt, *J* = 3.6, 11.3 Hz, 2H, Alif), 3.61 (s, 2H, CH_2_–Se) ppm. ^13^C NMR (101 MHz, CDCl_3_) δ = 24.79, 25.31, 25.59, 29.21, 55.69, 61.56, 170.49 (–COOH), 203.27 (–COSeR) ppm. ^77^Se NMR (76 MHz, CDCl_3_) δ 542.44 ppm. Anal. Calcd for C_9_H_14_O_3_Se (%): C, 43.38; H, 5.66; N, 0.00. Found: C, 43.49; H, 5.52; N, 0.06.

### 3.3. General Procedure for the Preparation of Thioglycolic Acid Derivatives (**B1**–**B8**)

For the synthesis of the **B1**–**B8** series derivatives, 0.010 mmol thioglycolic acid (1 g) was dissolved in DCM (30 mL) and reacted with the corresponding acid chlorides and 1.01 mmol triethylamine [17] for 2 h. After this time, the reaction was stopped and decanted with H_2_O (3 × 30 mL) and DCM (3 × 30 mL). The solvent was evaporated under reduced pressure at a rotary evaporator. Oily liquid compounds were obtained, and their purity and structural features were confirmed by elemental analysis and NMR.

**2-(butyrylthio)acetic acid (B1).** From: thioglycolic acid, triethylamine, and butyryl chloride. Appearance: Light pink oil. Yield: 25%. ^1^H NMR (400 MHz, CDCl_3_) δ 0.97 (t, *J* = 7.4 Hz, 3H, Alif), 1.73 (h, *J* = 7.4 Hz, 2H, Alif), 2.61 (t, *J* = 7.4 Hz, 2H, Alif), 3.74 (s, 2H, CH_2_–S) ppm. ^13^C NMR (101 MHz, CDCl_3_) δ = 13.43, 19.02, 30.95, 45.46, 174.66 (–COOH), 197.56 (–COSR) ppm. Anal. Calcd for C_6_H_10_O_3_S (%): C, 44.43; H, 6.21; N, 0.00. Found: C, 44.56; H, 6.74; N, 0.02.

**2-(pentanoylthio)acetic acid (B2).** From: thioglycolic acid, triethylamine, and pentanoyl chloride. Appearance: Yellow oil. Yield: 32%. ^1^H NMR (400 MHz, CDCl_3_) δ 0.92 (t, *J* = 7.6 Hz, 3H, Alif), 1.37 (h, *J* = 7.3 Hz, 2H, Alif), 1.66 (m, 2H, Alif), 2.63 (t, *J* = 7.5 Hz, 2H, Alif), 3.73 (s, 2H, CH_2_–S) ppm. ^13^C NMR (101 MHz, CDCl_3_) δ = 13.67, 22.05, 27.49, 30.96, 43.39, 174.33(–COOH), 197.72 (–COSR) ppm. Anal. Calcd for C_7_H_12_O_3_S (%): C, 47.71; H, 6.86; N, 0.00. Found: C, 47.62; H, 6.64; N, 0.04.

**2-(hexanoylthio)acetic acid (B3).** From: thioglycolic acid, triethylamine, and hexanoyl chloride. Appearance: Yellow oil. Yield: 33%. ^1^H NMR (400 MHz, CDCl_3_) δ 0.88 (t, *J* = 6.7 Hz, 3H, Alif), 1.35 (m, 2H, Alif), 1.68 (m, 2H, Alif), 2.66 (t, *J* = 7.5 Hz, 2H, Alif), 4.17 (q, *J* = 7.1 Hz, 2H, CH_2_–S) ppm. ^13^C NMR (101 MHz, CDCl_3_) δ = 13.98, 22.40, 28.47, 31.37, 47.52, 61.60, 170.33 (–COOH), 199.39 (–COSR) ppm. Anal. Calcd for C_8_H_14_O_3_S (%): C, 50.50; H, 7.42; N, 0.00. Found: C, 50.32; H, 7.07; N, 0.05.

**2-(heptanoylthio)acetic acid (B4).** From: thioglycolic acid, triethylamine, and heptanoyl chloride. Appearance: Colorless oil. Yield: 12%. ^1^H NMR (400 MHz, CDCl_3_) δ 0.88 (t, *J* = 6.7 Hz, 3H, Alif), 1.35 (m, 2H, Alif), 1.68 (m, 2H, Alif), 2.66 (t, *J* = 7.5 Hz, 2H, Alif), 4.17 (q, *J* = 7.1 Hz, 2H, CH_2_–S) ppm. ^13^C NMR (101 MHz, CDCl_3_) δ = 13.98, 22.40, 28.47, 31.37, 47.52, 61.60, 170.33(–COOH), 199.39 (–COSR) ppm. Anal. Calcd for C_9_H_16_O_3_S (%): C, 52.92; H, 7.89; N, 0.00. Found: C, 53.22; H, 7.67; N, 0.06.

**2-((cyclopropanecarbonyl)thio)acetic acid (B5).** From: thioglycolic acid, triethylamine, and cyclopropanecarbonyl chloride. Appearance: Colorless oil. Yield: 25%. ^1^H NMR (400 MHz, CDCl_3_) δ 1.05 (m, 2H, Alif), 1.23 (m, 2H, Alif), 2.07 (m, 1H, Alif), 3.75 (d, *J* = 1.9 Hz, 2H, CH_2_–S) ppm. ^13^C NMR (101 MHz, CDCl_3_) δ = 11.68, 25.33, 61.64, 170.29 (–COOH), 198.87 (–COSR) ppm. Anal. Calcd for C_6_H_8_O_3_S (%): C, 44.99; H, 5.03; N, 0.00. Found: C, 45.11; H, 4.87; N, 0.09.

**2-((cyclobutanecarbonyl)thio)acetic acid (B6).** From: thioglycolic acid, triethylamine, and cyclobutanecarbonyl chloride. Appearance: Colorless oil. Yield: 34%. ^1^H NMR (400 MHz, CDCl_3_) δ 1.96 (m, 2H, Alif), 2.26 (m, 2H, Alif), 2.35 (m, 2H, Alif), 3.42 (m, 1H, Alif), 3.73 (s, 2H, CH_2_–S) ppm. ^13^C NMR (101 MHz, CDCl_3_) δ = 18.01, 25.97, 30.77, 37.80, 46.42, 174.54(–COOH), 199.60 (–COSR) ppm. Anal. Calcd for C_7_H_10_O_3_S (%): C, 48.26; H, 5.79; N, 0.00. Found: C, 45.07; H, 6.01; N, 0.12.

**2-((cyclopentanecarbonyl)thio)acetic acid (B7).** From: thioglycolic acid, triethylamine, and cyclopentanecarbonyl chloride. Appearance: Colorless. Yield: 31%. ^1^H NMR (400 MHz, CDCl_3_) δ 0.88 (t, *J* = 6.7 Hz, 3H, Alif), 1.35 (m, 2H, Alif), 1.68 (m, 2H, Alif), 2.66 (t, *J* = 7.5 Hz, 2H, Alif), 4.17 (q, *J* = 7.1 Hz, 2H, CH_2_–S) ppm. ^13^C NMR (101 MHz, CDCl_3_) δ = 25.12, 25.70, 30.14, 56.48, 61.51, 170.39 (–COOH), 202.77 (–COSR) ppm. Anal. Calcd for C_8_H_12_O_3_S (%): C, 51.05; H, 6.43; N, 0.00. Found: C, 50.88; H, 6.32; N, 0.05.

**2-((cyclohexanecarbonyl)thio)acetic acid (B8).** From: thioglycolic acid, triethylamine, and cyclohexanecarbonyl chloride. Appearance: Yellow oil. Yield: 34%. ^1^H NMR (400 MHz, CDCl_3_) δ 1.27 (m, 3H, Alif), 1.48 (m, 2H, Alif), 1.67 (m, 1H, Alif), 1.79 (m, 2H, Alif), 1.95 (m, 2H, Alif), 2.53 (m, 1H, Alif), 3.70 (s, 2H, CH_2_–S) ppm. ^13^C NMR (101 MHz, CDCl_3_) δ = 25.39, 25.54, 29.36, 30.72, 42.84, 52.26, 174.75 (–COOH), 201.15 (–COSR) ppm. Anal. Calcd for C_9_H_14_O_3_S (%): C, 53.44; H, 6.98; N, 0.00. Found: C, 53.19; H, 7.07; N, -0.02.

### 3.4. General Procedure for the Preparation of Selanyl Acetate Derivatives (**C1**–**C8**)

The reaction was carried out following the procedure used for the **A** series reaction. Briefly in a flask cooled with ice and nitrogen atmosphere, sodium hydrogen selenide was obtained, which was immediately reacted with methyl bromoacetic acid and the corresponding acid chlorides. THF (5 mL) was added due to the low solubility of these compounds. This reaction was stirred overnight at room temperature. After this time, the reaction was stopped, the organic fraction was filtered off and then the ethanol was evaporated under reduced pressure. Then, decantation was performed with H_2_O (3 × 30 mL) and DCM (3 × 30 mL). A silica gel chromatographic column with hexane/ethyl acetate (EtOH) in a 8:2 ratio was used as purification method. Oily liquid compounds were obtained, whose purity and structural characteristics were confirmed by elemental analysis and NMR.

**Methyl 2-(butyrylselanyl)acetate (C1).** From: sodium hydrogen selenide, methyl-2-bromoacetate, and butyryl chloride. Appearance: Yellow oil. Yield: 14%. ^1^H NMR (400 MHz, CDCl_3_) δ 0.98 (t, *J* = 7.4 Hz, 3H, Alif), 1.73 (h, *J* = 7.4 Hz, 2H, Alif), 2.65 (t, *J* = 7.4 Hz, 2H, Alif), 3.65 (s, 2H, CH_2_–Se), 3.72 (s, 3H, CH_3_–O) ppm. ^13^C NMR (101 MHz, CDCl_3_) δ = 13.36, 18.87, 24.79, 49.29, 52.66, 170.81 (–COOCH_3_), 199.15 (–COSeR) ppm. ^77^Se NMR (76 MHz, CDCl_3_) δ 561.81 ppm. Anal. Calcd for C_7_H_12_O_3_Se (%): C, 37.68; H, 5.42; N, 0.00. Found: C, 38.00; H, 5.31; N, 0.09.

**Methyl 2-(pentanoylselanyl)acetate (C2).** From: sodium hydrogen selenide, methyl-2-bromoacetate, and pentanoyl chloride. Appearance: Yellow oil. Yield: 12%. ^1^H NMR (400 MHz, CDCl_3_) δ 0.98 (t, *J* = 7.4 Hz, 3H), 1.73 (h, *J* = 7.4 Hz, 2H), 2.65 (t, *J* = 7.4 Hz, 2H), 3.65 (s, 2H, CH_2_–Se), 3.72 (s, 3H, CH_3_–O) ppm. ^13^C NMR (101 MHz, CDCl_3_) δ = 13.68, 22.06, 27.52, 30.98, 43.41, 52.77, 169.38 (–COOCH_3_), 197.60 (–COSeR) ppm. ^77^Se NMR (76 MHz, CDCl_3_) δ 561.81 ppm. Anal. Calcd for C_8_H_14_O_3_Se (%): C, 40.51; H, 5.95; N, 0.00. Found: C, 40.73; H, 6.12; N, 0.07.

**Methyl 2-(hexanoylselanyl)acetate (C3).** From: sodium hydrogen selenide, methyl-2-bromoacetate, and hexanoyl chloride. Appearance: Colorless. Yield: 48%. ^1^H NMR (400 MHz, CDCl_3_) δ 1.61 (m, 2H, Alif), 1.71 (m, 2H, Alif), 1.89 (m, 4H, Alif), 3.07 (m, 1H, Alif), 3.64 (s, 2H, CH_2_–Se), 3.72 (s, 3H, CH_3_–O) ppm. ^13^C NMR (101 MHz, CDCl_3_) δ = 22.36, 31.34, 47.46, 52.60, 61.53, 170.74 (–COOCH_3_), 199.16 (–COSeR) ppm. ^77^Se NMR (76 MHz, CDCl_3_) δ 544.50 ppm. Anal. Anal. Calcd for C_9_H_16_O_3_Se (%): C, 43.03; H, 6.42; N, 0.00. Found: C, 42.86; H, 6.32; N, 0.07.

**Methyl 2-(heptanoylselanyl)acetate (C4).** From: sodium hydrogen selenide, methyl-2-bromoacetate, and cyclohexanecarbonyl chloride. Appearance: Yellow oil. Yield: 7%. ^1^H NMR (400 MHz, CDCl_3_) δ 0.88 (m, 3H, Alif), 1.31 (m, 6H, Alif), 1.68 (m, 2H, Alif), 2.66 (t, *J* = 7.5 Hz, 2H), 3.65 (s, 2H, CH_2_–Se), 3.72 (s, 3H, CH_3_–O) ppm. ^13^C NMR (101 MHz, CDCl_3_) δ = 14.00, 22.42, 25.43, 28.55, 30.97, 31.38, 43.69, 52.76, 169.36 (–COOCH_3_), 197.59 (–COSeR) ppm. ^77^Se NMR (76 MHz, CDCl_3_) δ 560.55 ppm. Anal. Calcd for C_10_H_18_O_3_Se (%): C, 45.29; H, 6.84; N, 0.00. Found: C, 44.94; H, 6.92; N, 0.04.

**Methyl 2-((cyclopropanecarbonyl)selanyl)acetate (C5).** From: sodium hydrogen selenide, methyl-2-bromoacetate, and cyclohexanecarbonyl chloride. Appearance: Yellow oil. Yield: 15%. ^1^H NMR (400 MHz, CDCl_3_) δ 1.05 (m, 2H, Alif), 1.27 (m, 2H, Alif), 2.14 (m, 1H, Alif), 3.67 (s, 2H, CH_2_–Se), 3.72 (s, 3H, CH_3_–O) ppm. ^13^C NMR (101 MHz, CDCl_3_) δ = 25.88, 29.96, 30.48, 30.99, 43.39, 52.74, 52.88, 169.49 (–COOCH_3_), 201.02 (–COSeR) ppm. ^77^Se NMR (76 MHz, CDCl_3_) δ 557.09 ppm. Anal. Calcd for C_7_H_10_O_3_Se (%): C, 38.02; H, 4.56; N, 0.00. Found: C, 38.14; H, 4.66; N, 0.07.

**Methyl 2-((cyclobutanecarbonyl)selanyl)acetate (C6).** From: sodium hydrogen selenide, methyl-2-bromoacetate, and cyclohexanecarbonyl chloride. Appearance: Yellow oil. Yield: 19%. ^1^H NMR (400 MHz, CDCl_3_) δ 1.05 (m, 2H, Alif), 1.27 (m, 2H, Alif), 2.14 (m, 1H, Alif), 3.67 (s, 2H, CH_2_–Se), 3.72 (s, 3H, CH_3_–O) ppm. ^13^C NMR (101 MHz, CDCl_3_) δ = 17.85, 24.43, 25.98, 49.80, 52.64, 170.87 (–COOCH_3_), 201.35 (–COSeR) ppm. ^77^Se NMR (76 MHz, CDCl_3_) δ 557.09 ppm. Anal. Calcd for C_8_H_12_O_3_Se (%): C, 40.86; H, 5.14; N, 0.00. Found: C, 41.01; H, 5.31; N, 0.03.

**Methyl 2-((cyclopentanecarbonyl)selanyl)acetate (C7).** From: sodium hydrogen selenide, methyl-2-bromoacetate, and cyclohexanecarbonyl chloride. Appearance: Yellow oil. Yield: 25%. ^1^H NMR (400 MHz, CDCl_3_) δ 1.61 (m, 2H, Alif), 1.71 (m, 2H, Alif), 1.89 (m, 4H, Alif), 3.07 (m, 1H, Alif), 3.64 (s, 2H, CH_2_–Se), 3.72 (s, 3H, CH_3_–O) ppm. ^13^C NMR (101 MHz, CDCl_3_) δ = 25.88, 29.96, 30.48, 30.99, 43.39, 52.74, 52.88, 169.49 (–COOCH_3_), 201.02 (–COSeR) ppm. ^77^Se NMR (76 MHz, CDCl_3_) δ 544.50 ppm. Anal. Calcd for C_9_H_14_O_3_Se (%): C, 43.38; H, 5.66; N, 0.00. Found: C, 43.10; H, 5.39; N, 0.03.

**Methyl 2-((cyclohexanecarbonyl)selanyl)acetate (C8).** From: sodium hydrogen selenide, methyl-2-bromoacetate, and cyclohexanecarbonyl chloride. Appearance: Yellow oil. Yield: 20%. ^1^H NMR (400 MHz, CDCl_3_) δ 1.27 (m, 4H, Alif), 1.48 (m, 2H, Alif), 1.65 (m, 1H, Alif), 1.80 (m, 2H, Alif), 1.98 (m, 2H, Alif), 2.55 (m, 1H, Alif), 3.62 (s, 2H, CH_2_–Se), 3.71 (s, 3H, CH_3_–O) ppm. ^13^C NMR (101 MHz, CDCl_3_) δ = 23.40, 24.36, 25.30, 25.58, 29.19, 52.64, 55.66, 61.54, 170.98 (–COOCH_3_), 203.15 (–COSeR) ppm. ^77^Se NMR (76 MHz, CDCl_3_) δ 543.31 ppm. Anal. Calcd for C_10_H_16_O_3_Se (%): C, 45.66; H, 6.13; N, 0.00. Found: C, 45.74; H, 6.26; N, 0.02.

### 3.5. General Procedure for the Preparation of Methyl Thioglycolate Derivatives (**D1**–**D8**)

The derivatives of the **D1**–**D8** series were obtained as a result of the reaction between methyl thioglycolate, TEA and the different acid chlorides using DCM as a solvent with a duration of 2 h. The purities of these compounds were checked by NMR.

**Methyl 2-(butyrylthio)acetate (D1).** From: methyl thioglycolate, triethylamine, and butyryl chloride. Appearance: Yellow oil. Yield: 45%. ^1^H NMR (400 MHz, CDCl_3_) δ 0.97 (t, *J* = 7.4 Hz, 3H, Alif), 1.72 (m, 2H, Alif), 2.60 (t, *J* = 7.4 Hz, 2H, Alif), 3.71 (s, 2H, CH_2_–S), 3.74 (s, 3H, CH_3_–O) ppm. ^13^C NMR (101 MHz, CDCl_3_) δ = 13.44, 19.04, 30.97, 45.50, 52.77, 169.37 (–COOCH_3_), 197.48 (–COSR) ppm. Anal. Calcd for C_7_H_12_O_3_S (%): C, 47.71; H, 6.86; N, 0.00. Found: C, 47.57; H, 6.69; N, 0.06.

**Methyl 2-(pentanoylthio)acetate (D2).** From: methyl thioglycolate, triethylamine, and pentanoyl chloride. Appearance: Yellow oil. Yield: 55%. ^1^H NMR (400 MHz, CDCl_3_) δ 0.92 (t, *J* = 7.7 Hz, 3H, Alif), 1.37 (m, 2H, Alif), 1.66 (m, 2H, Alif), 2.62 (t, *J* = 7.5 Hz, 2H, Alif), 3.71 (s, 2H, CH_2_–S), 3.74 (s, 3H, CH_3_–O) ppm. ^13^C NMR (101 MHz, CDCl_3_) δ = 13.68, 22.06, 27.52, 30.98, 43.41, 52.77, 169.38 (–COOCH_3_), 197.60 (–COSR) ppm. Anal. Calcd for C_8_H_14_O_3_S (%): C, 50.50; H, 7.42; N, 0.00. Found: C, 50.62; H, 7.39; N, 0.03.

**Methyl 2-(hexanoylthio)acetate (D3).** From: methyl thioglycolate, triethylamine, and hexanecarbonyl chloride. Appearance: Colorless oil. Yield: 36%. ^1^H NMR (400 MHz, CDCl_3_) δ 0.89 (m, 3H, Alif), 1.32 (m, 4H, Alif), 1.69 (m, 2H, Alif), 2.61 (t, *J* = 7.5 Hz, 2H, Alif), 3.71 (s, 2H, CH_2_–S), 3.74 (s, 3H, CH_3_–O) ppm. ^13^C NMR (101 MHz, CDCl_3_) δ = 13.85, 22.27, 25.15, 30.98, 31.03, 43.66, 52.77, 169.38(–COOCH_3_), 197.61 (–COSR) ppm. Anal. Calcd for C_9_H_16_O_3_S (%): C, 52.92; H,7.89; N, 0.00. Found: C, 53.14; H, 8.08; N, 0.04.

**Methyl 2-(heptanoylthio)acetate (D4).** From: methyl thioglycolate, triethylamine, and heptanecarbonyl chloride. Appearance: Colorless oil. Yield: 22%. ^1^H NMR (400 MHz, CDCl_3_) δ 0.88 (m, 3H, Alif), 1.31 (m, 6H, Alif), 1.68 (m, 2H, Alif), 2.61 (t, *J* = 7.5 Hz, 2H, Alif), 3.71 (s, 2H, CH_2_–S), 3.74 (s, 3H,CH_3_–O) ppm. ^13^C NMR (101 MHz, CDCl_3_) δ = 14.00, 22.42, 25.43, 28.56, 30.98, 31.39, 43.70, 52.76, 169.37 (–COOCH_3_), 197.61(–COSR) ppm. Anal. Calcd for C_10_H_18_O_3_S (%): C, 55.02; H, 8.31; N, 0.00. Found: C, 54.89; H, 8.19; N, 0.06.

**Methyl 2-((cyclopropanecarbonyl)thio)acetate (D5).** From: methyl thioglycolate, triethylamine, and cyclopropanecarbonyl chloride. Appearance: Yellow oil. Yield: 25%. ^1^H NMR (400 MHz, CDCl_3_) δ 1.01 (m, 2H, Alif), 1.21 (m, 2H, Alif), 2.05 (m, 1H, Alif), 3.73 (s, 2H, CH_2_–S), 3.74 (s, 3H, CH_3_–O) ppm. ^13^C NMR (101 MHz, CDCl_3_) δ = 11.27, 22.45, 31.00, 52.78, 169.39 (–COOCH_3_), 197.38 (–COSR) ppm. Anal. Calcd for C_7_H_10_O_3_S (%): C, 48.26; H, 5.79; N, 0.00. Found: C, 48.02; H, 5.95; N, 0.04.

**Methyl 2-((cyclobutanecarbonyl)thio)acetate (D6).** From: methyl thioglycolate, triethylamine, and cyclobutanecarbonyl chloride. Appearance: Colorless oil. Yield: 65%. ^1^H NMR (400 MHz, CDCl_3_) δ 1.95 (m, 2H, Alif), 2.25 (m, 2H, Alif), 2.36 (m, 2H, Alif), 3.41 (m, 1H, Alif), 3.71 (s, 2H, CH_2_–S), 3.74 (s, 3H. CH_3_–O) ppm. ^13^C NMR (101 MHz, CDCl_3_) δ = 18.01, 25.97, 30.78, 46.46, 52.78, 169.48 (–COOCH_3_), 199.48 (–COSR) ppm. Anal. Calcd for C_8_H_12_O_3_S (%): C, 51.05; H, 6.43; N, 0.00. Found: C, 49.88; H, 6.26; N, -0.04.

**Methyl 2-((cyclopentanecarbonyl)thio)acetate (D7).** From: methyl thioglycolate, triethylamine, and cyclopentanecarbonyl chloride. Appearance: Yellow oil. Yield: 31%. ^1^H NMR (400 MHz, CDCl_3_) δ 1.60 (m, 2H, Alif), 1.72 (m, 2H, Alif), 1.88 (m, 4H, Alif), 3.03 (m, 1H, Alif), 3.70 (s, 2H, CH_2_–S), 3.74 (s, 3H, CH_3_–O) ppm. ^13^C NMR (101 MHz, CDCl_3_) δ = 25.88, 30.48, 31.00, 52.75 (m), 52.89, 169.51 (–COOCH_3_), 201.04 (–COSR) ppm. Anal. Calcd for C_9_H_14_O_3_S (%): C, 53.44; H, 6.98; N, 0.00. Found: C, 53.09; H, 6.69; N, 0.04.

**Methyl 2-((cyclohexanecarbonyl)thio)acetate (D8).** From: methyl thioglycolate, triethylamine, and cyclohexanecarbonyl chloride. Appearance: Yellow oil. Yield: 45%. ^1^H NMR (400 MHz, CDCl_3_) δ 1.27 (m, 3H, Alif), 1.48 (m, 2H, Alif), 1.66 (m, 1H, Alif), 1.79 (m, 2H, Alif), 1.95 (m, 2H, Alif), 2.53 (m, 1H, Alif), 3.68 (s, 2H, CH_2_–S), 3.73 (m, 3H, CH_3_–O) ppm. ^13^C NMR (101 MHz, CDCl_3_) δ = 25.42, 25.57, 29.40, 30.71, 52.31, 52.75, 169.51 (–COOCH_3_), 201.06 (–COSR) ppm. Anal. Calcd for C_10_H_16_O_3_S (%): C, 55.53; H, 7.46; N, 0.00. Found: C, 55.71; H, 7.55; N, 0.05.

### 3.6. Biology

For all the biological assays, the requested purity for all the tested compounds was ≥95%. Since some of the purified compounds contained some solvent trace, the purity before the biological testing was assessed by elemental analysis. To ensure this purity, the maximal value accepted for the deviation of the carbon, hydrogen, and nitrogen percentages was ±0.4. The obtained carbon, hydrogen, and nitrogen percentage values for each compound before its evaluation are presented in chemistry part of Section 3.

#### 3.6.1. Parasite Culture

Promastigotes. *L. major* (clone VI, MHOM/IL/80) and *L. infantum* (clone, BCN-150) parasites were cultured at 26 °C under continuous shaking in M199 1× medium (Sigma, St. Louis, MO, USA) supplemented with heat-inactivated fetal bovine serum (FBS), 25 mM HEPES (pH 7), 0.1 mM adenine, 0.0005% (*w*/*v*) haemin, 0.0001% (*w*/*v*), 0.0005% (*w*/*v*) biotin, 100 IU/mL penicillin and 100 mg/mL penicillin. For each experiment, the culture medium was changed everyday in order to achieve parasite in exponential growth phase.

#### 3.6.2. Cell Culture

Human monocytic leukemia cell line THP-1 cells were cultured in RMPI 1640 medium (Gibco, Leiden, The Netherlands) supplemented with 10% heat-inactivated FBS, 5% penicillin/streptomycin, 1 mM HEPES, 2 mM glutamine at pH 7.2 at 36 °C and 5% CO_2_.

#### 3.6.3. Leishmanicidal Activity of Glycolic Derivatives against Promastigotes

In order to determine the leishmanicidal activity of the compounds obtained in this study, promastigotes of *L. major* and *L. infantum* strains were seeded in 96-well plates (3 ×·10^6^ parasite/mL) in exponential growth phase at increasing concentrations (1–500 μM) of the compounds and maintained at 26 °C. After 48 h of incubation, the IC_50_ was determined by MTT assay [26]. The absorbance was measured in a MultiskanEX photometric plate reader for microplates at 540 nm. Data were obtained from three independent experiments performed in triplicate.

#### 3.6.4. Cytotoxicity Studies in PMA-Differentiated THP-1 Macrophages

THP-1 cells were seeded at a concentration of 8 × 10^5^ cells/mL in 96-well plates and incubated for 24 h with phorbol 12-myristate 13-acetate (PMA) (10 ng/mL) supplemented RPMI 1640 (Gibco, Leiden, The Netherlands). After 24 h, the culture medium was removed, and the cells were treated with the synthesized compounds at different concentrations ranging from 1–500 μM at 37 °C and 5% CO_2_. After this time, the MTT assay was performed. The cytotoxic concentration values (CC_50_) were obtained by fitting the data to a sigmoid dose-inhibition curve using GraphPad Prism 7.0 software (GraphPad Software Inc., San Diego, CA, USA). MIL and PMN were used as reference drugs for comparison.

#### 3.6.5. Drug-Sinergy Studies

The fractional inhibitory concentration index (FICI) was used to describe the interaction between the lead compounds **A4**–**A6** and **A8** with AmB, PMN and MIL. The compounds were studied in vitro against *L. infantum* promastigotes after 48 h of treatment. For this purpose, different increasing concentration ratios (0.2×, 0.4×, 0.6×, 0.8×, 1×, 1.2×, 1.4×, 1.6×, 1.8× and 2× times the IC_50_ of the compounds) were established [27]. Finally, synergy was defined as FICI < 0.5, non-interaction as 0.5 < FICI < 4, and antagonism as FICI > 4. FICI values were obtained from four independent experiments.

#### 3.6.6. Theorical ADME and Lipinski Properties

Absorption, distribution, metabolism, excretion, and toxicity (ADME) parameters were calculated using pkCSM program (https://biosig.lab.uq.edu.au/pkcsm/prediction, accessed on 18 July 2023).

## 4. Conclusions

In conclusion, the obtained results suggest that the selanylacetic acid derivatives **A4**–**A6** and **A8** (IC_50_ below 40 µM) were the leaders in this study, as they showed the highest activity, being 2- and 5-fold more active than their S counterparts and more potent than MIL (IC_50_ = 31.9 µM) against *L. infantum* parasites. These compounds were obtained following a simple, modular, and efficient one-step synthetic procedure, which, when evaluated against PMA-differentiated THP-1 macrophages, showed low toxicity and high selectivity. However, the emergence of drug resistance constitutes a major challenge. In this context, drug combination studies were conducted with the reference drug AMB, MIL and PMN. Results showed that when combining fixed ratio solutions of compounds **A6** and **A8** with MIL, showed a potent synergistic interaction. Compound **A6** exhibited a reduction of the IC_50_ from 16.7 to 5.2 μM and compound **A8** a decrease in the IC_50_ from 39.9 to 12.39 µM. Miltefosine, the only available oral treatment against visceral leishmaniasis caused by *L. infantum*, has showed a similar effect presented MIL combined with **A6** and **A8** which showed, a decrease in the IC_50_ from 31.9 to 6.55–9.29 μM, against *L. infantum* promastigotes. These results suggest the possibility that the synergistic interaction observed between the **A6** and **A8** derivatives may be linked to complementary mechanisms of action that enhance the efficacy of the combination and offer a promising therapeutic approach for the treatment of visceral leishmaniasis. The interesting leishmanicidal activity, the affordability and simplicity of the synthetic process, highlight their innovative design and potential as a leishmanicidal agent. Finally, further studies are required to elucidate the mechanisms of action and evaluate their efficacy during in vivo studies.

## Figures and Tables

**Table 1:** Cytotoxic activity (CC_50_) of the lead compounds (**A4**, **A5**, **A6**, and **A8**) and standard drugs (MIL, PMN) against THP-1 cells after 48 h of treatment.

	THP-1 Cells	SI	
Ref.	CC_50_ (μM)	*L. major*	*L. infantum*
**A4**	173.4 ± 8.6	3	6
**A5**	211.3 ± 9.3	2	9
**A6**	178.2 ± 10.2	3	11
**A8**	146.8 ± 12.5	2	5
**MIL**	13.3 ± 2.8	0.2	0.4
**PMN**	39.0 ± 13.7	0.6	2.2

**Table 2:** Fractional inhibitory concentrations of 50% overall mean (ƩFICI_50_) of the lead compounds (**A4**–**A6** and **A8**) and the reference drugs (AMB, MIL, and PMN) against *L. infantum* promastigotes.

A4			A5		
*L. infantum*			*L. infantum*		
Combination	ƩFICI_50_	Effect	Combination	ƩFICI_50_	Effect
**AmB**	0.71	Non interaction	**AmB**	0.70	Non interaction
**MIL**	0.68	Non interaction	**MIL**	0.98	Non interaction
**PMN**	0.68	Non interaction	**PMN**	0.61	Non interaction
**A6**			**A8**		
***L. infantum***			***L. infantum***		
**Combination**	**ƩFICI_50_**	**Effect**	**Combination**	**ƩFICI_50_**	**Effect**
**AmB**	0.70	Non interaction	**AmB**	0.52	Non interaction
**MIL**	0.44	Synergy	**MIL**	0.40	Synergy
**PMN**	0.54	Non interaction	**PMN**	0.52	Non interaction

**Table 3:** The absorption, distribution, metabolism, and excretion (ADME) properties of the lead compounds and the reference drug MIL and PMN.

Ref.	^a^ MW (g/mol)	^b^ LogP	^c^ OHNH Donors	^d^ ON Acceptors	^e^ Lipinski Violation	^f^ GI Absorption	^g^ Water Solubility	^h^ Caco2	^i^ Skin Sensitization	^j^ Skin Permeability
**A4**	251.18	1.69	1	2	0	High	−2.19	1.43	No	−2.53
**A5**	207.09	0.13	1	2	0	High	−0.92	1.17	No	−2.72
**A6**	221.11	0.52	1	2	0	High	−1.35	1.19	No	−2.67
**A8**	249.17	1.30	1	2	0	High	−2.20	1.24	No	−2.58
**MIL**	407.57	0.26	0	4	0	Low	−6.15	1.05	Yes	−2.72
**PMN**	615.63	−8.86	13	19	3	Low	−2.38	−0.70	No	−2.74

^a^ MW, molecular weight (g/mol); ^b^ LogP, octanol/water partition coefficient; ^c^ OHNH donors, number of protons able to act as hydrogen bond donors; ^d^ ON acceptors, number of protons capable of acting as hydrogen bond acceptors; ^e^ Lipinski’s rule of five; ^f^ GI absorption, human intestinal absorption; ^g^ Water solubility, amount of the compound that dissolves in water expressed as log mol/L; ^h^ Caco-2, apparent permeability of the Caco-2 cells, values > 0.90 means high permeability. ^i^ Skin sensitization, potential adverse effect of a product applied to the skin. ^j^ Skin permeability, ability of to pass through the different layers of the skin, values >−2.5 means low skin permeability.

## Data Availability

The raw data supporting the conclusions of this manuscript are available to any qualified researcher.

## Supplementary Materials

The following supporting information can be downloaded at: https://www.mdpi.com/article/10.3390/molecules28155845/s1.

## Sample Availability

Samples of the compounds **A1**–**A8**, **B1**–**B8**, **C1**–**C2** and **D1**–**D8** are available from authors.
