# Supplementary material for: Exploring Novel Drug Combinations: The Therapeutic Potential of Selanyl Derivatives for Leishmania Treatment
Source: Molecules. 2023 Aug 3;28(15):5845. doi: 10.3390/molecules28155845 (PMC10420963; doi:10.3390/molecules28155845)
Supplement: Supplementary file 1 [file molecules-28-05845-s001.zip › molecules-2487165-supplementary.pdf]

Suplementary material

*Chemical characterization – NMR spectra.

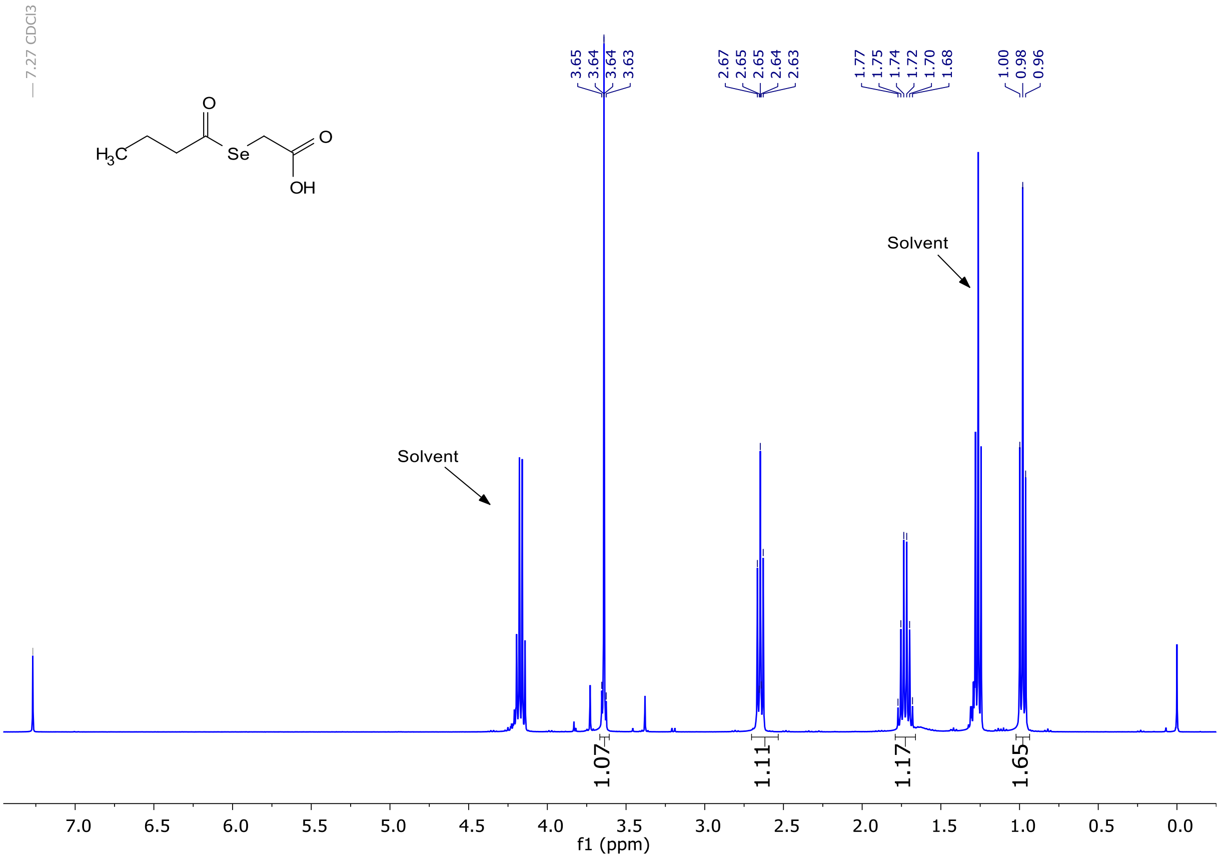

*Figure S1. ^{1}H-NMR spectrum of compound **A1**.*

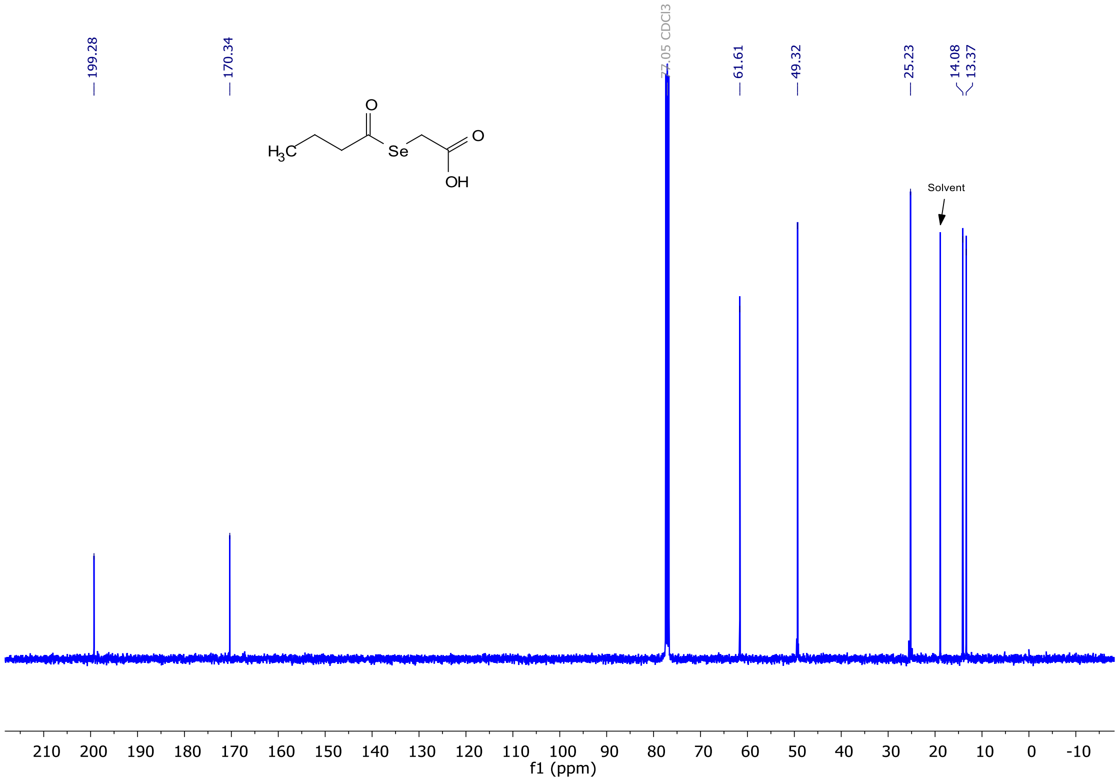

*Figure S2. ^{13}C-NMR spectrum of compound **A1**.*

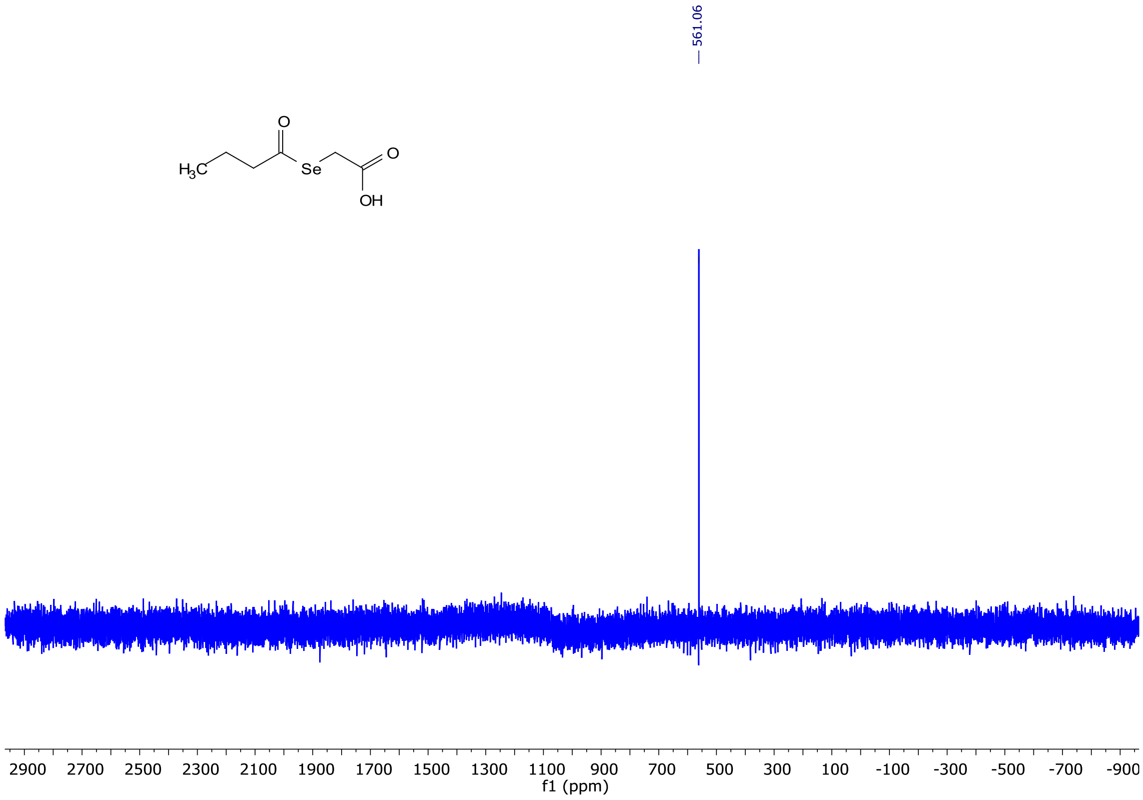

*Figure S3. ^{77}Se-NMR spectrum of compound **A1**.*

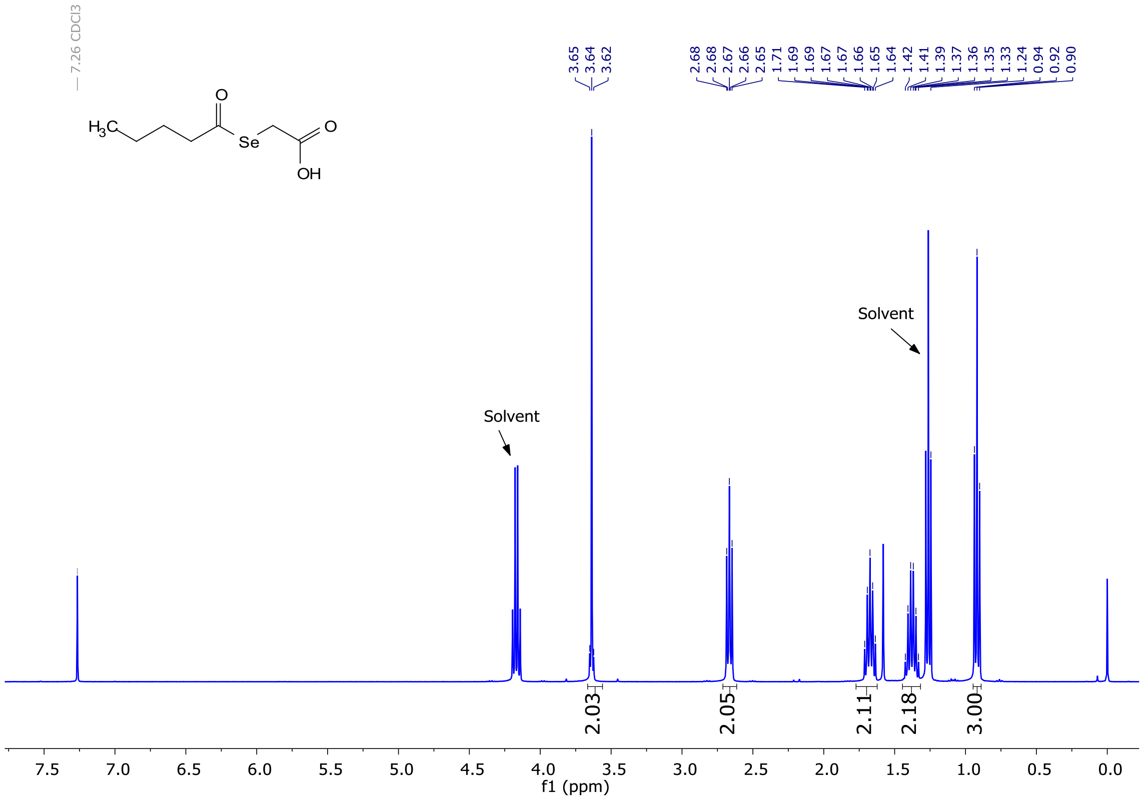

*Figure S4. 1H-NMR spectrum of compound **A2**.*

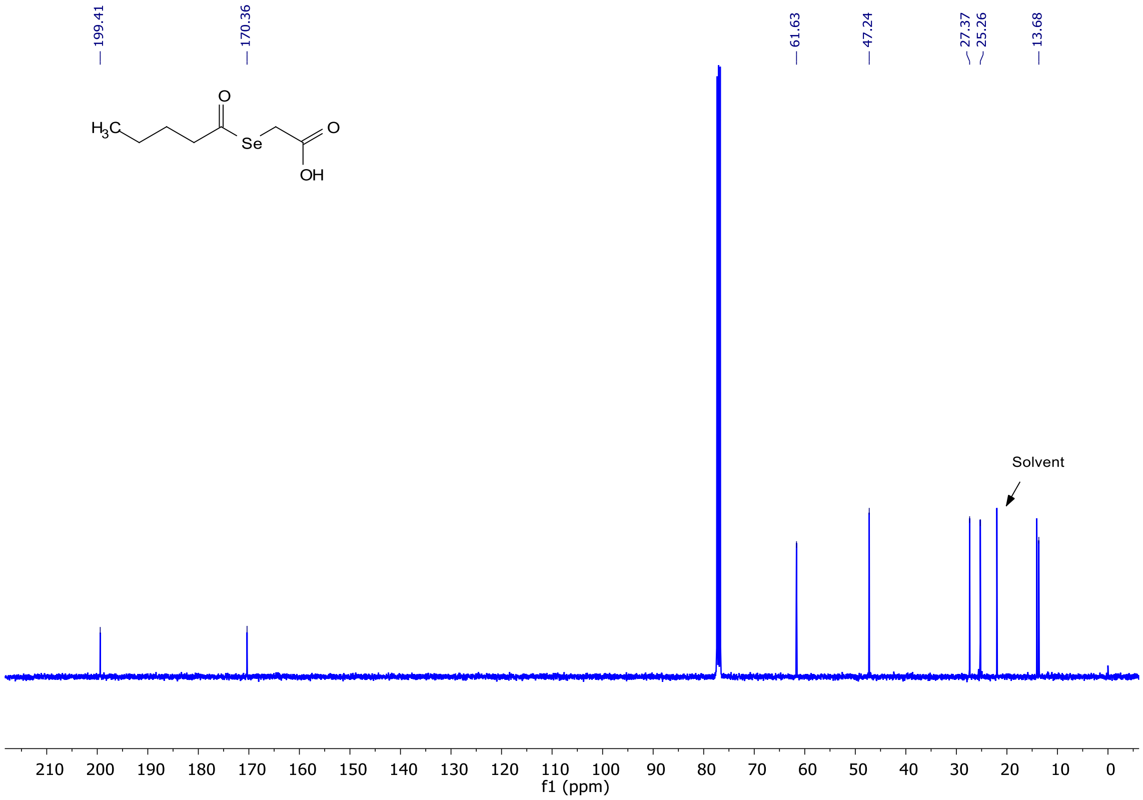

*Figure S5. 13C-NMR spectrum of compound **A2**.*

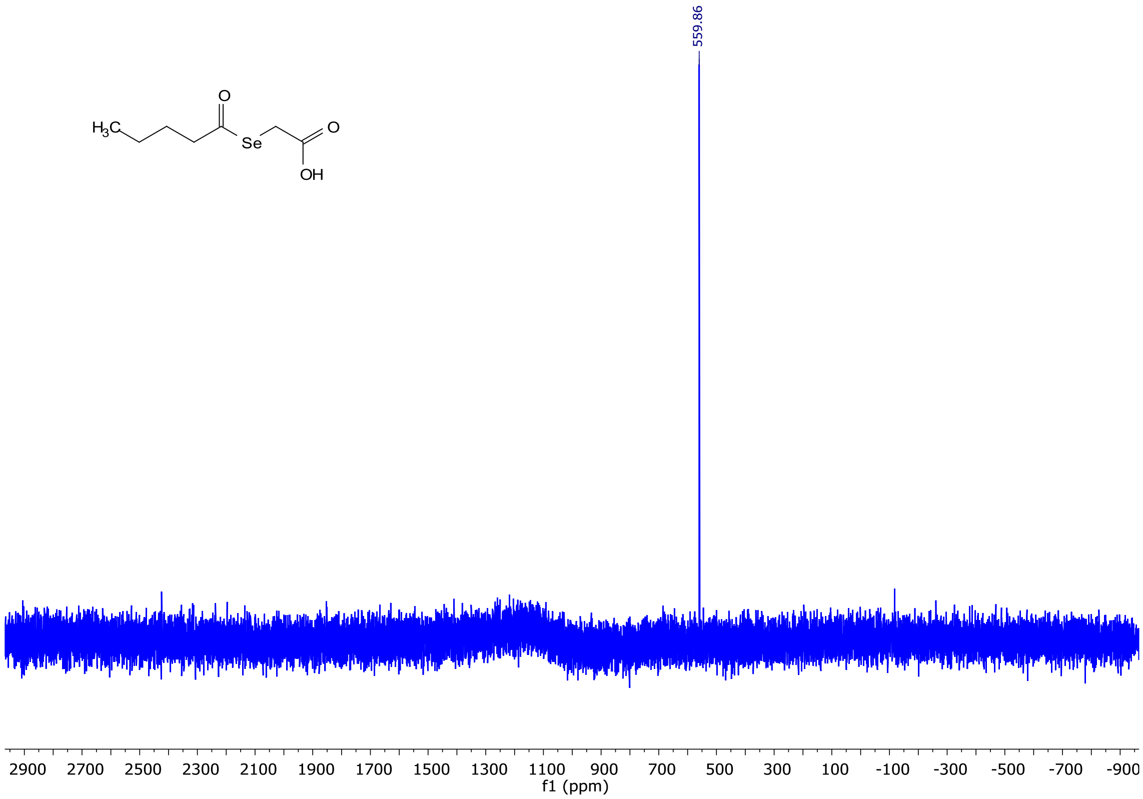

*Figure S6. ^{77}Se-NMR spectrum of compound **A2**.*

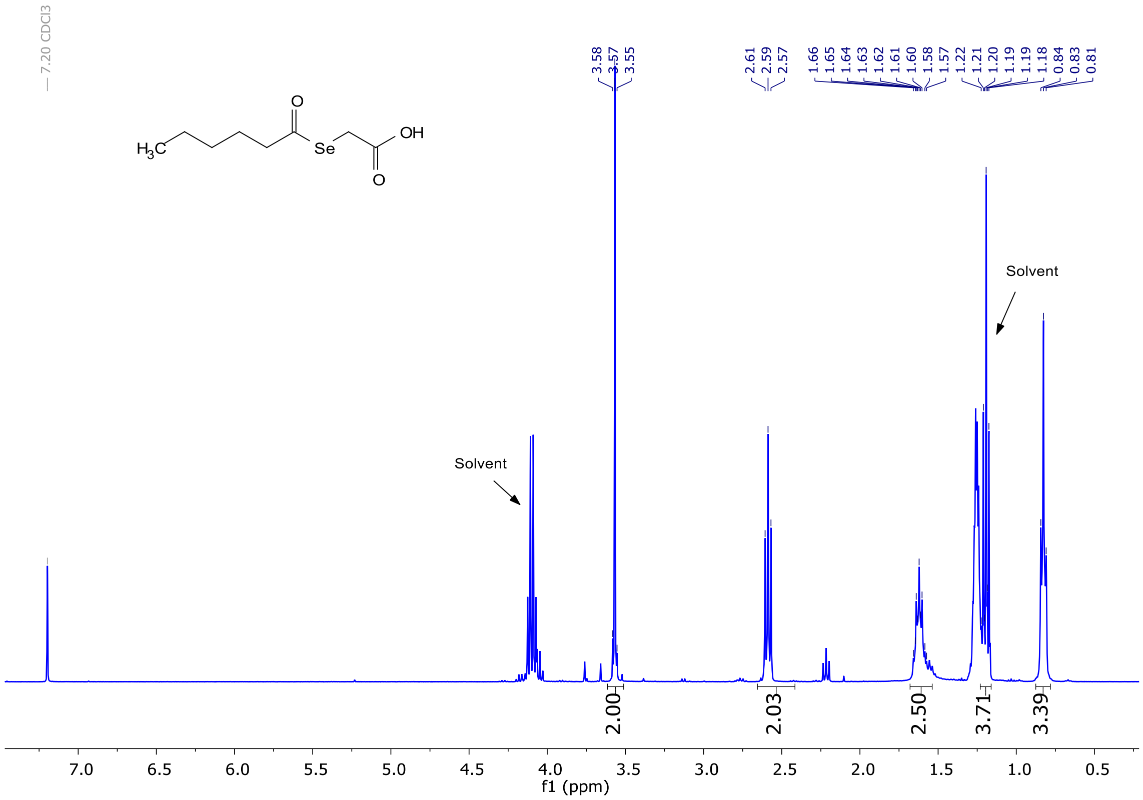

*Figure S7. ^{1}H-NMR spectrum of compound **A3**.*

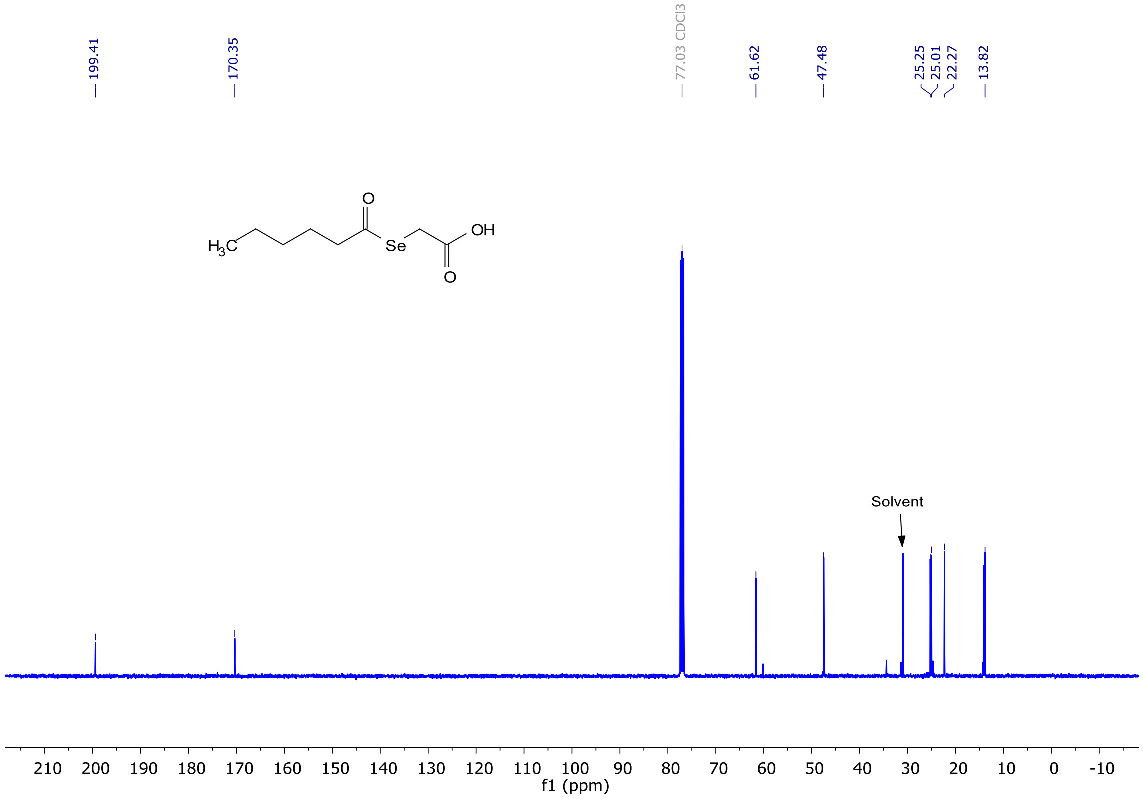

*Figure S8. ^{13}C-NMR spectrum of compound **A3**.*

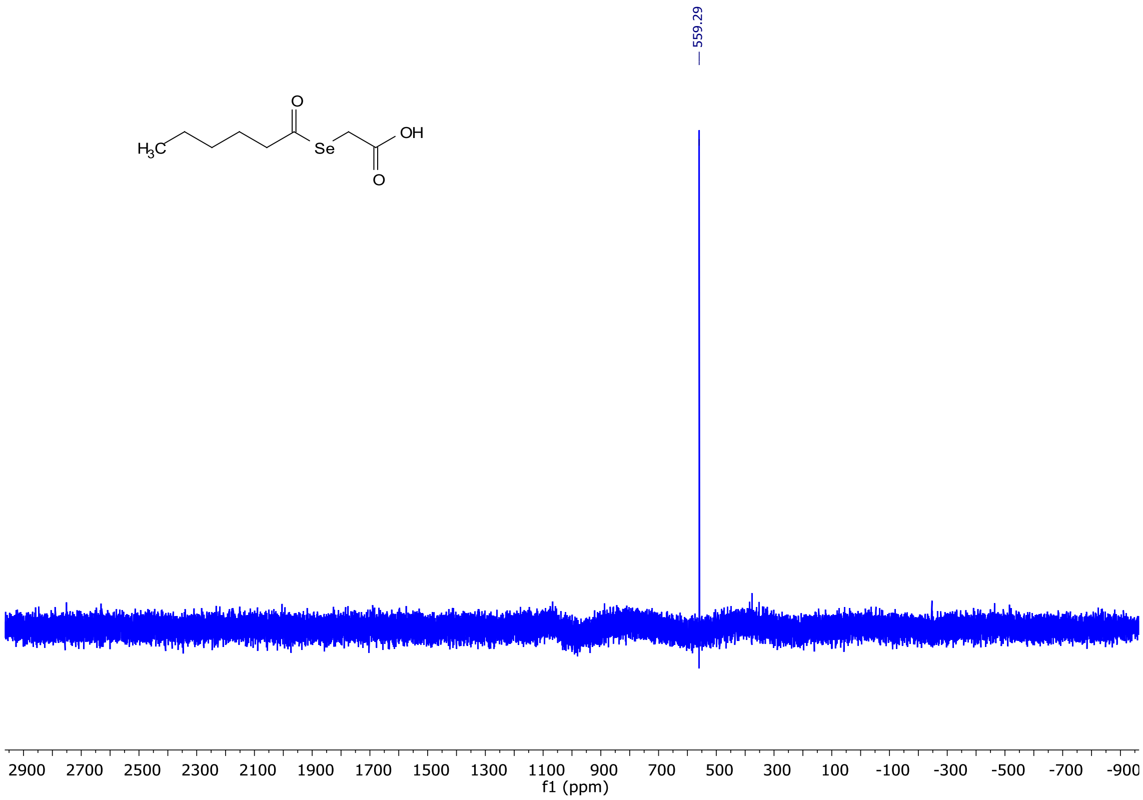

*Figure S9. ^{77}Se-NMR spectrum of compound **A3**.*

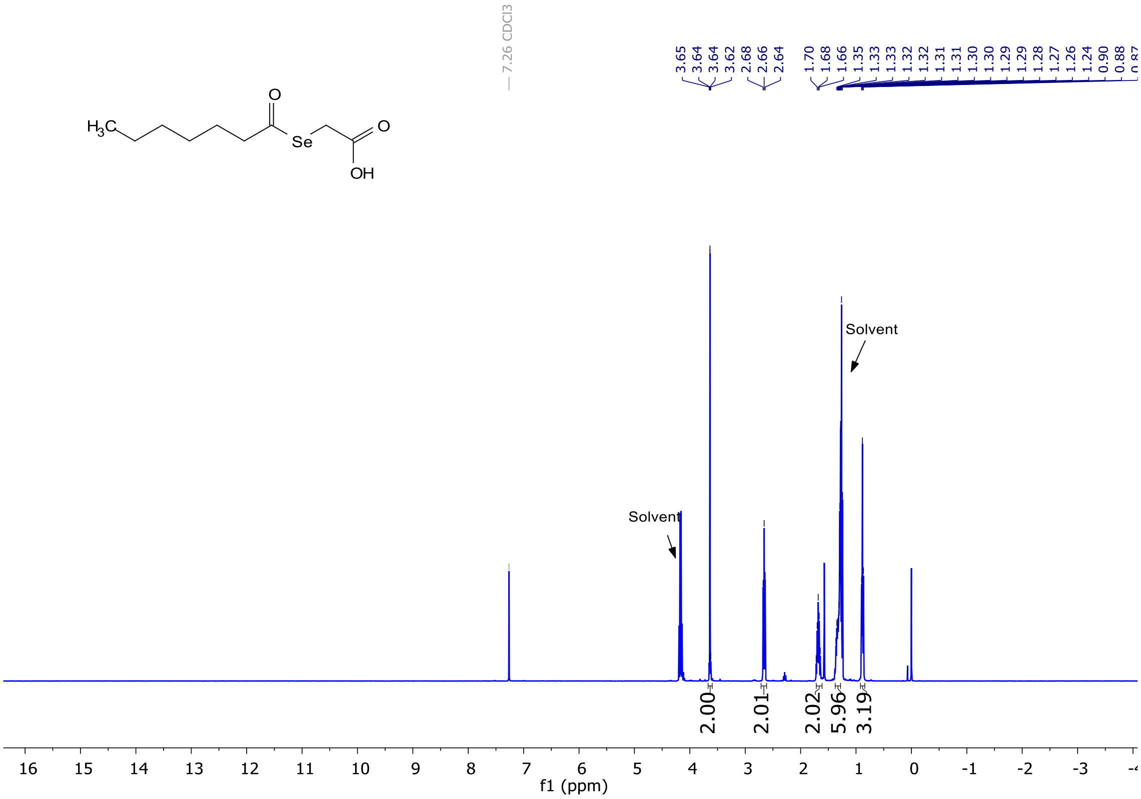

*Figure S10. ^{1}H-NMR spectrum of compound **A4**.*

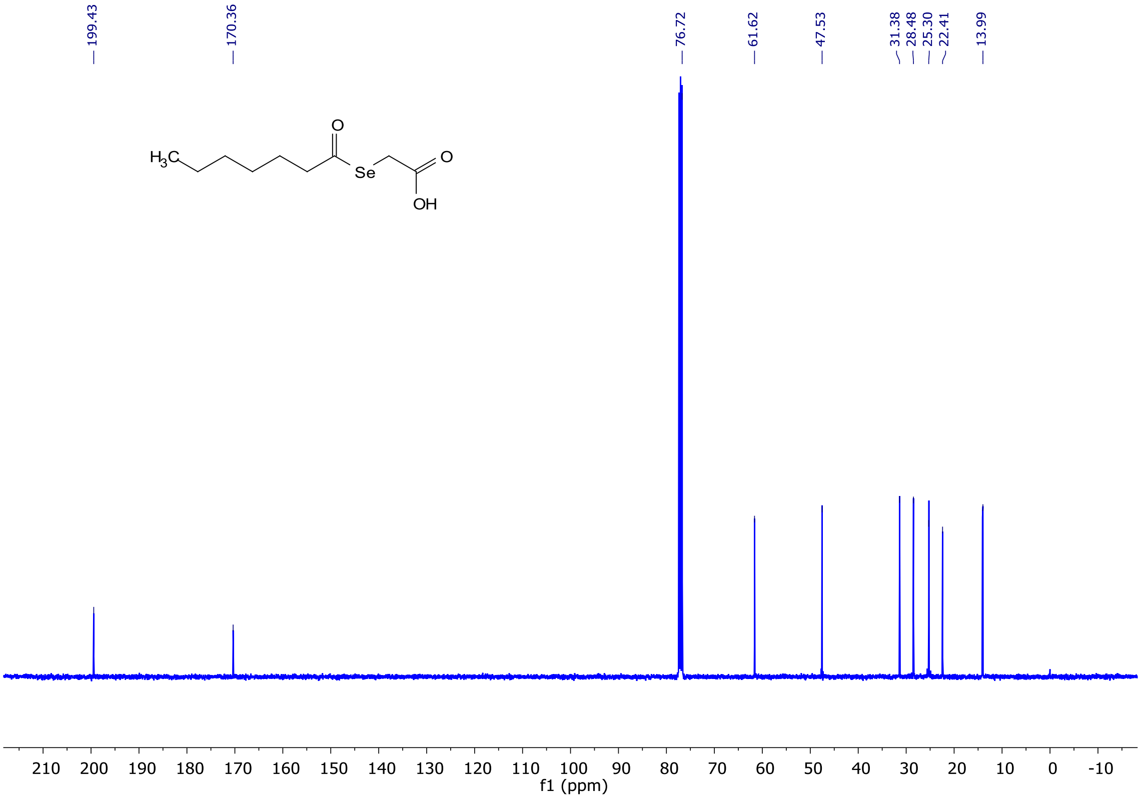

*Figure S11. ^{13}C-NMR spectrum of compound **A4**.*

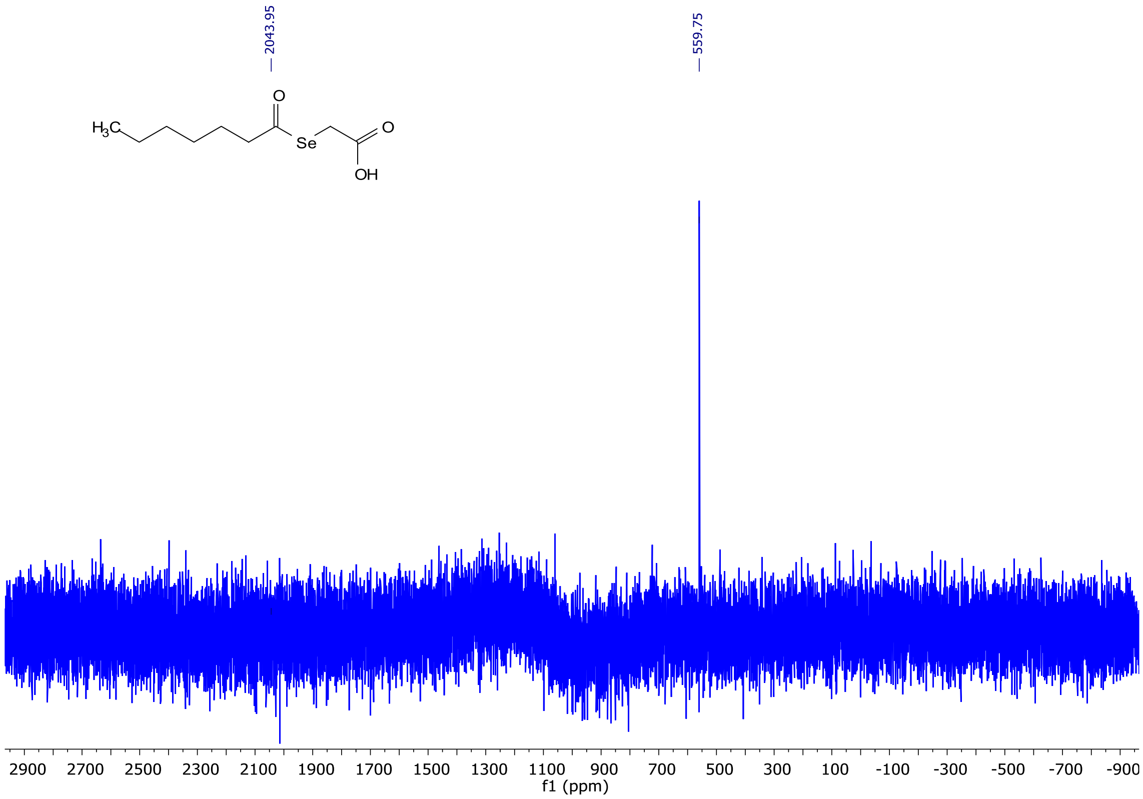

Figure S12. ^{77}*Se-NMR spectrum of compound* ***A4.***

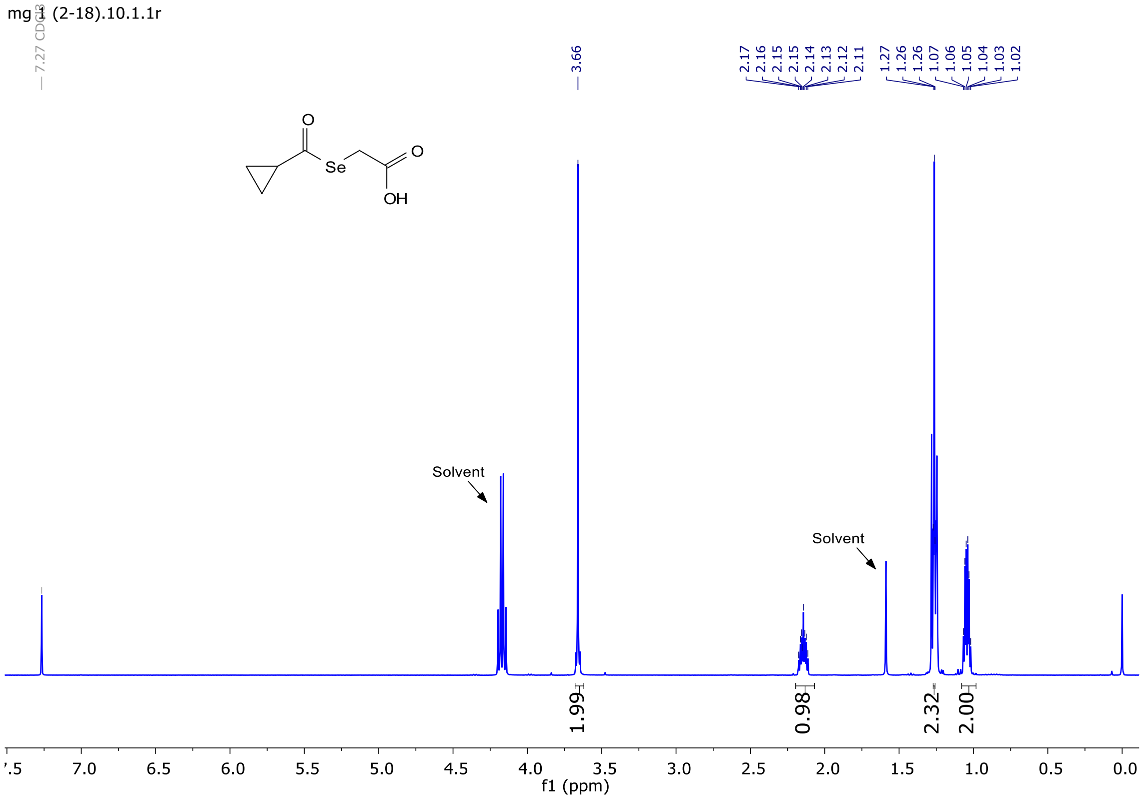

Figure S13. ^{1}*H-NMR spectrum of compound* ***A5.***

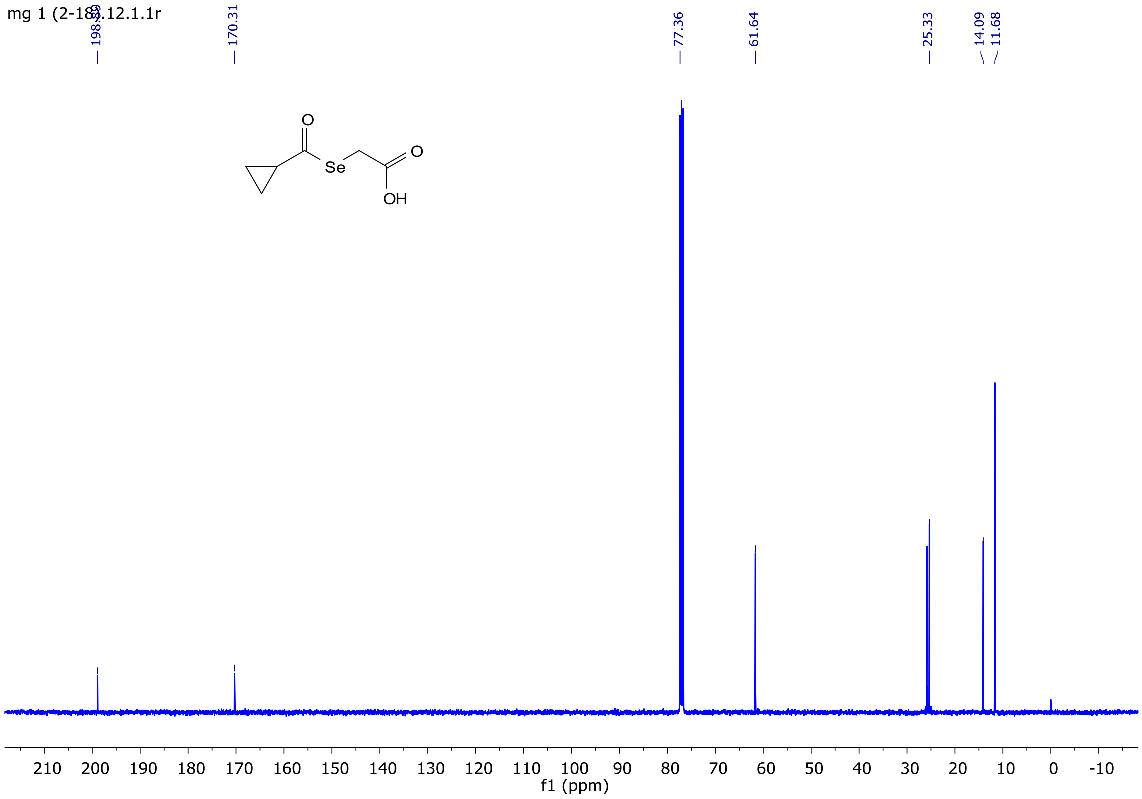

*Figure S14. ^{13}C-NMR spectrum of compound **A5**.*

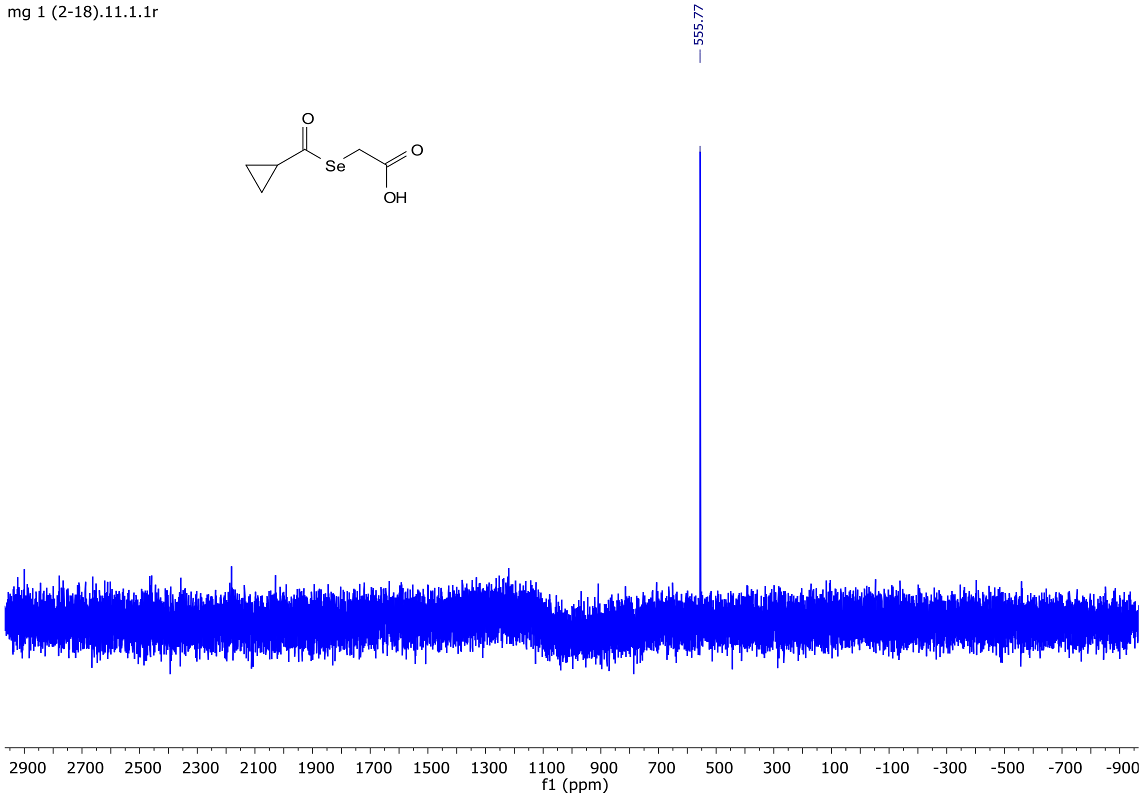

*Figure S15. ^{77}Se-NMR spectrum of compound **A5.***

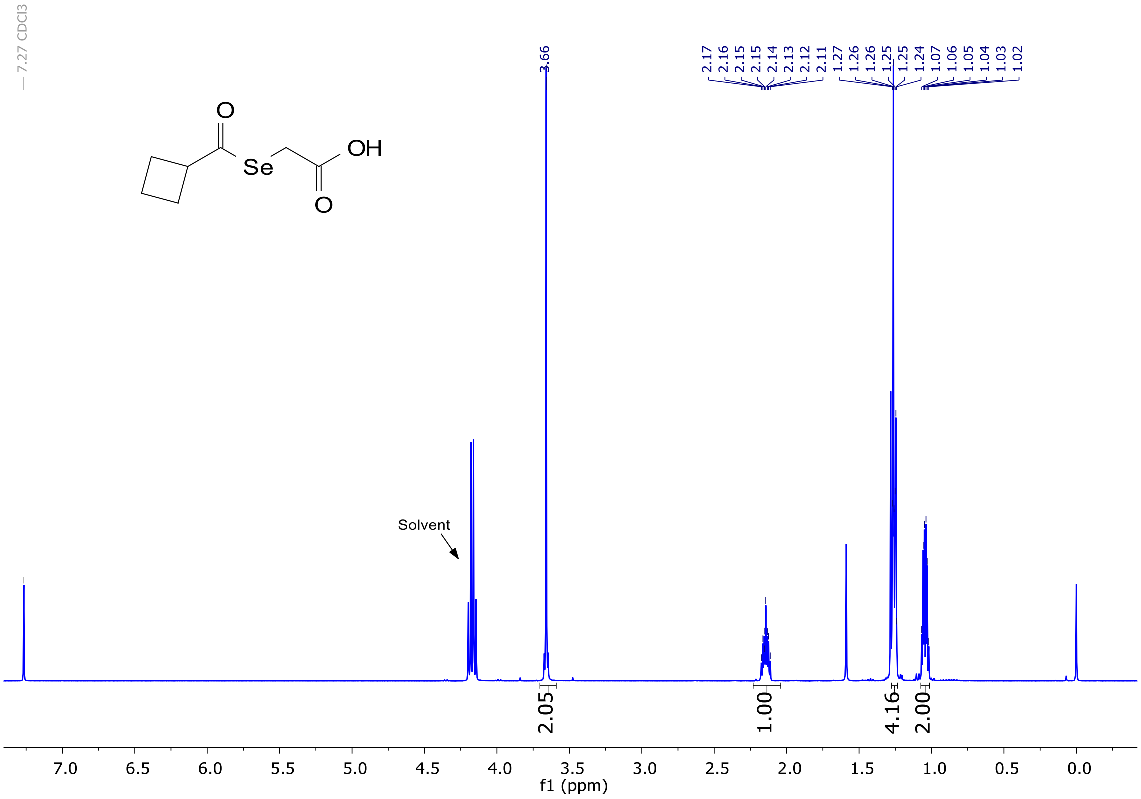

Figure S16. ^{1}H-NMR spectrum of compound ***A6****.*

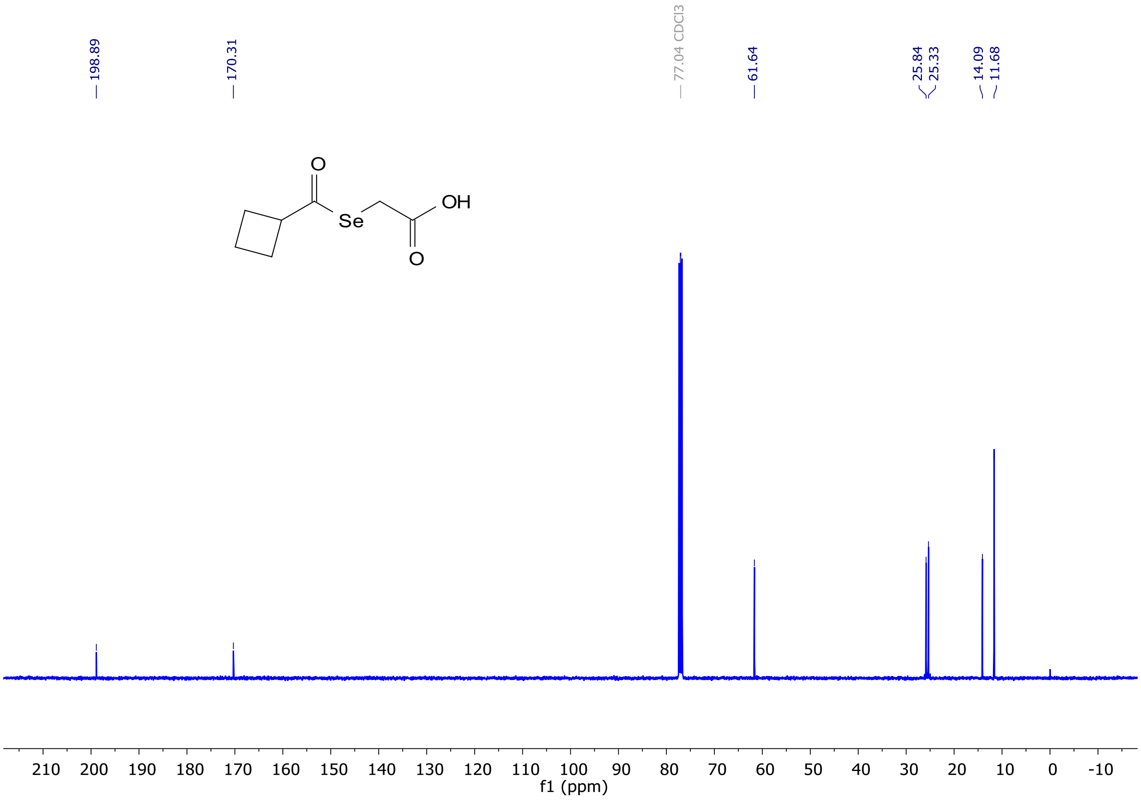

Figure S17. ^{13}C-NMR spectrum of compound ***A6****.*

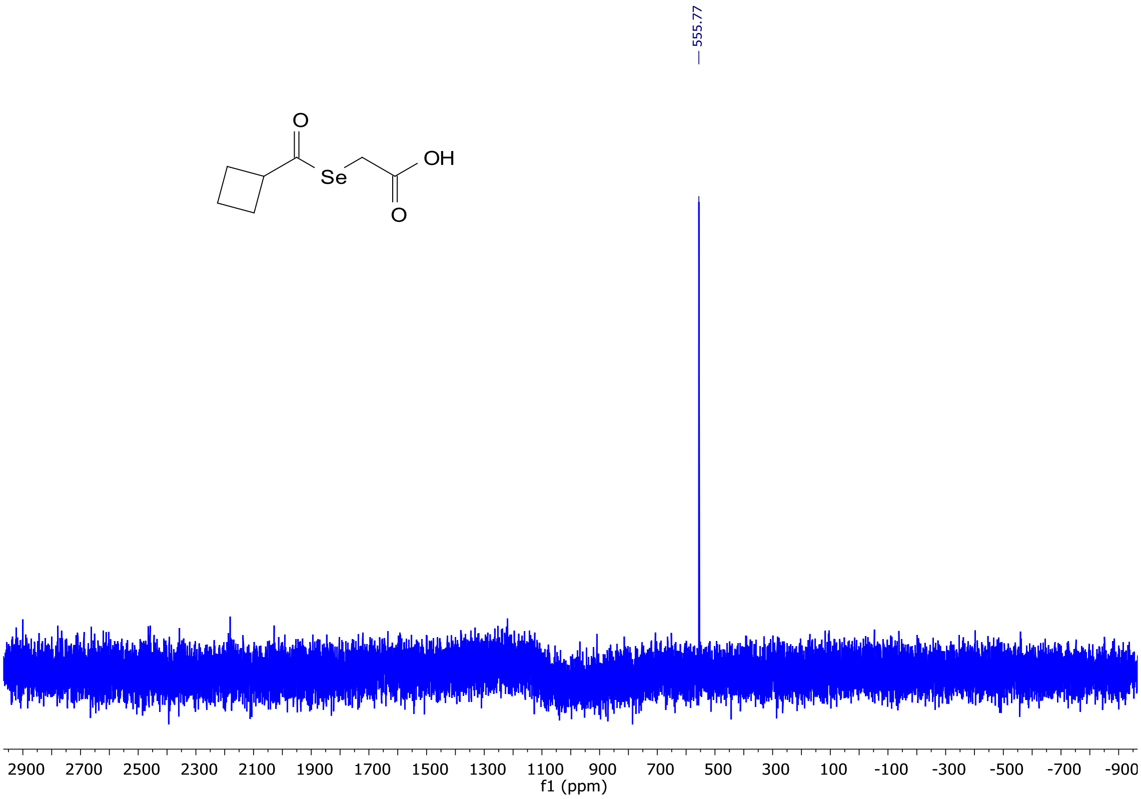

Figure S18. ^{77}Se-NMR spectrum of compound ***A6.***

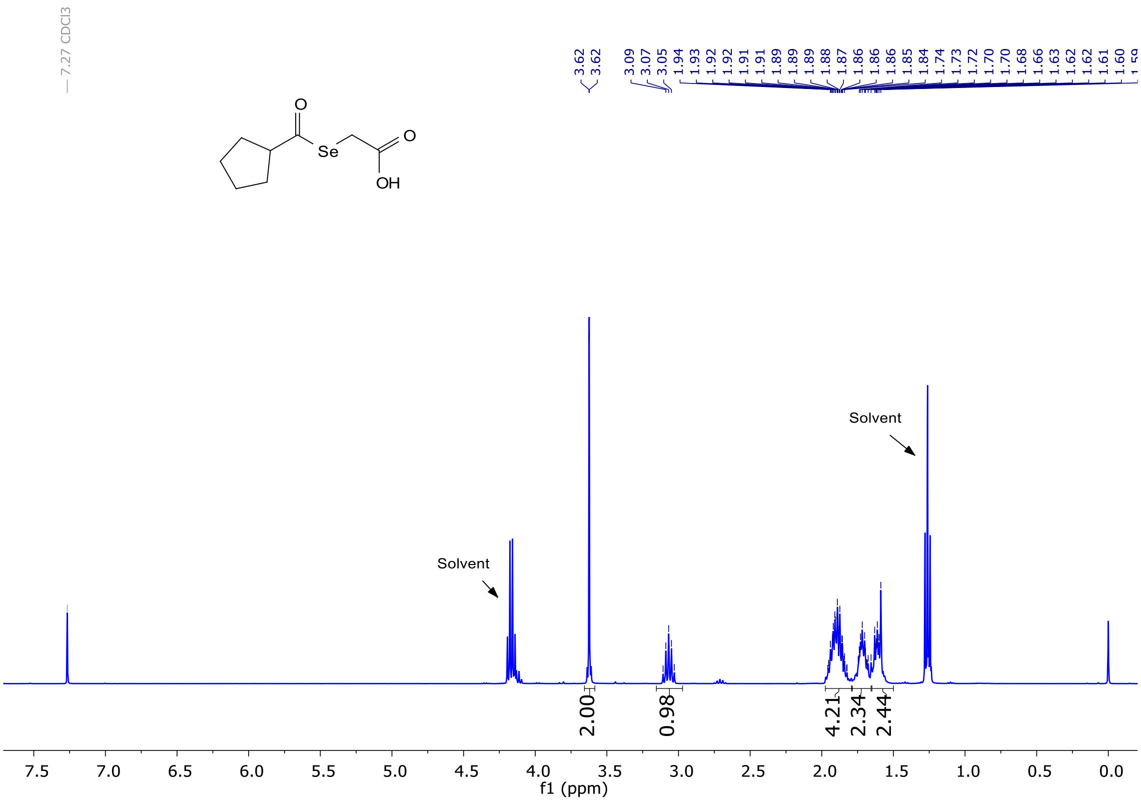

Figure S19. ^{1}H-NMR spectrum of compound ***A7****.*

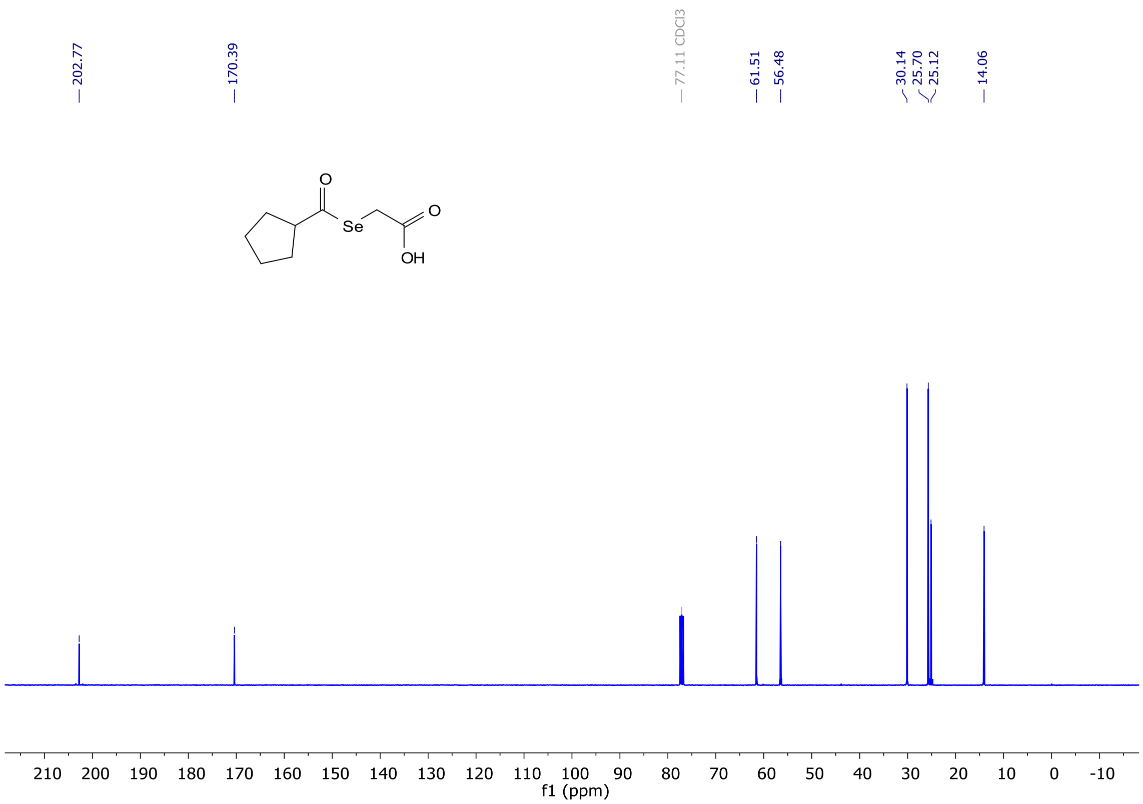

*Figure S20. ^{13}C-NMR spectrum of compound **A7**.*

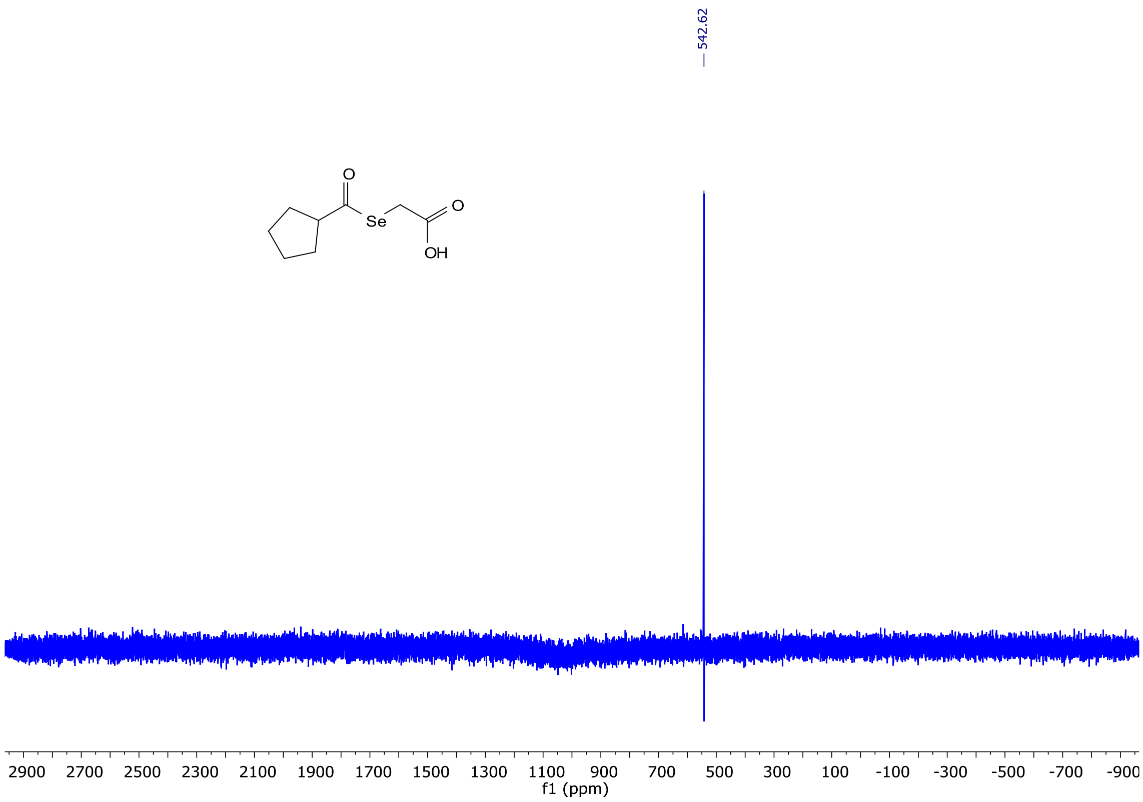

*Figure S21. ^{77}Se-NMR spectrum of compound **A7.***

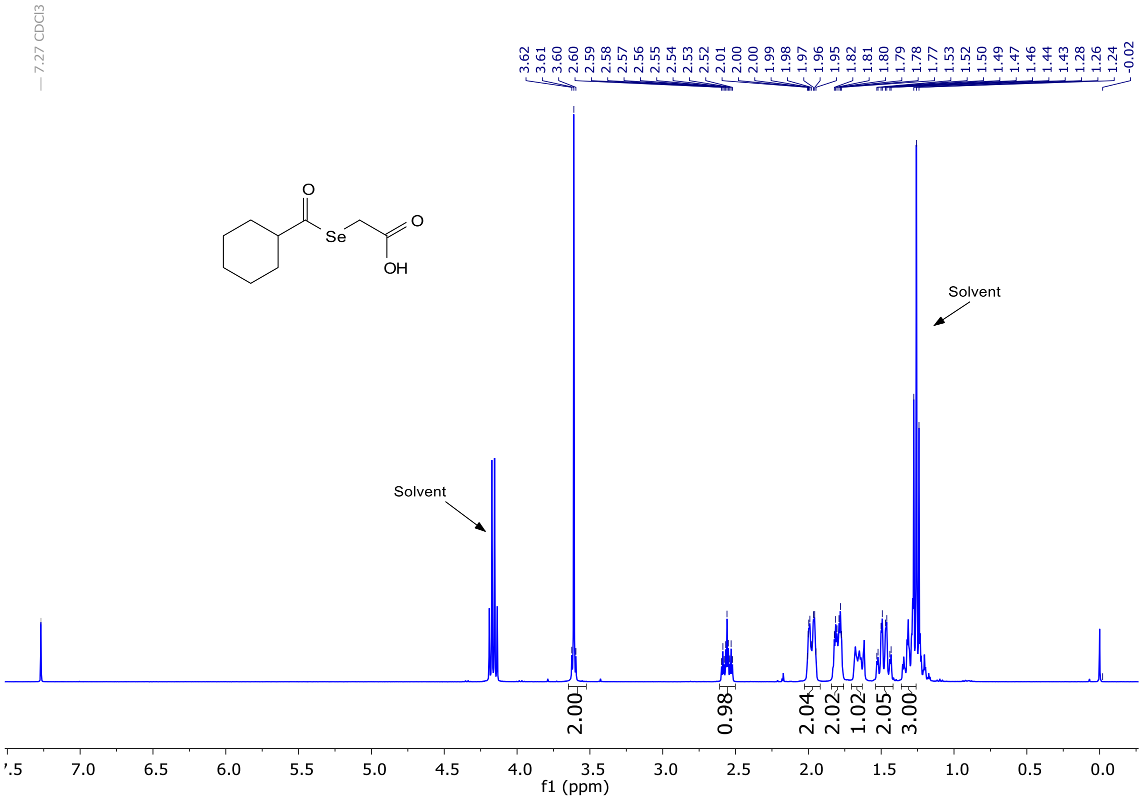

Figure S22. ^{1}H-NMR spectrum of compound ***A8.***

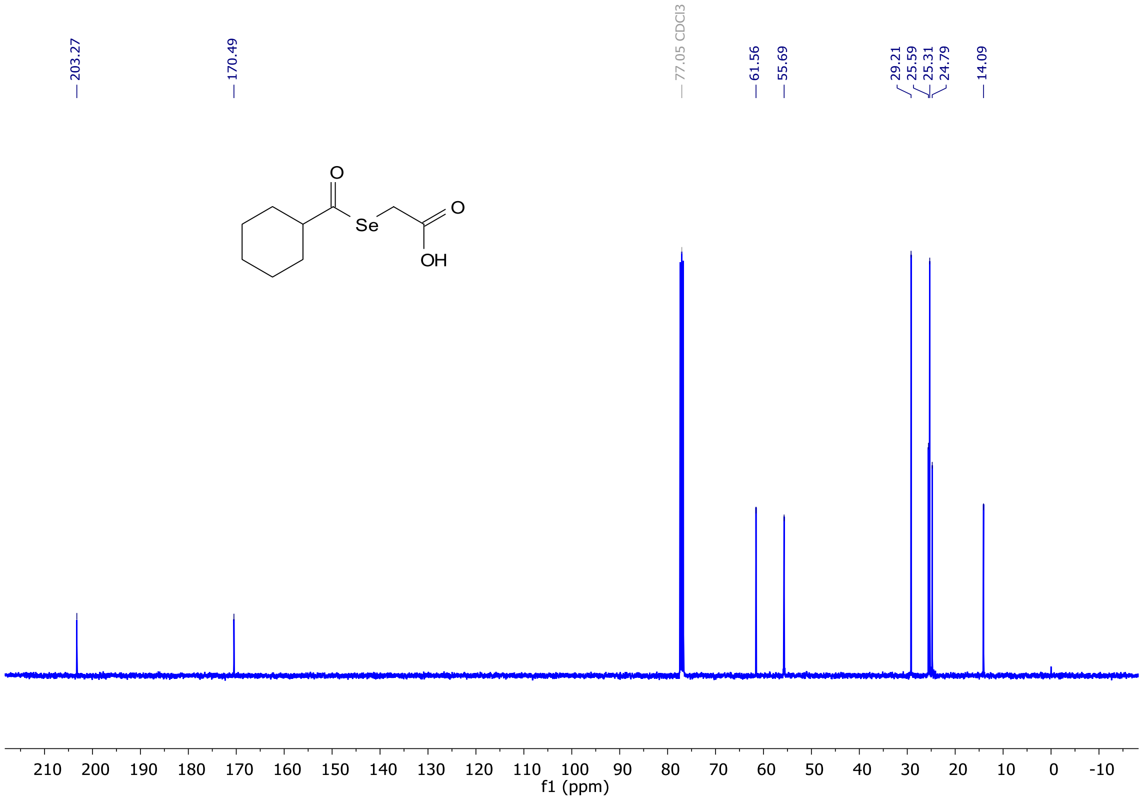

Figure S23. ^{13}C-NMR spectrum of compound ***A8***.

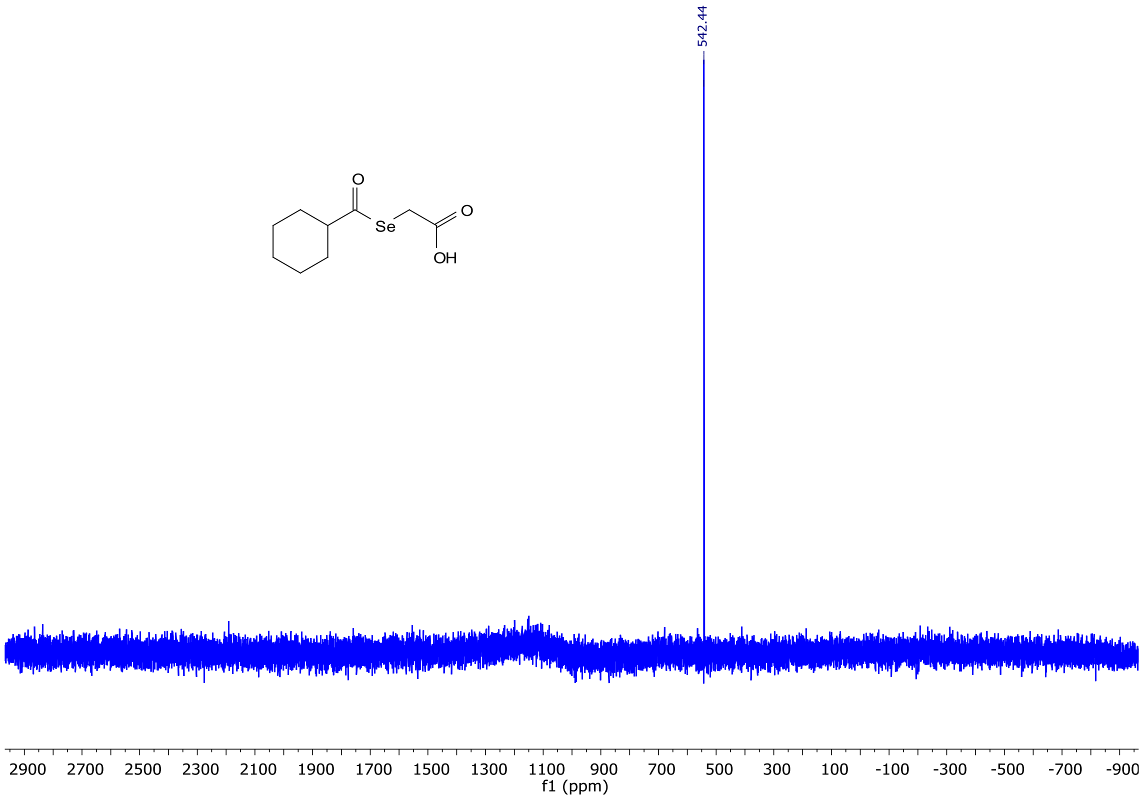

Figure S24. ^{77}Se-NMR spectrum of compound ***A8.***

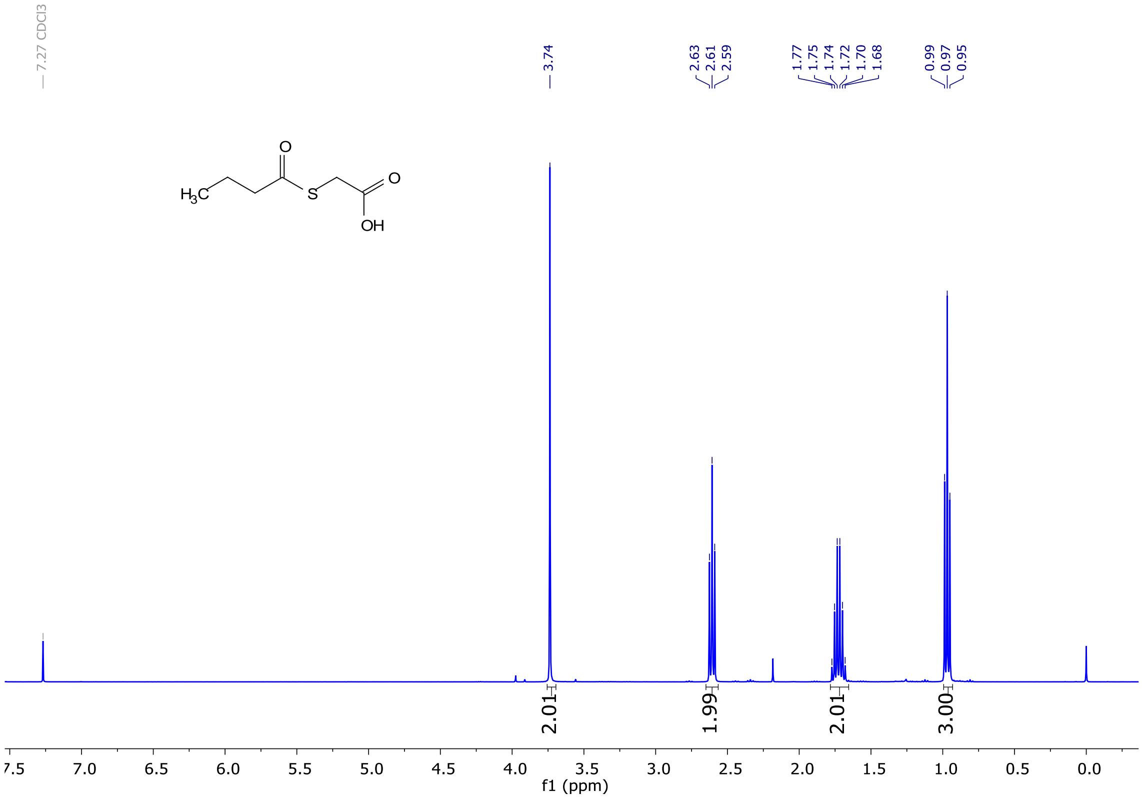

Figure S25. ^{1}H-NMR spectrum of compound ***B1.***

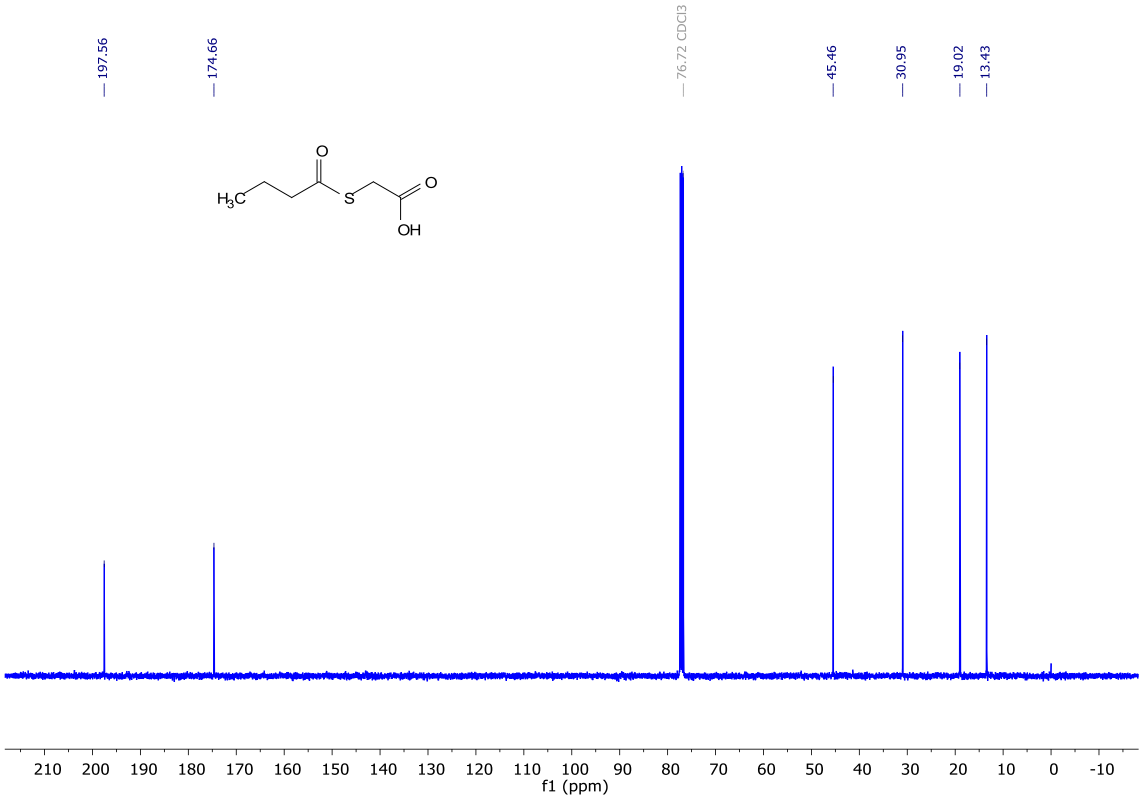

*Figure S26. ^{13}C-NMR spectrum of compound **B1**.*

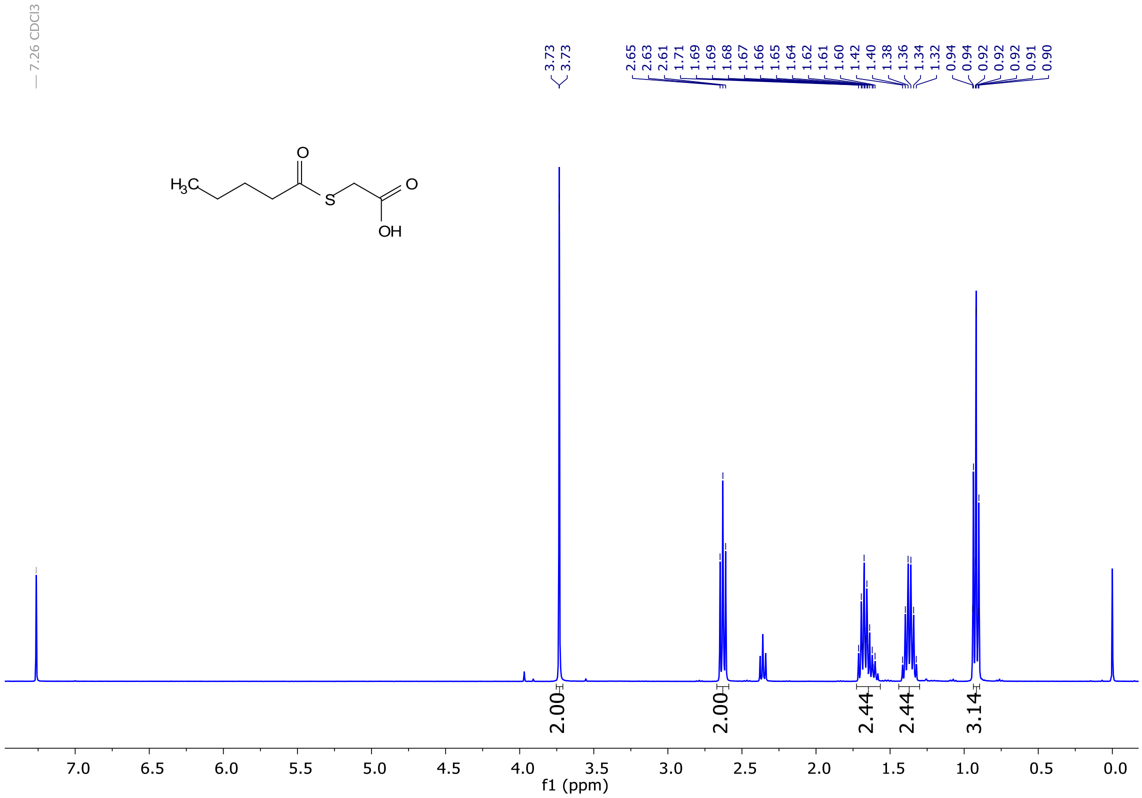

*Figure S27. ^{1}H-NMR spectrum of compound **B2.***

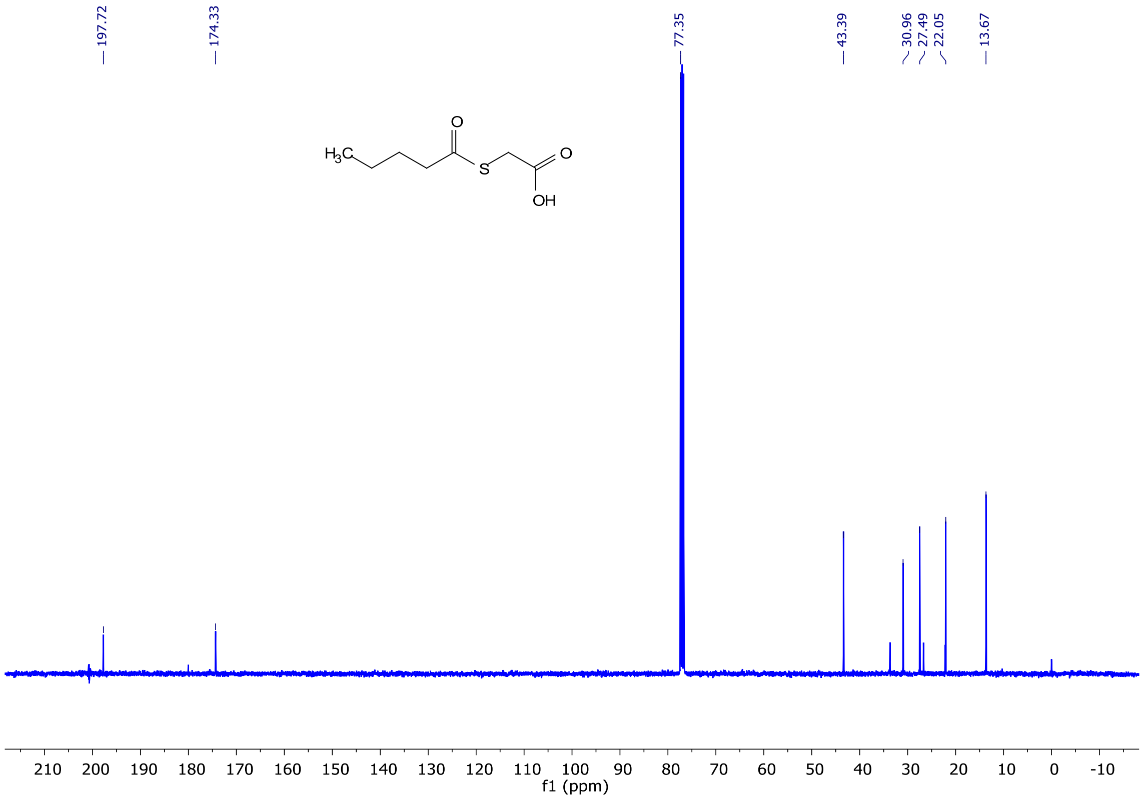

*Figure S28. ^{13}C-NMR spectrum of compound **B2**.*

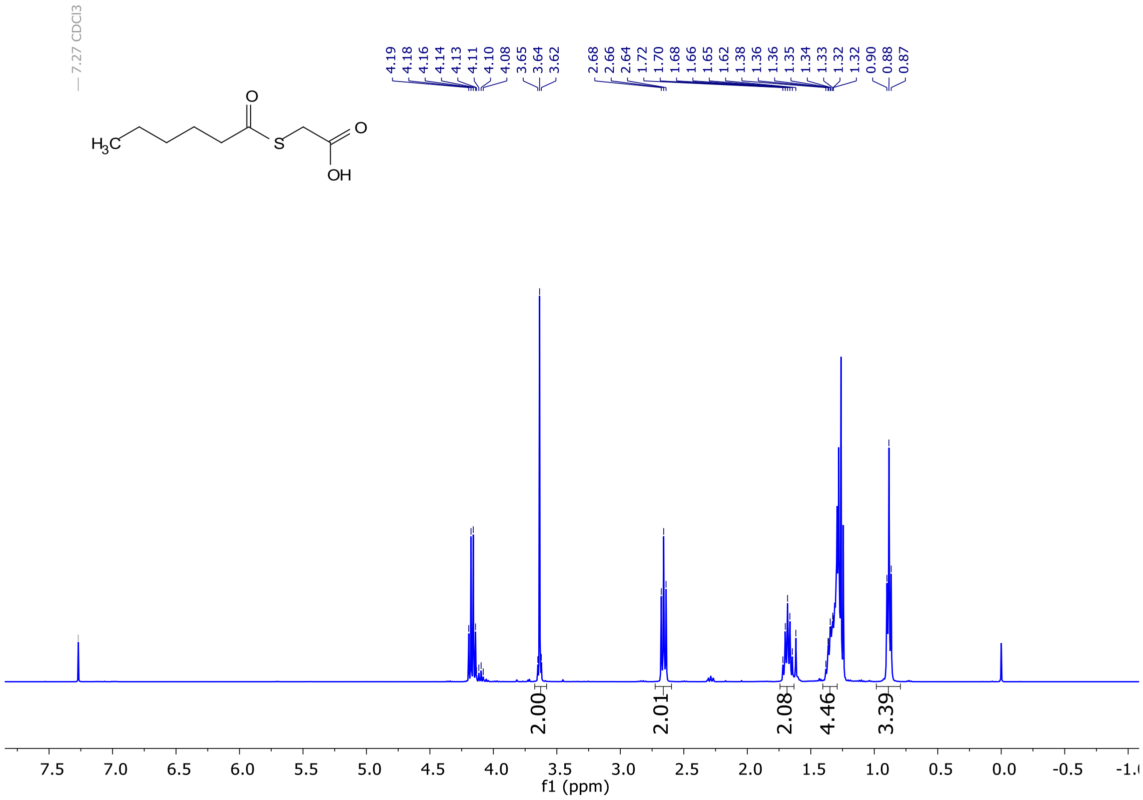

*Figure S29. ^{1}H-NMR spectrum of compound **B3**.*

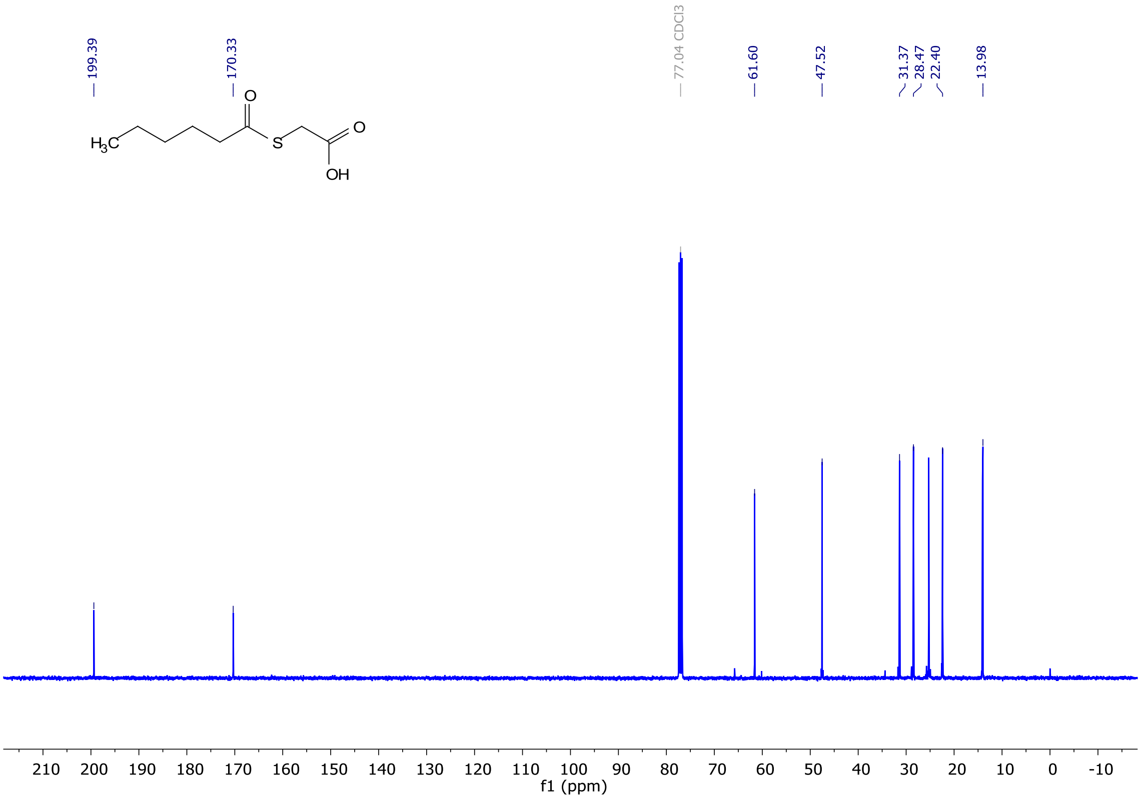

*Figure S30. ^{13}C-NMR spectrum of compound **B3**.*

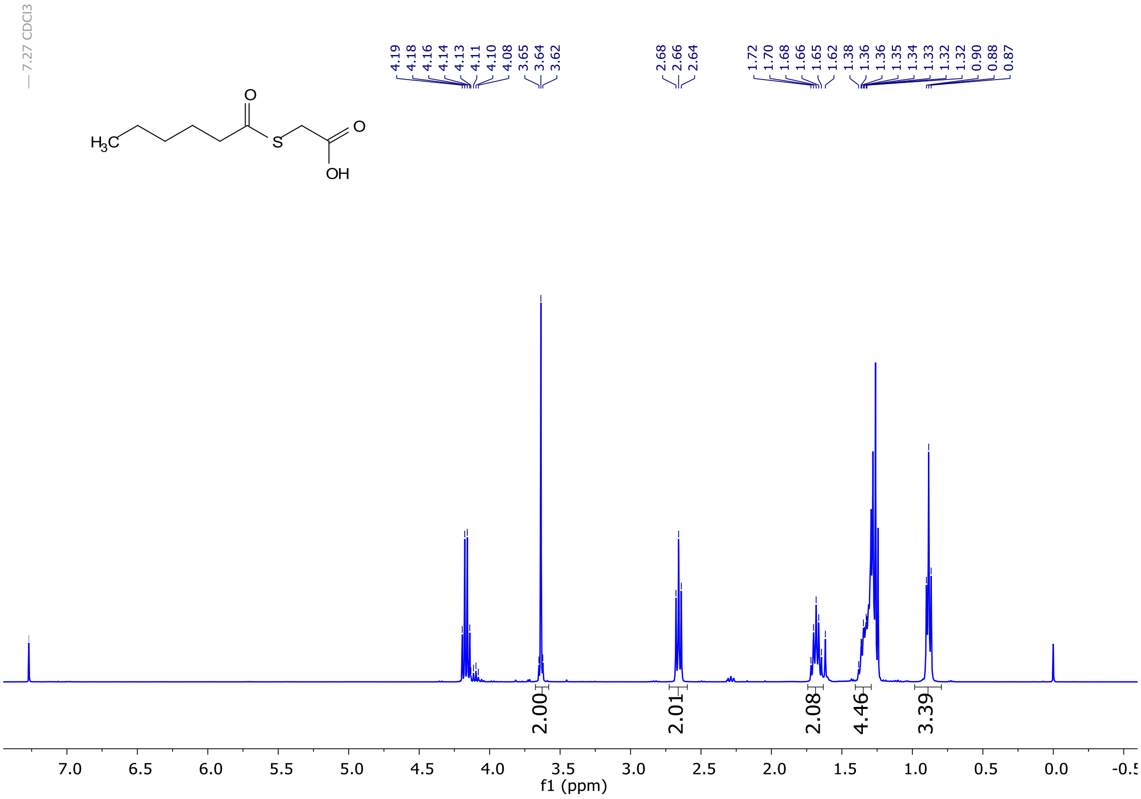

*Figure S31. ^{1}H-NMR spectrum of compound **B4.***

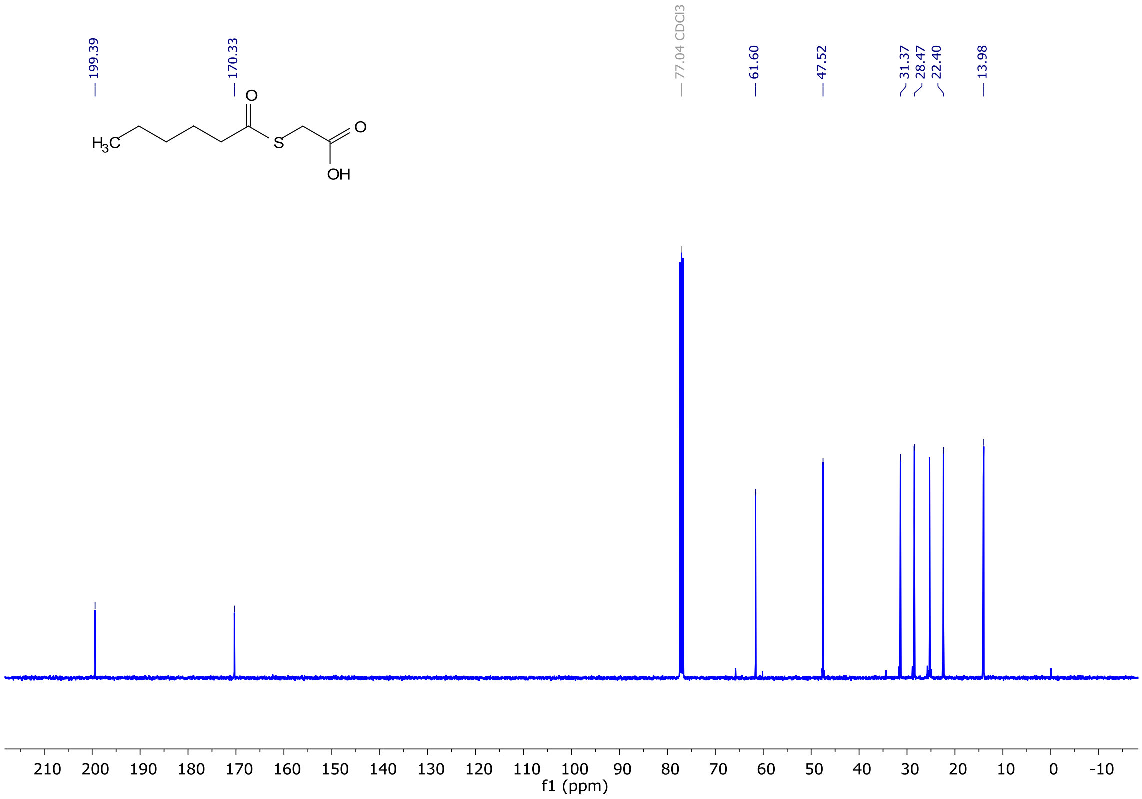

*Figure S32. 13C-NMR spectrum of compound **B4**.*

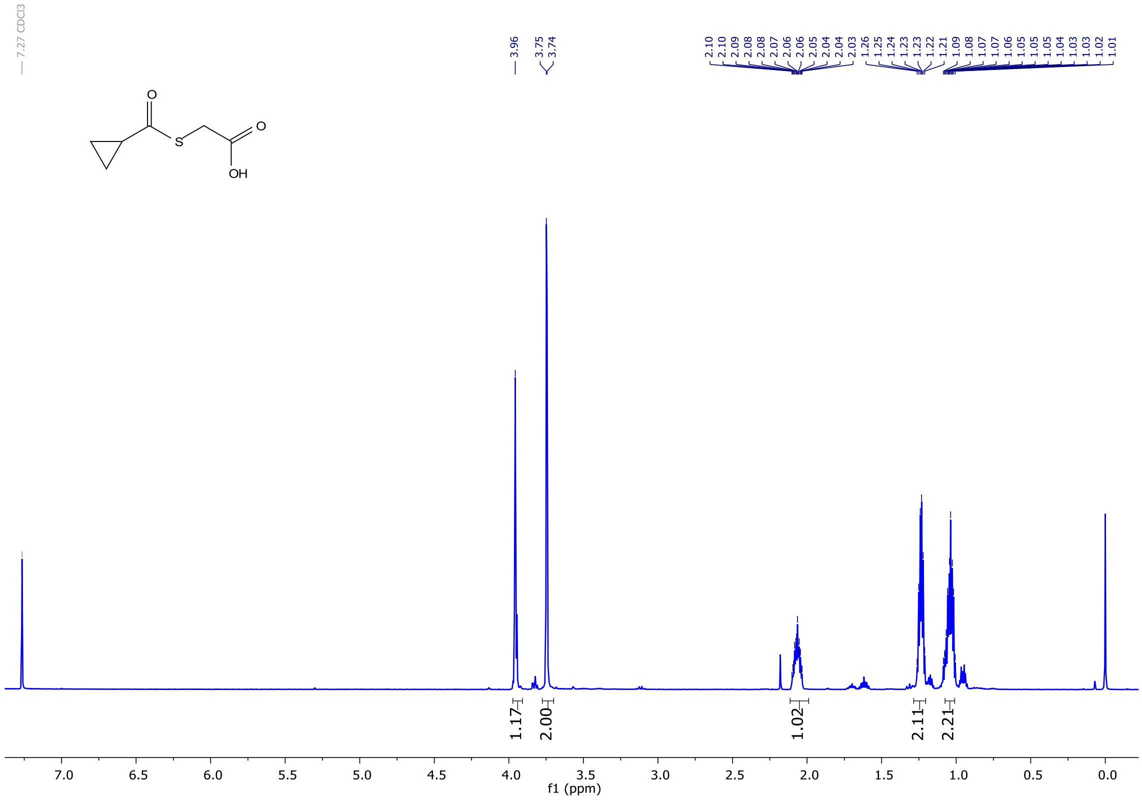

*Figure S33. 1H-NMR spectrum of compound **B5.***

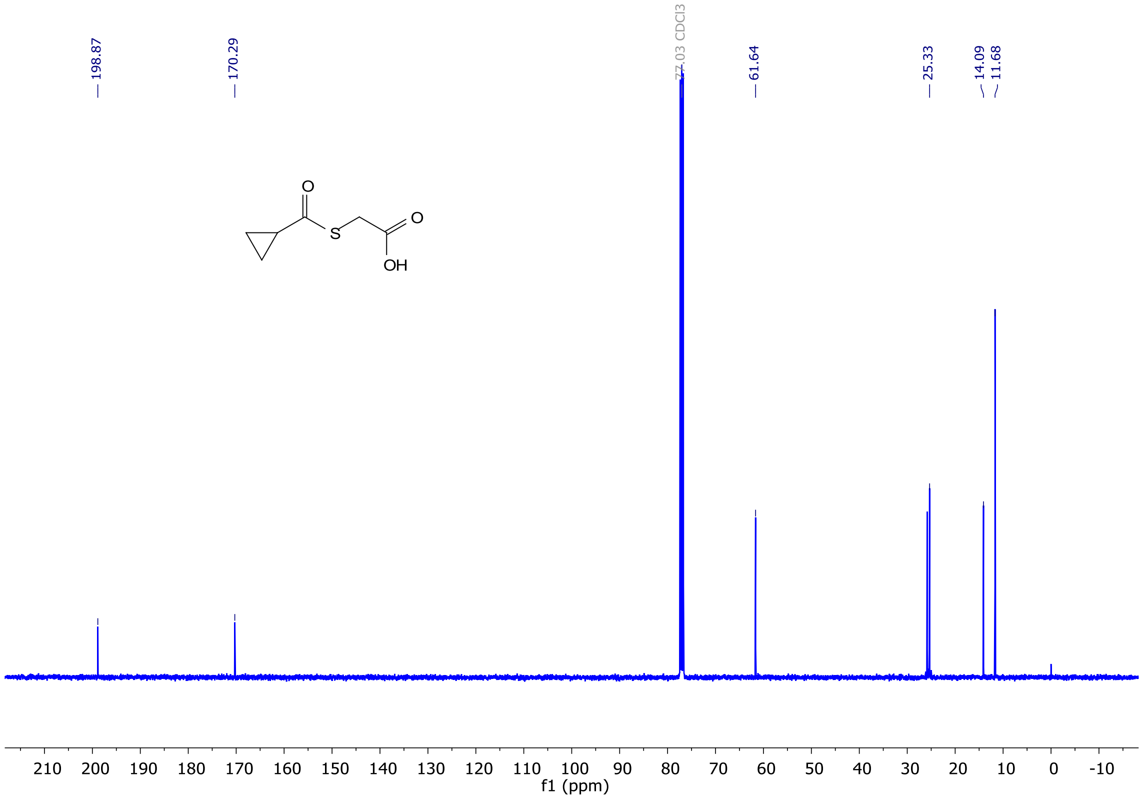

Figure S34. ^{13}C-NMR spectrum of compound ***B5****.*

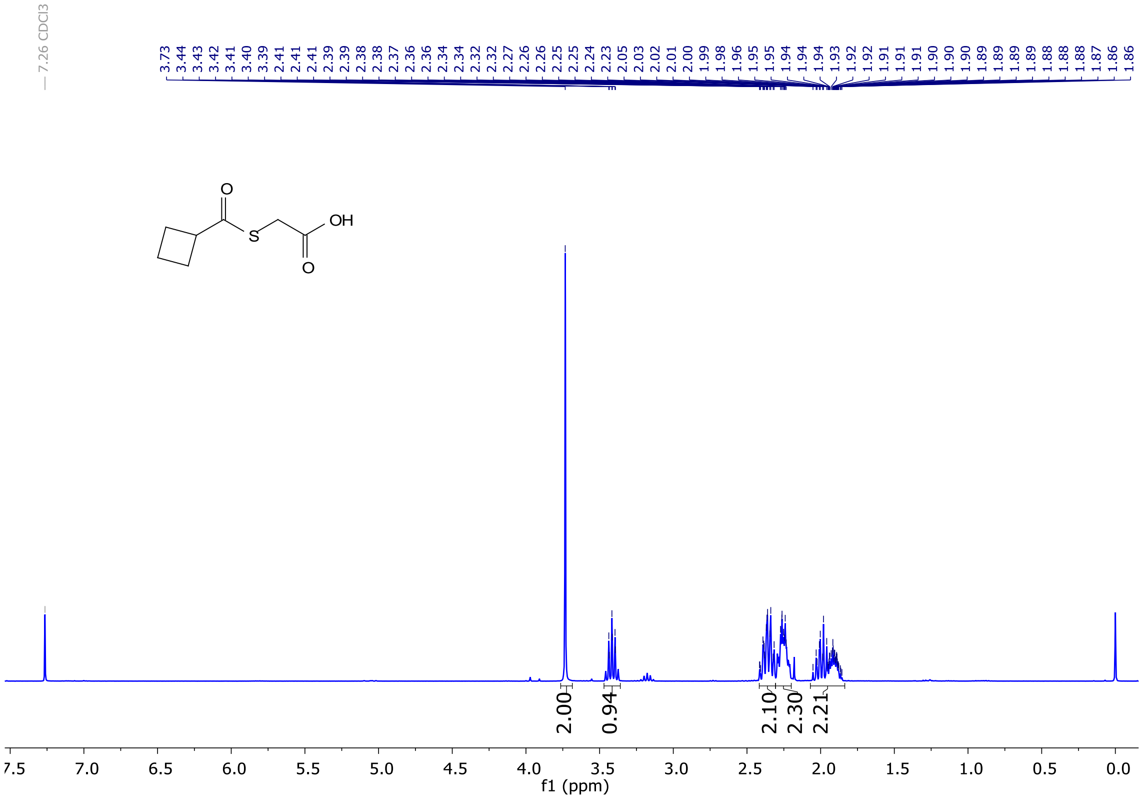

Figure S35. ^{1}H-NMR spectrum of compound ***B6.***

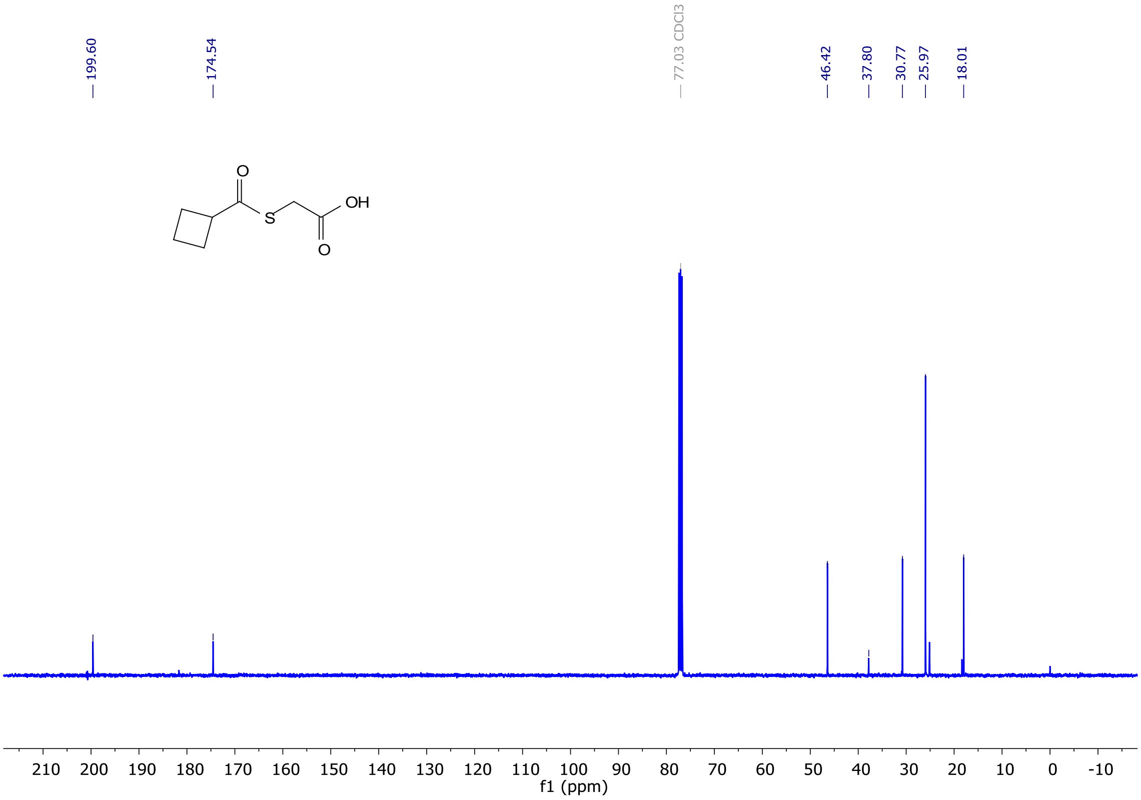

*Figure S36. ^{13}C-NMR spectrum of compound **B6**.*

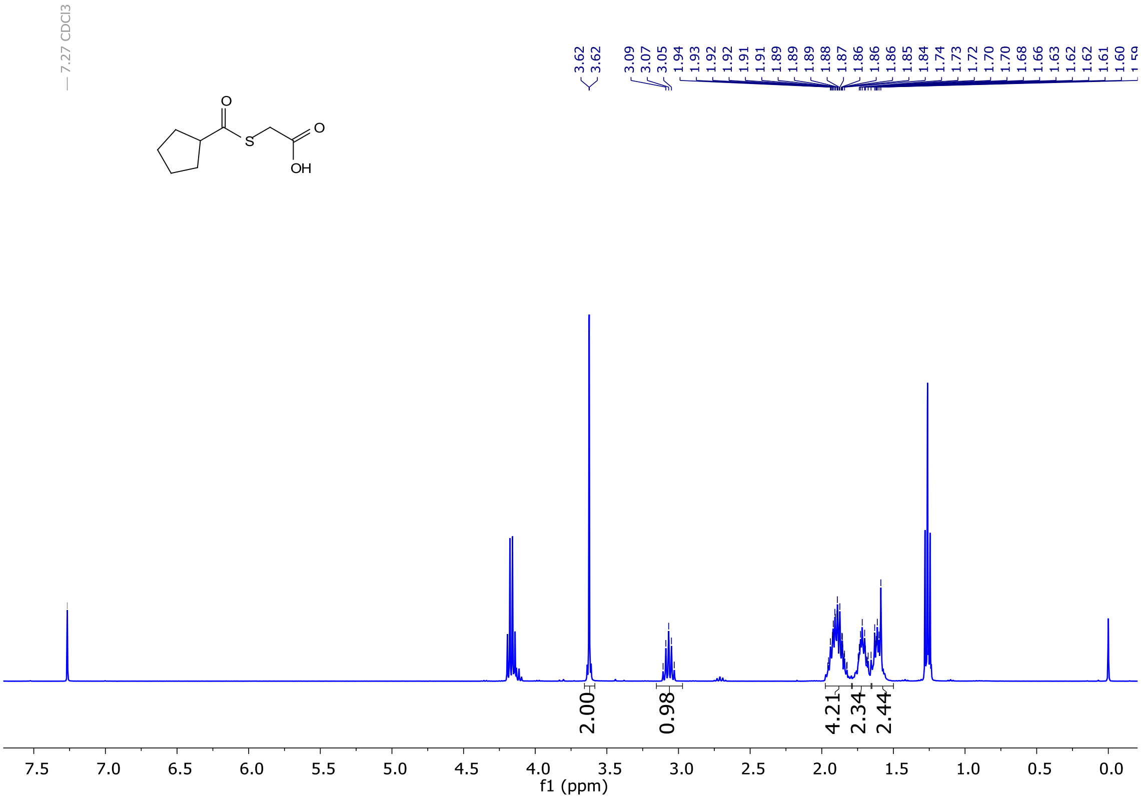

*Figure S37. ^{1}H-NMR spectrum of compound **B7.***

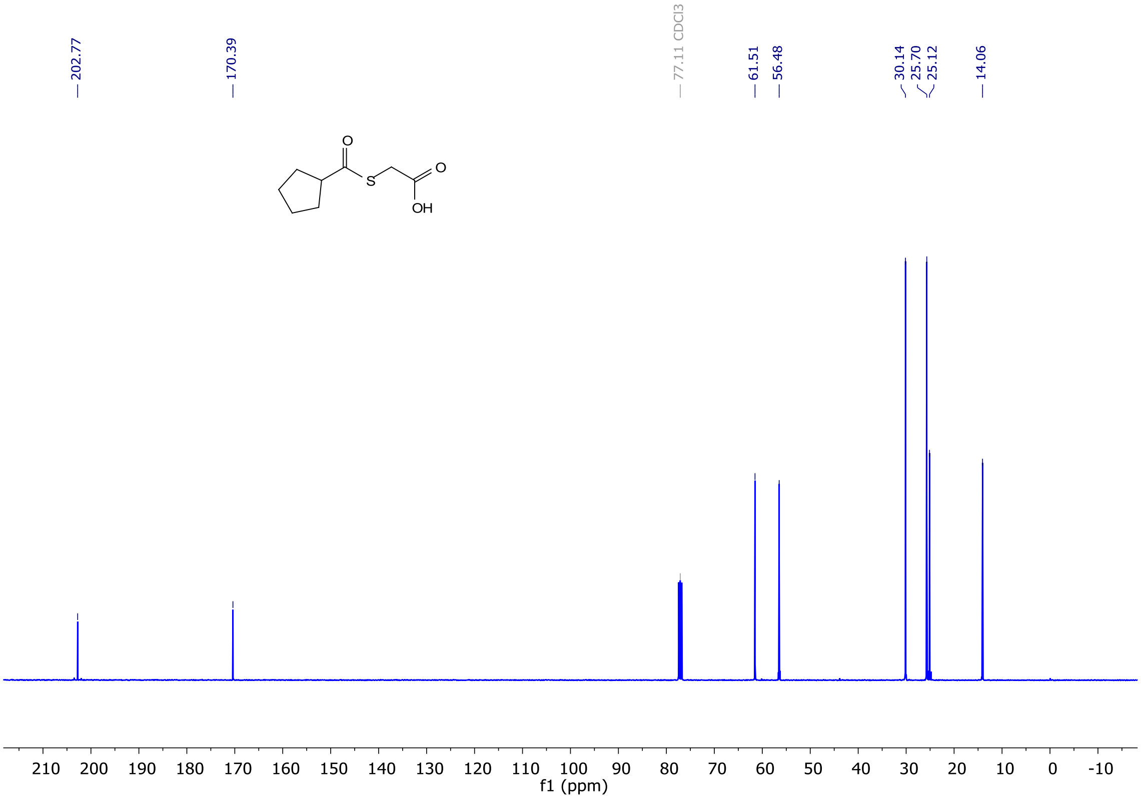

*Figure S38. ^{13}C-NMR spectrum of compound **B7**.*

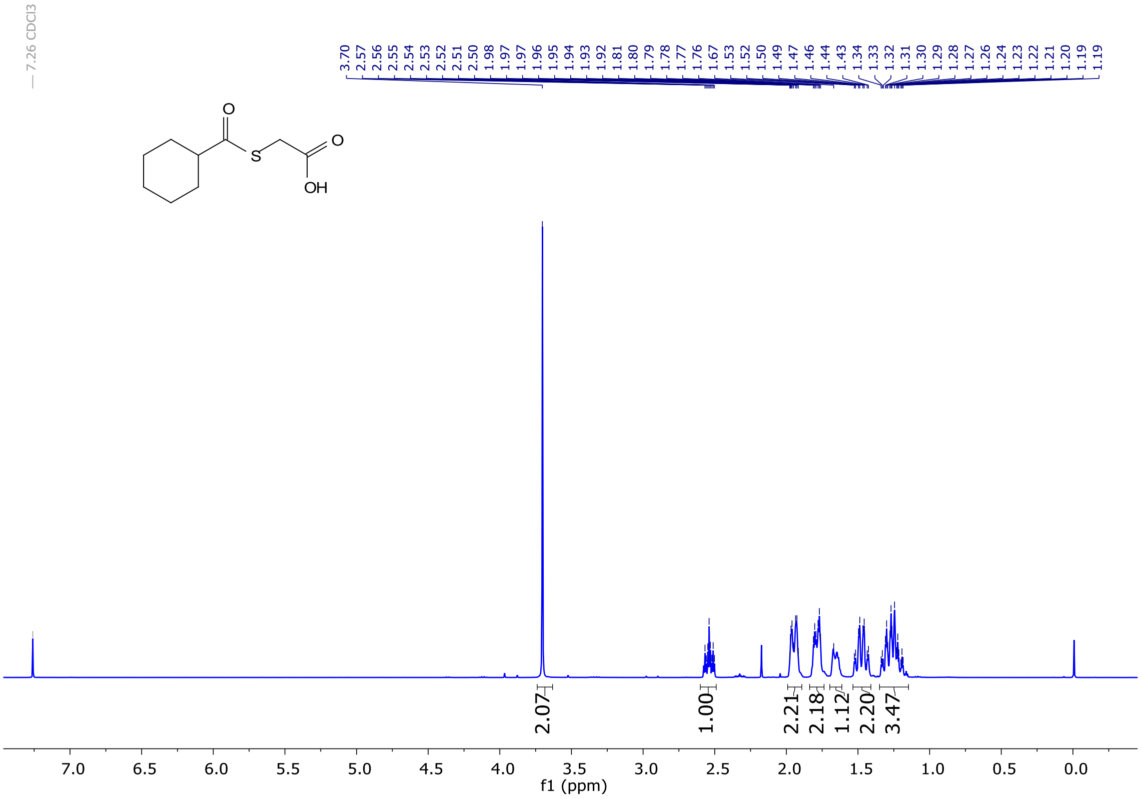

*Figure S39. ^{1}H-NMR spectrum of compound **B8.***

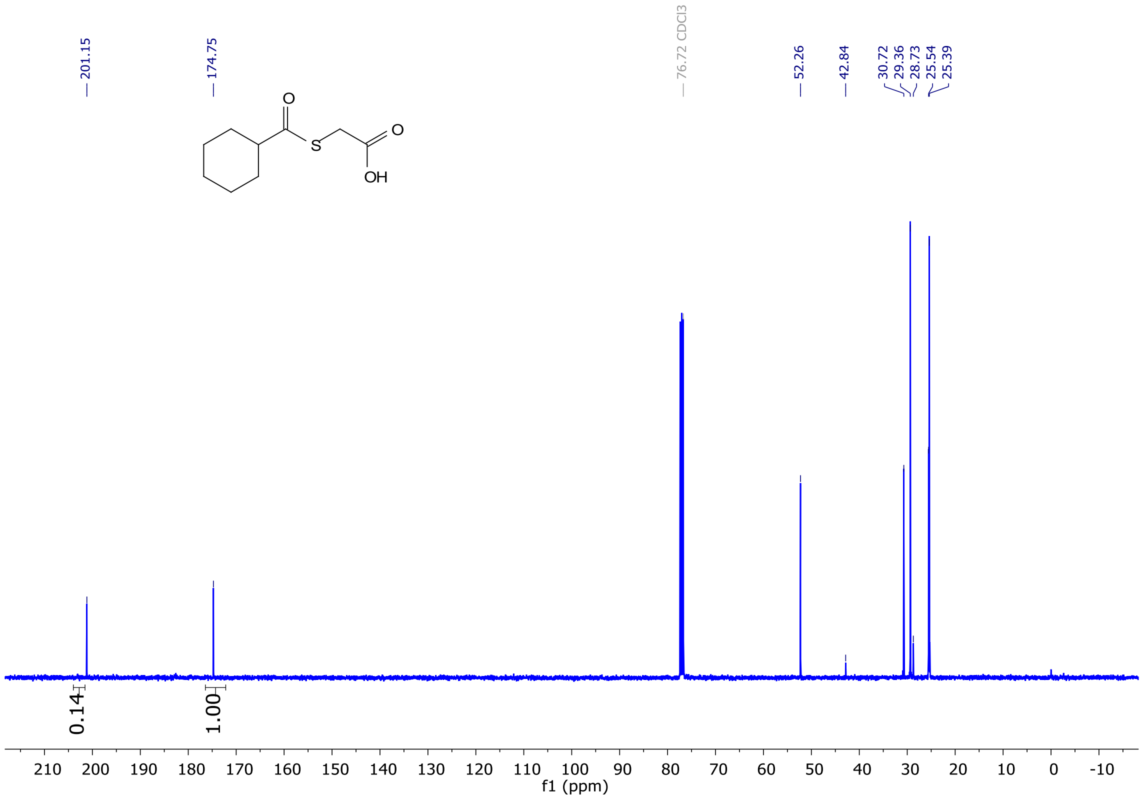

*Figure S40. ^{13}C-NMR spectrum of compound **B8**.*

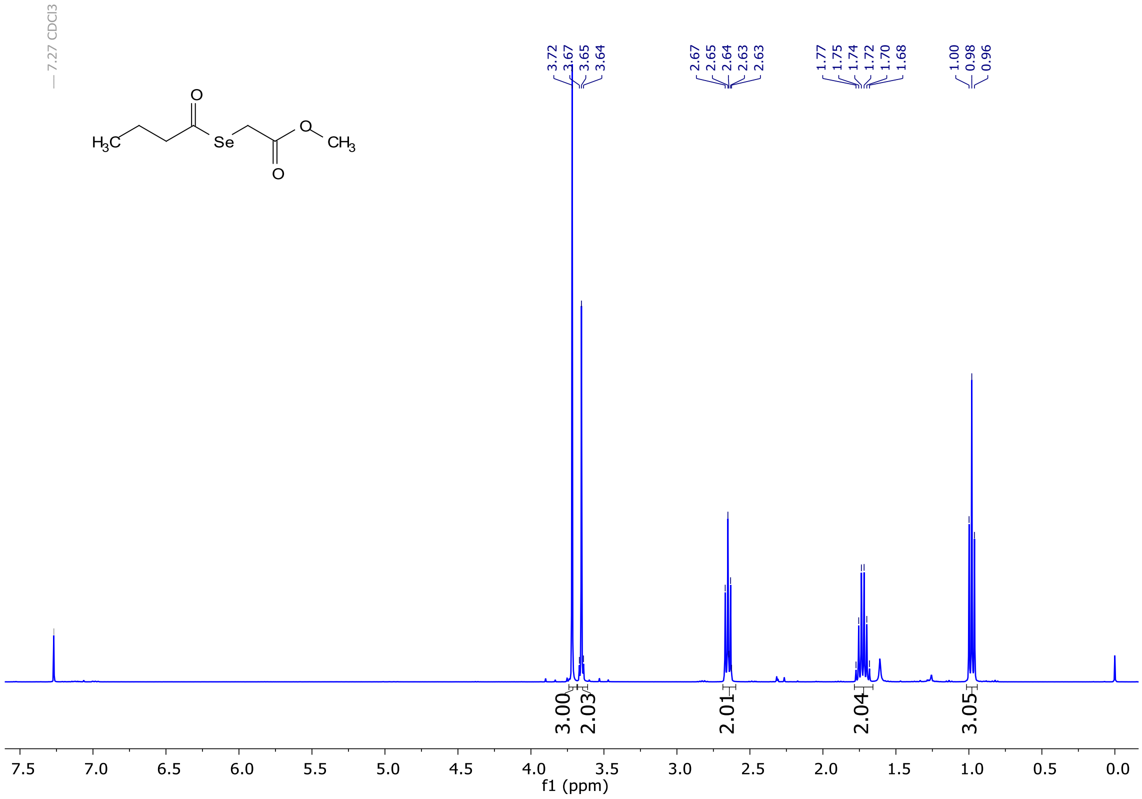

*Figure S41. ^{1}H-NMR spectrum of compound **C1.***

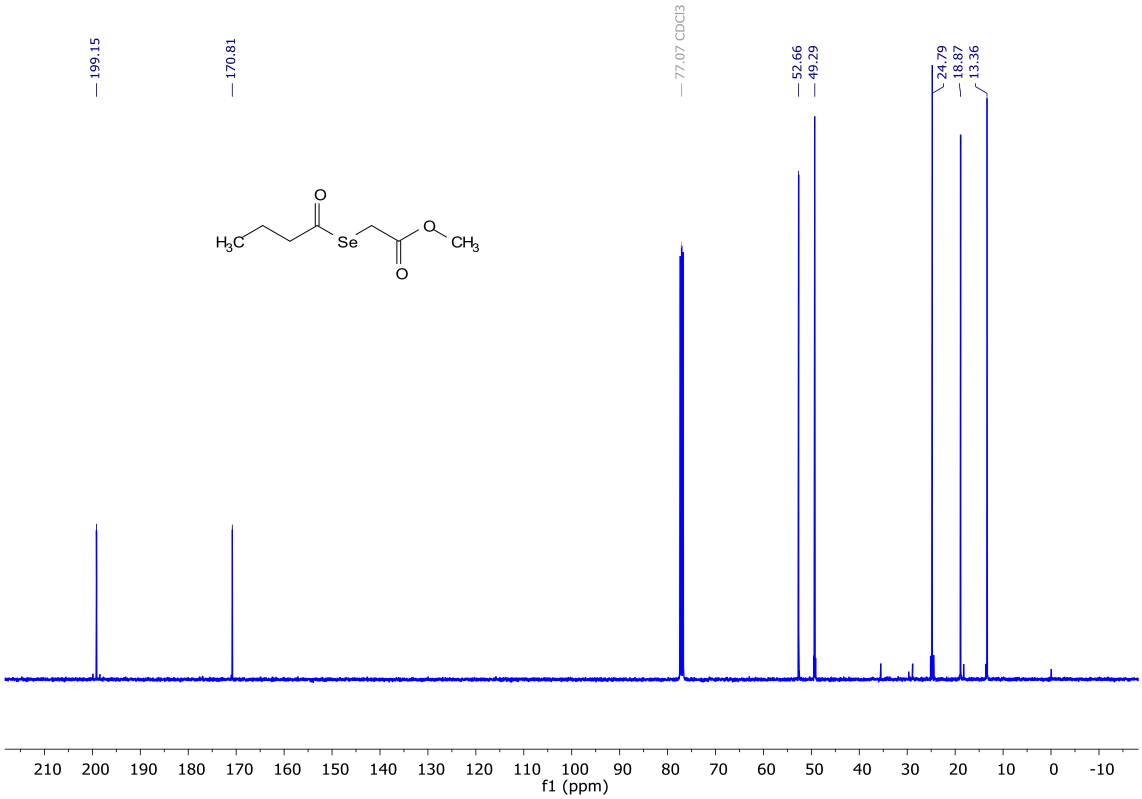

*Figure S42. ^{13}C-NMR spectrum of compound **C1**.*

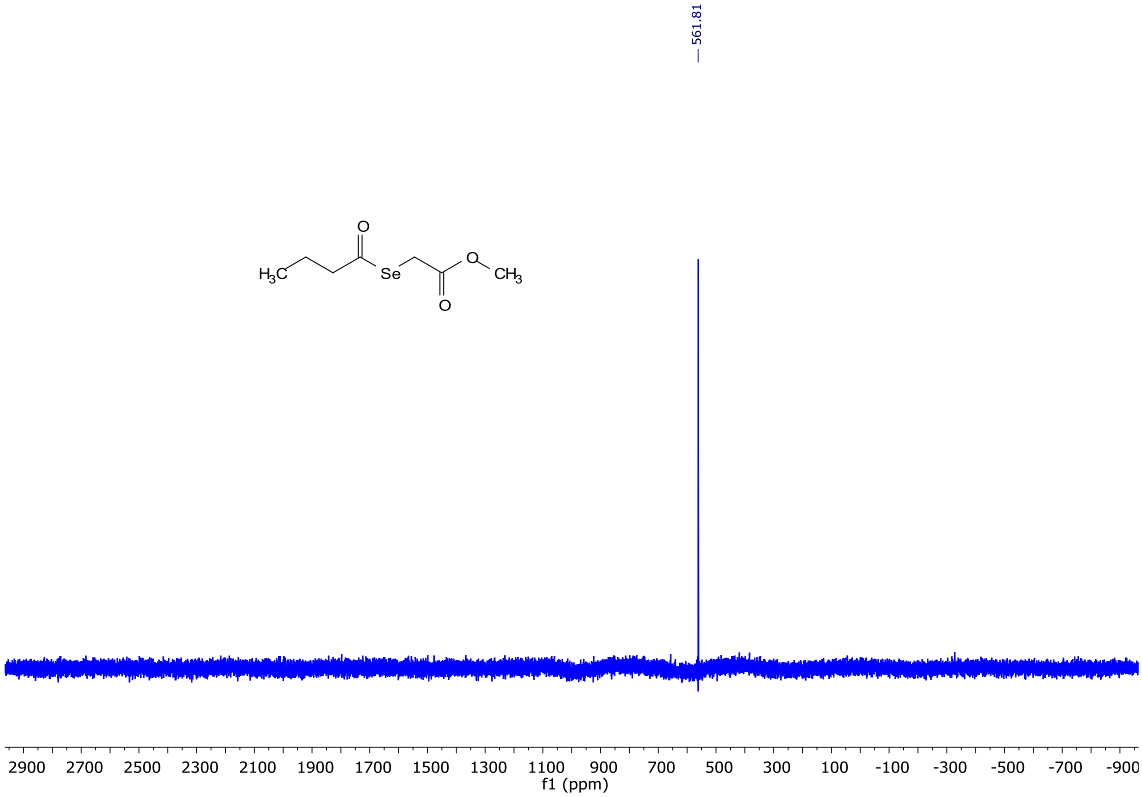

*Figure S43. ^{77}Se-NMR spectrum of compound **C1.***

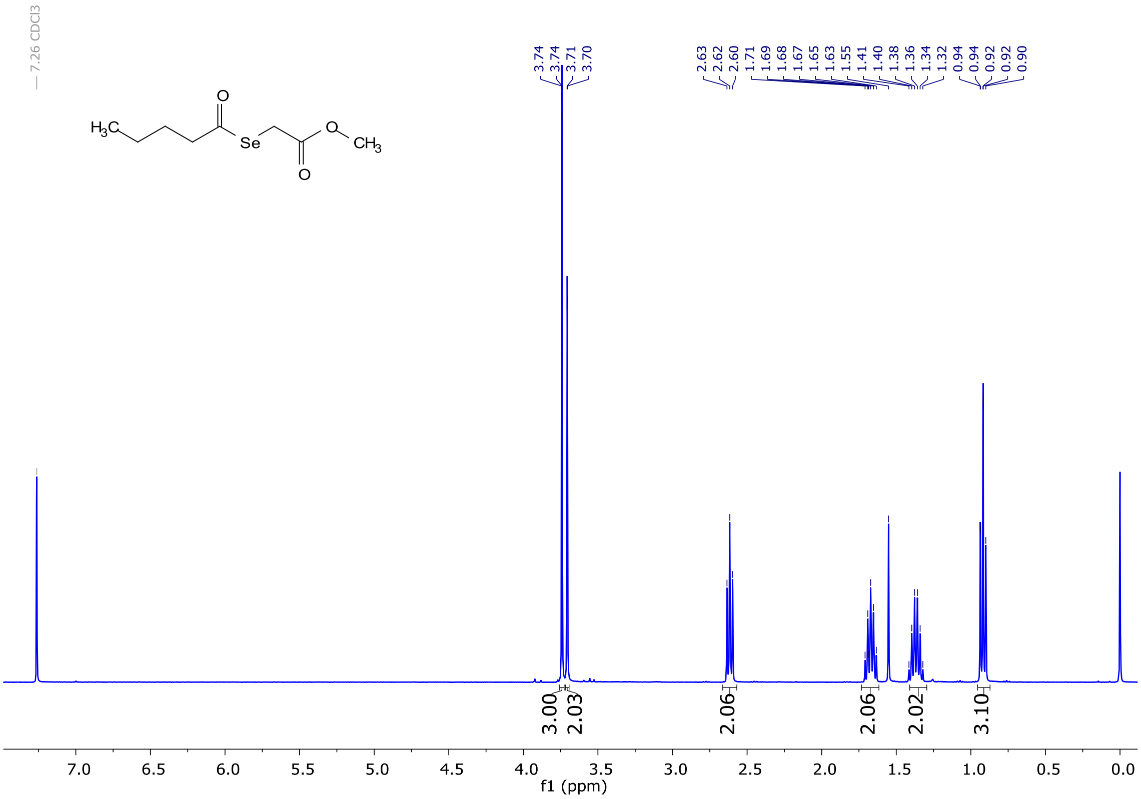

*Figure S44. ^{1}H-NMR spectrum of compound **C2.***

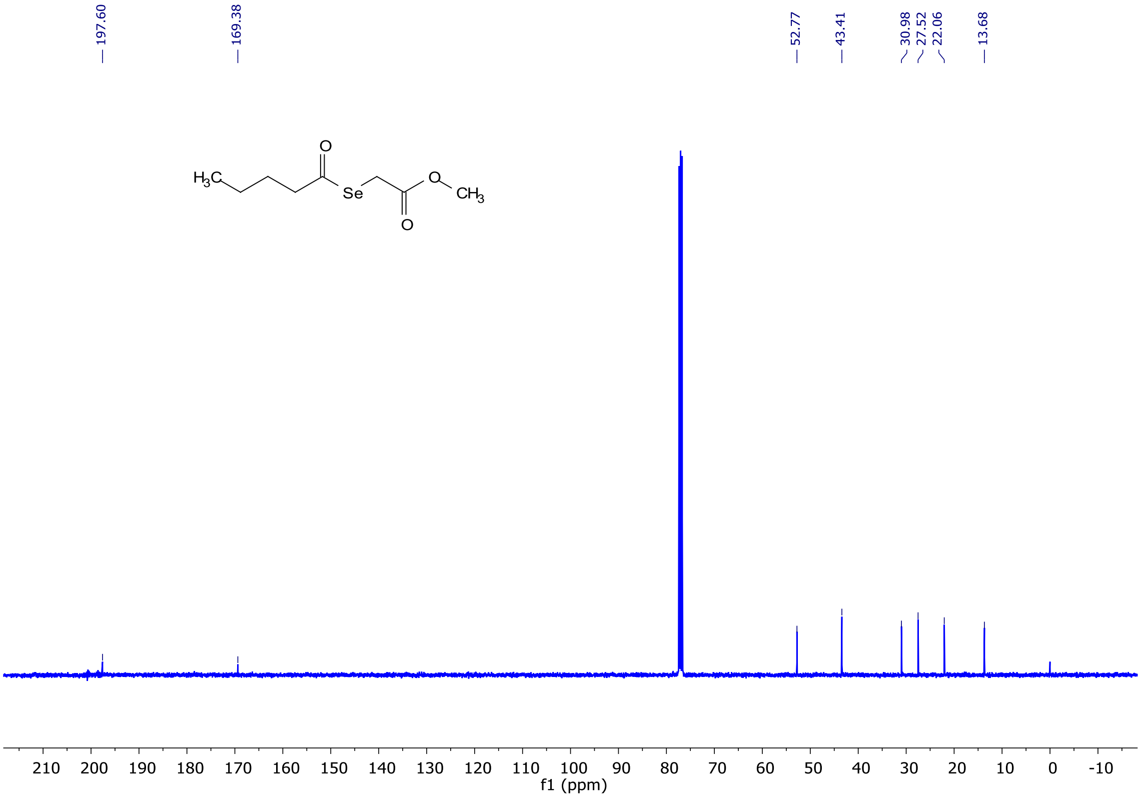

*Figure S45. ^{13}C-NMR spectrum of compound **C2**.*

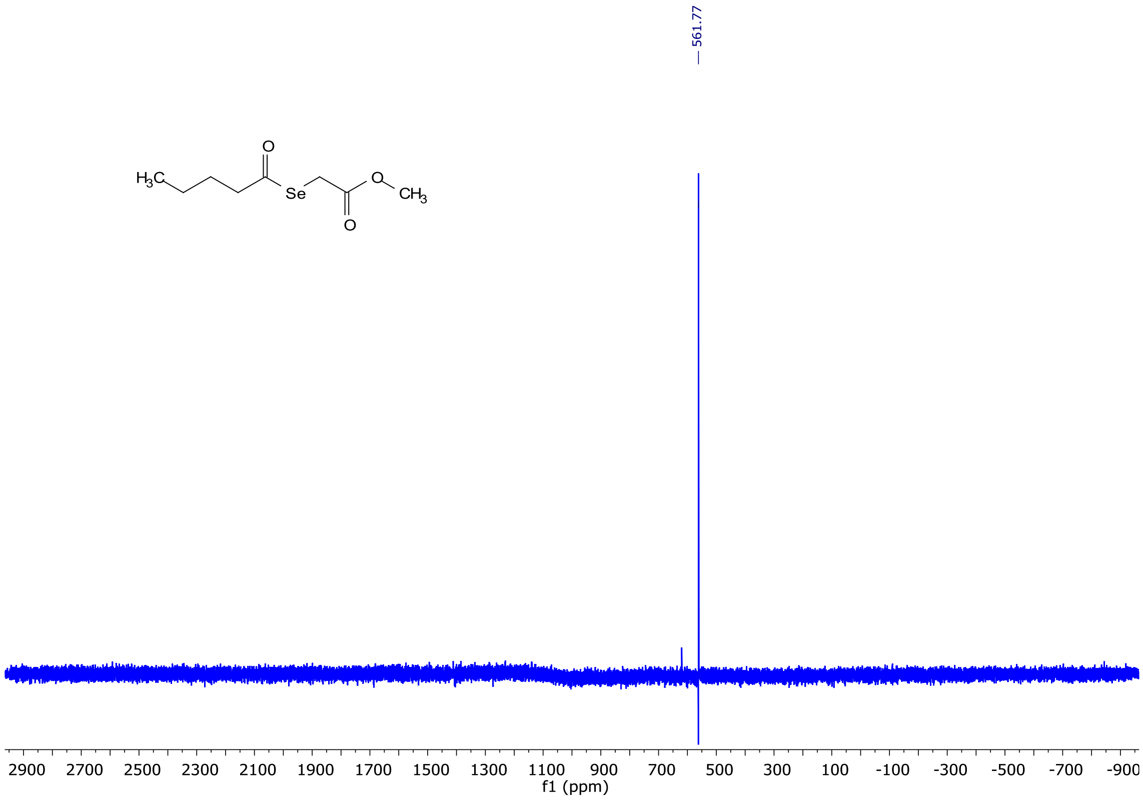

Figure S46. ^{77}*Se-NMR spectrum of compound* ***C2.***

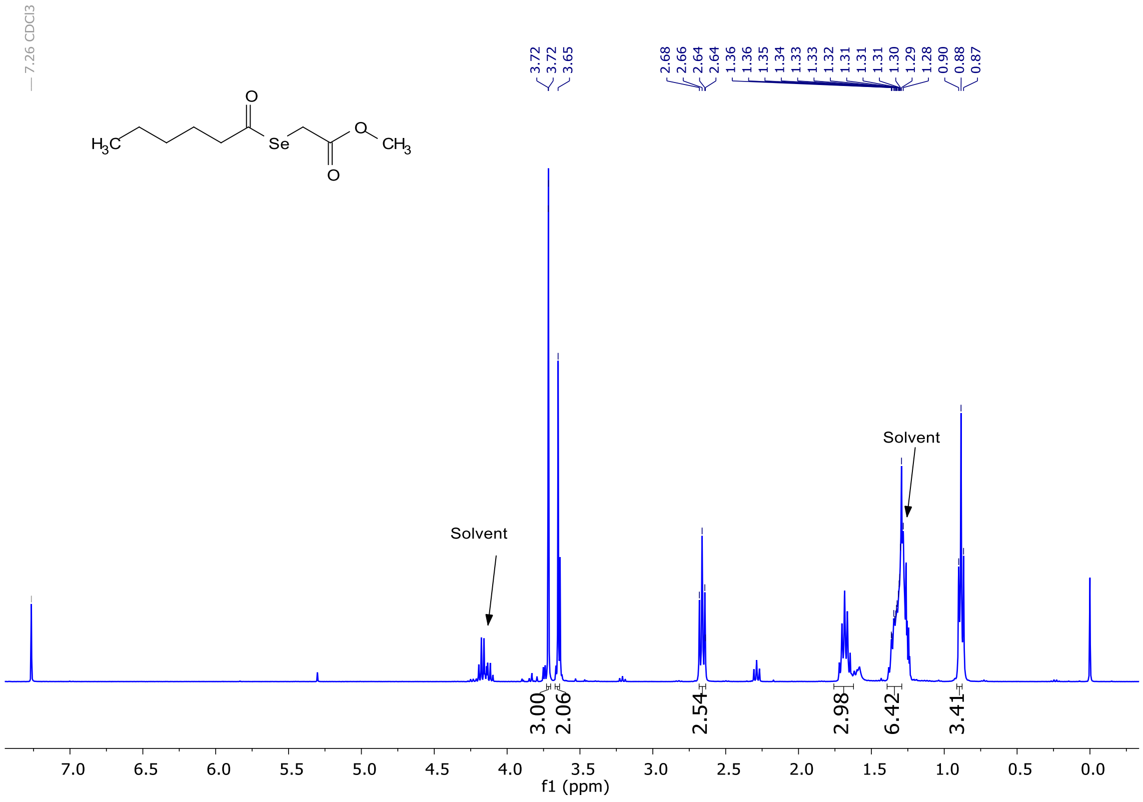

Figure S47. ^{1}*H-NMR spectrum of compound* ***C3.***

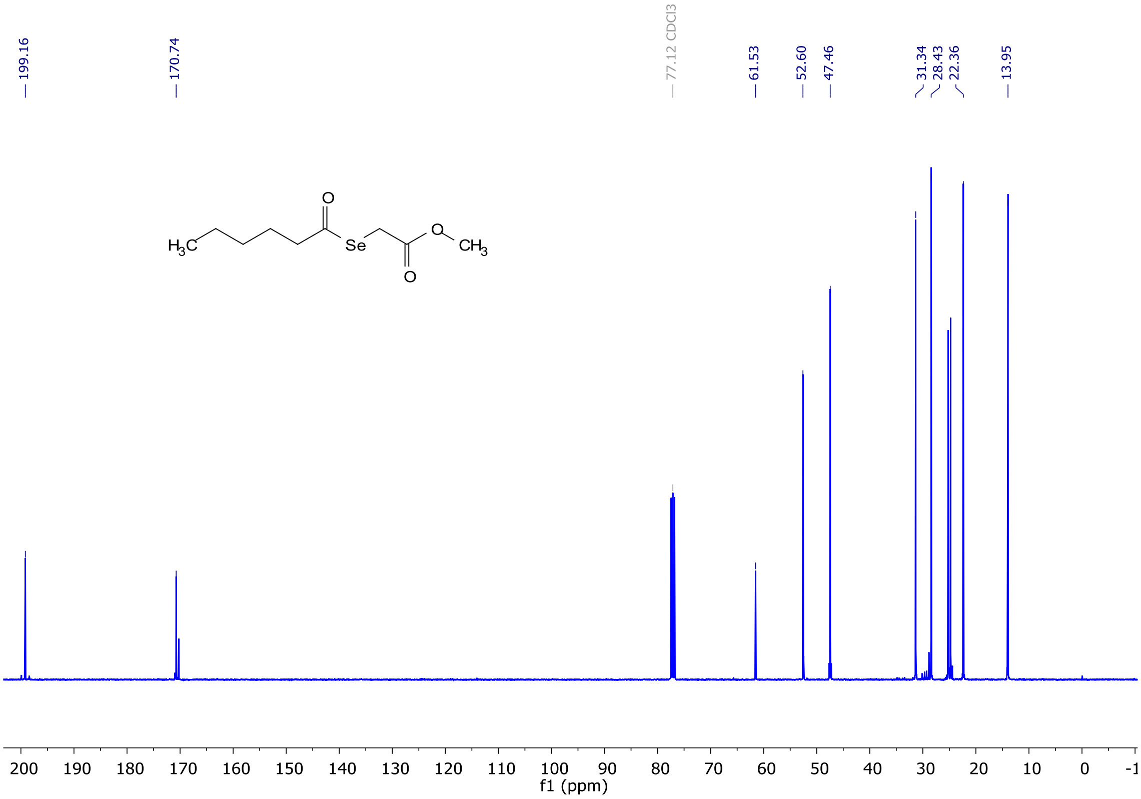

*Figure S48. ^{13}C-NMR spectrum of compound **C3**.*

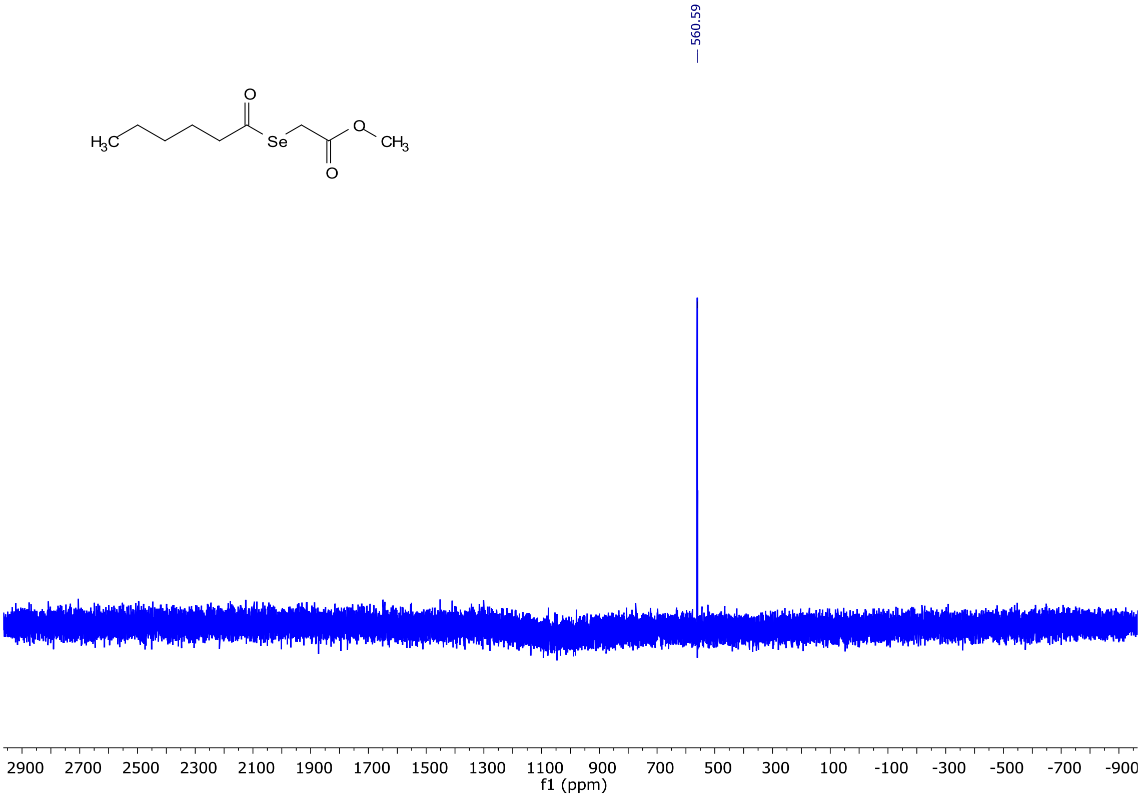

*Figure S49. ^{77}Se-NMR spectrum of compound **C3.***

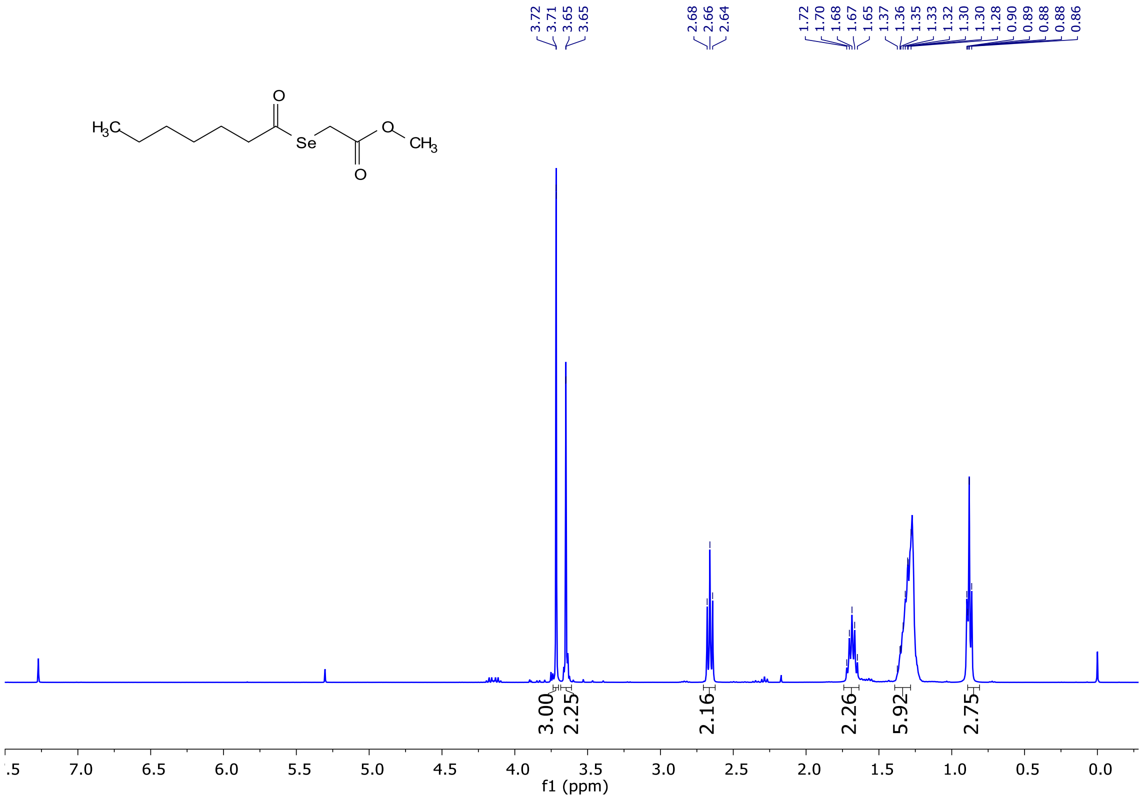

Figure S50. ^{1}H-NMR spectrum of compound **C4.**

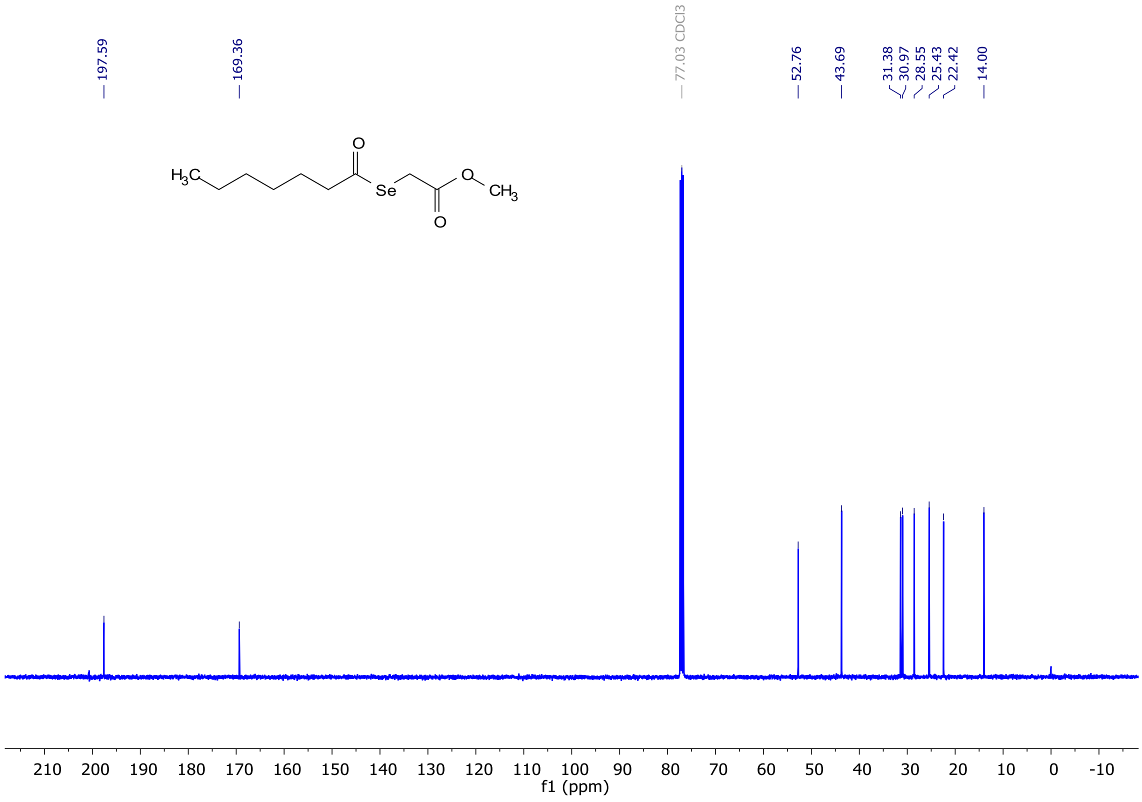

Figure S51. ^{13}C-NMR spectrum of compound **C4**.

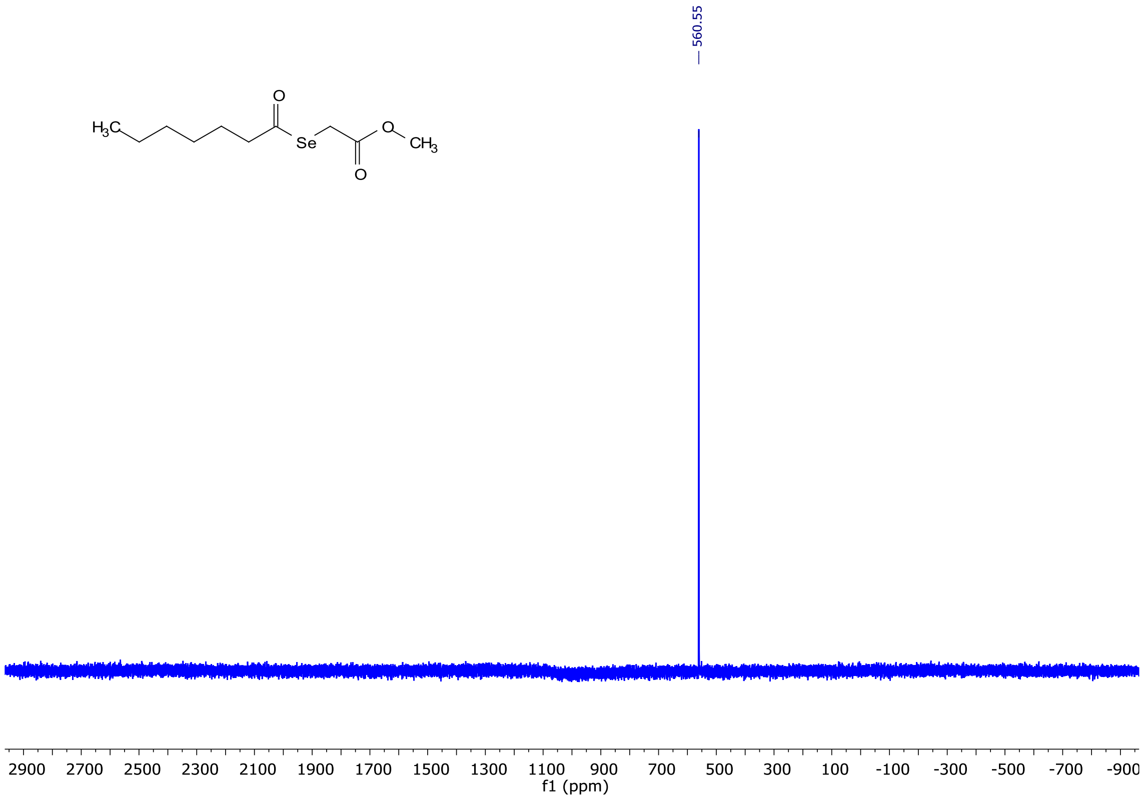

*Figure S52. ^{77}Se-NMR spectrum of compound **C4.***

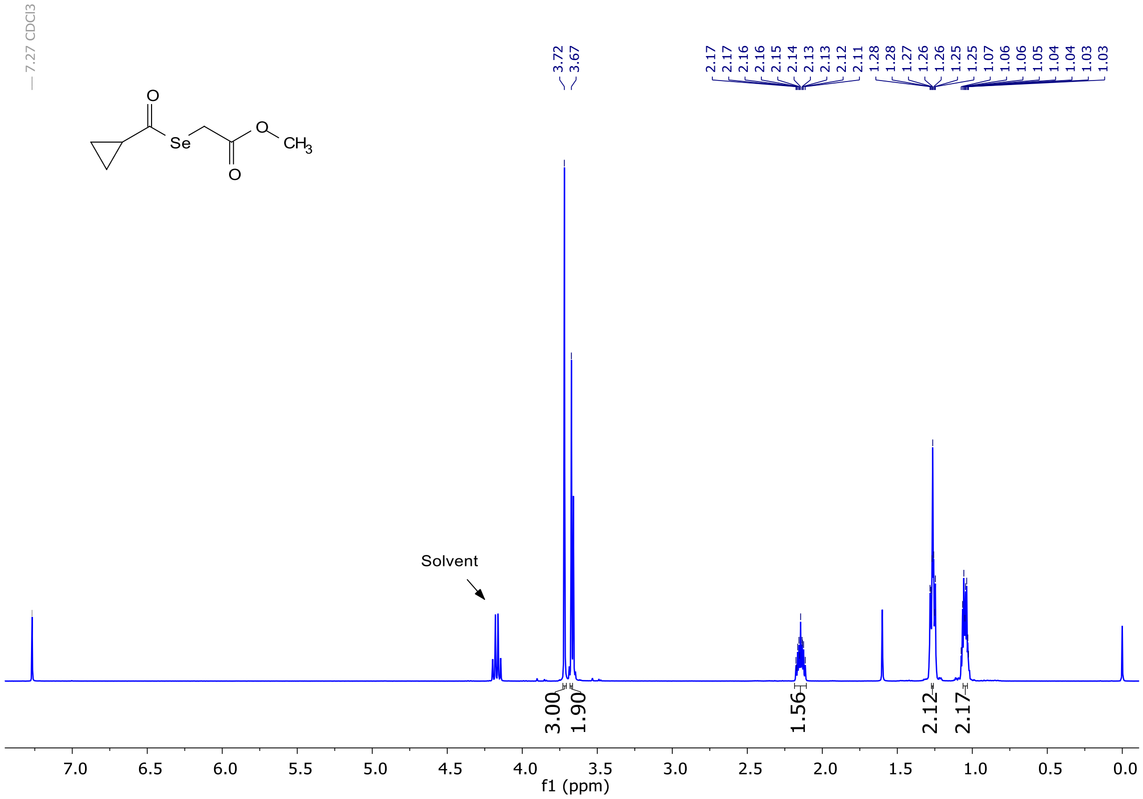

*Figure S53. ^{1}H-NMR spectrum of compound **C5.***

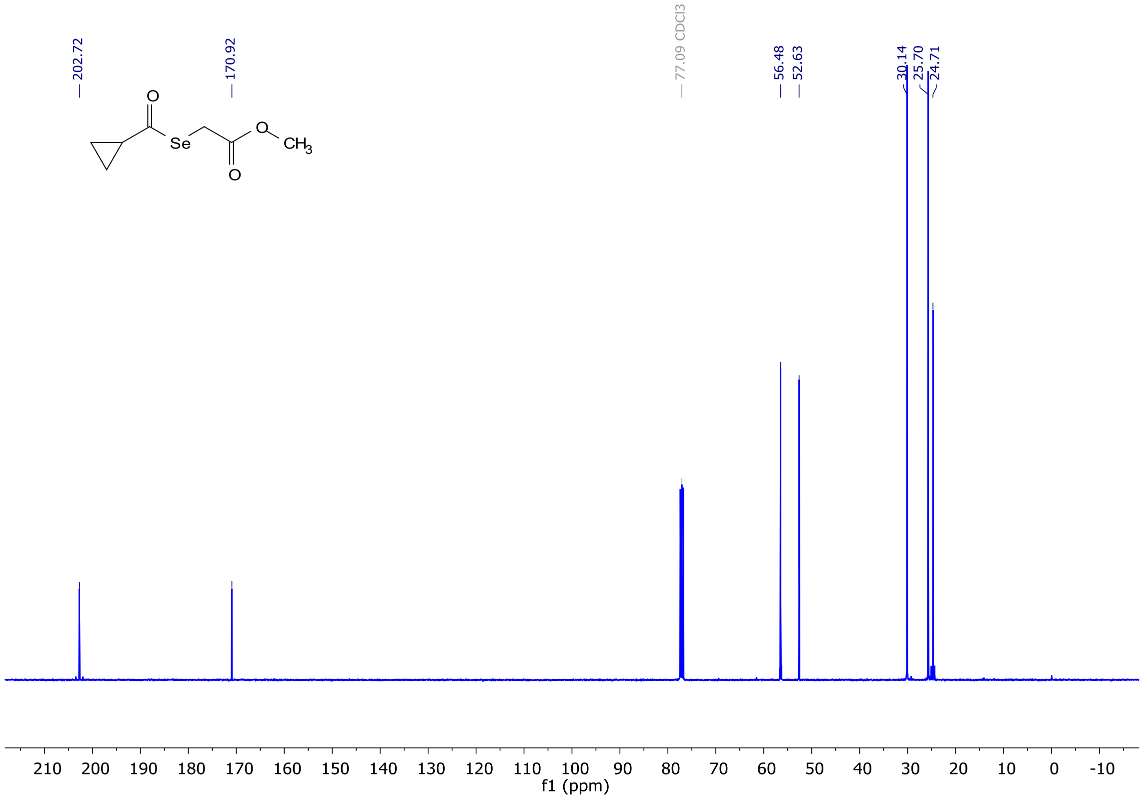

*Figure S54. ^{13}C-NMR spectrum of compound **C5**.*

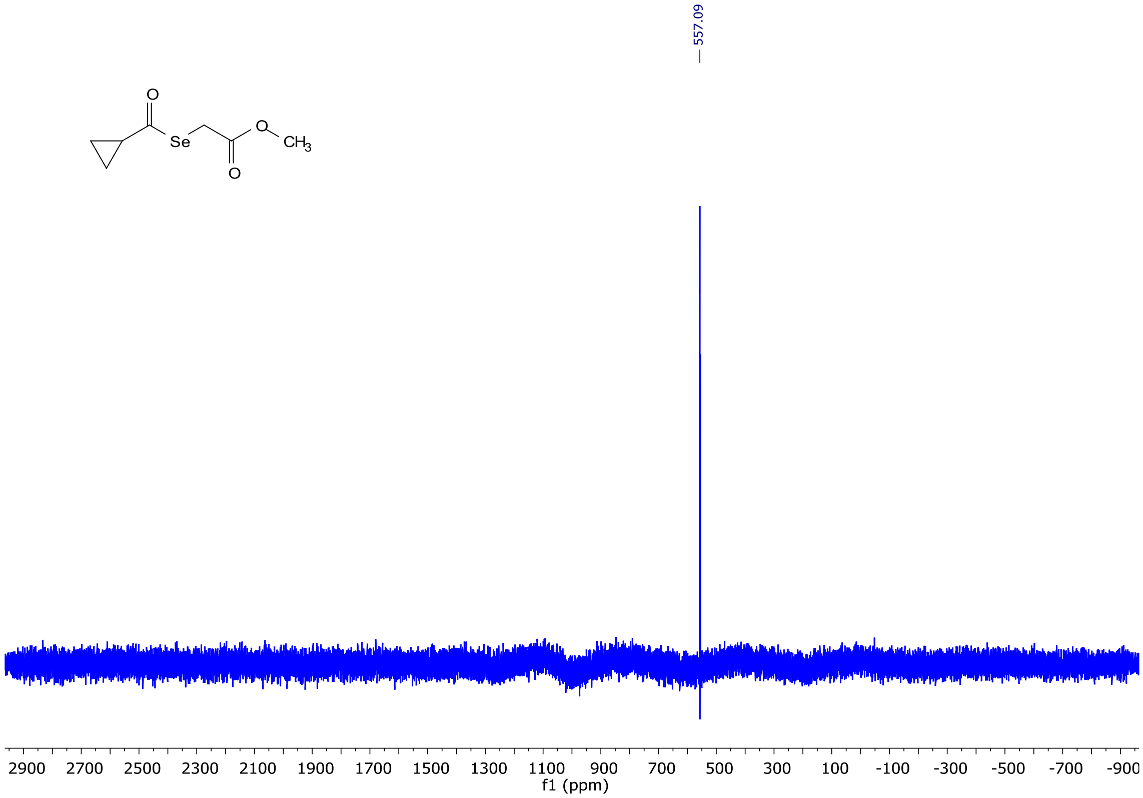

*Figure S55. ^{77}Se-NMR spectrum of compound **C5.***

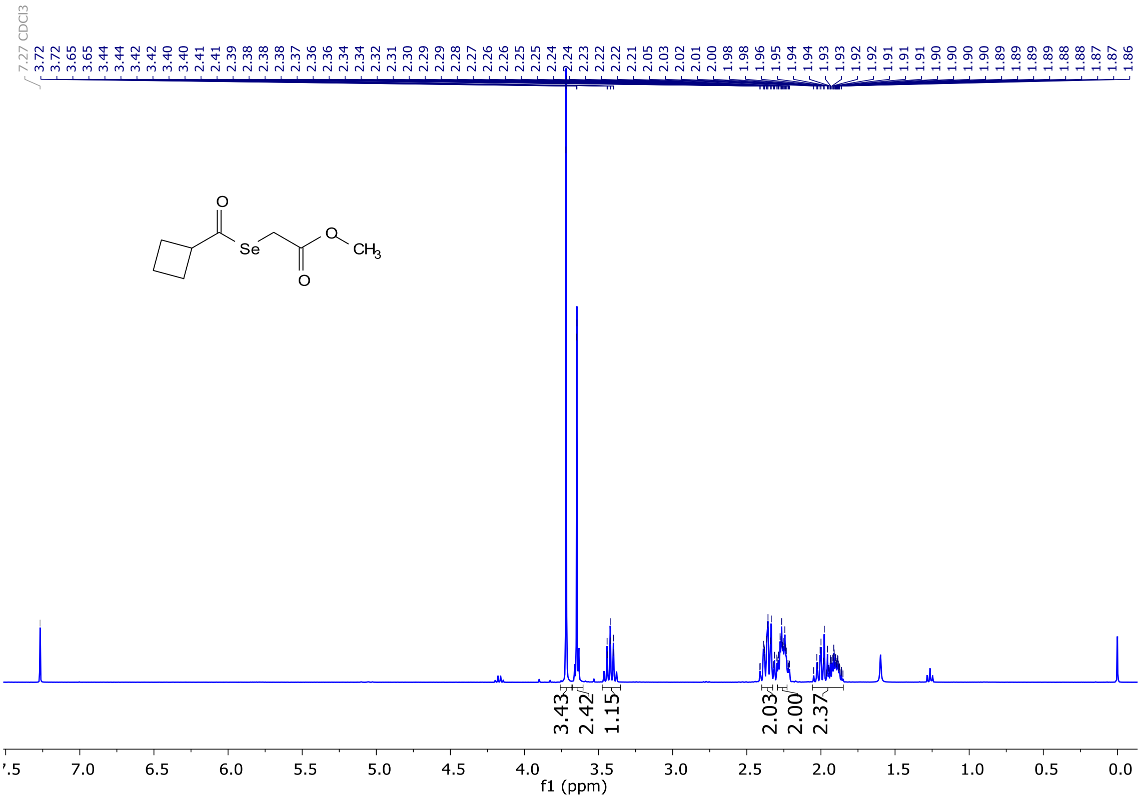

Figure S56. ^{1}H-NMR spectrum of compound ***C6.***

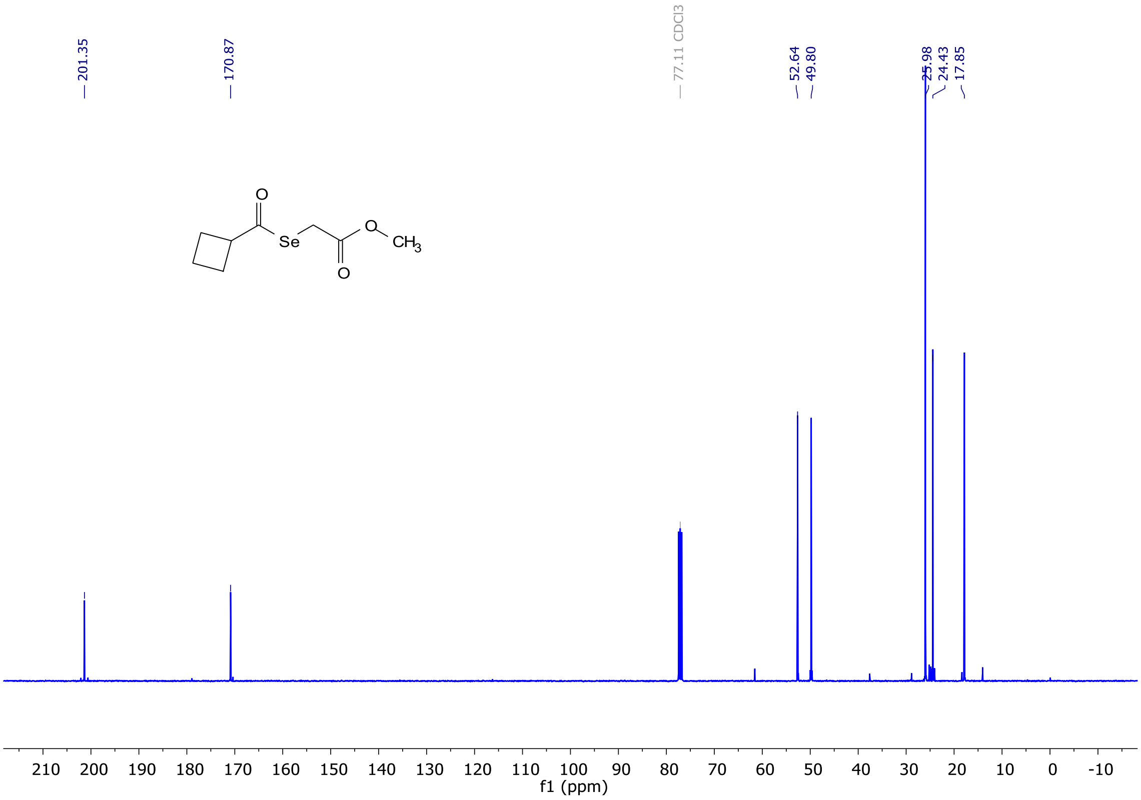

Figure S57. ^{13}C-NMR spectrum of compound ***C6****.*

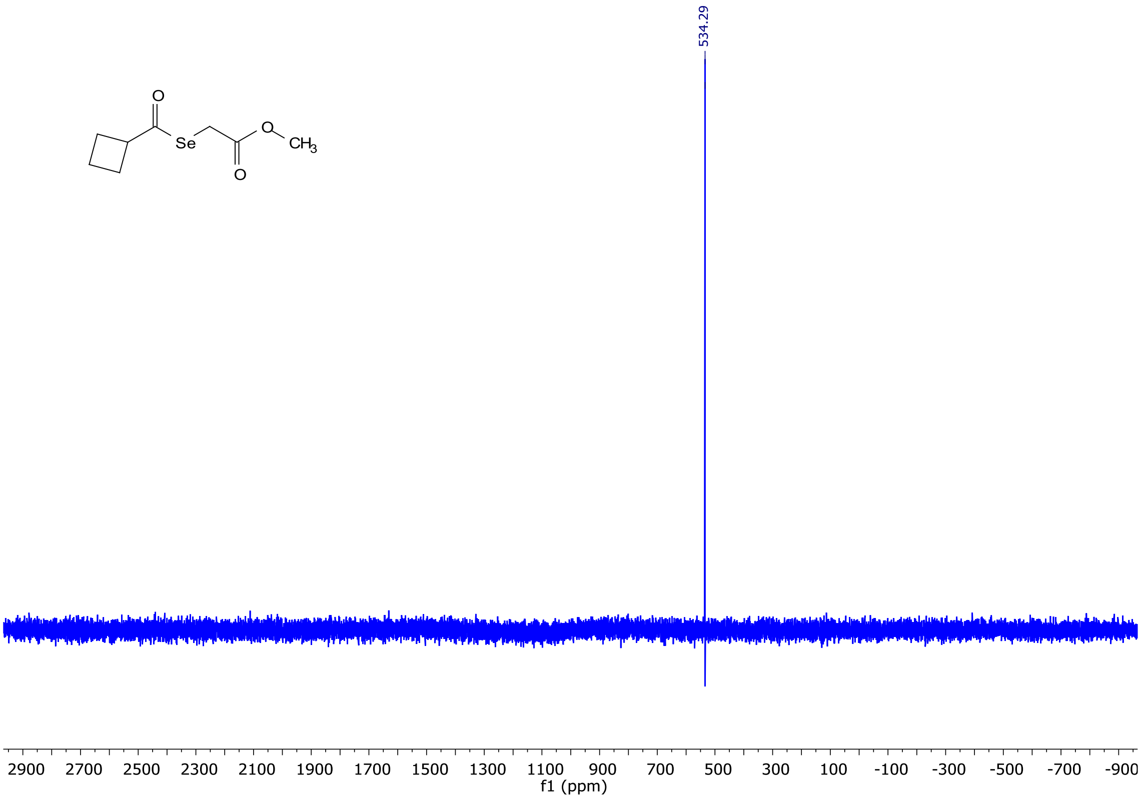

*Figure S58. ^{77}Se-NMR spectrum of compound **C6.***

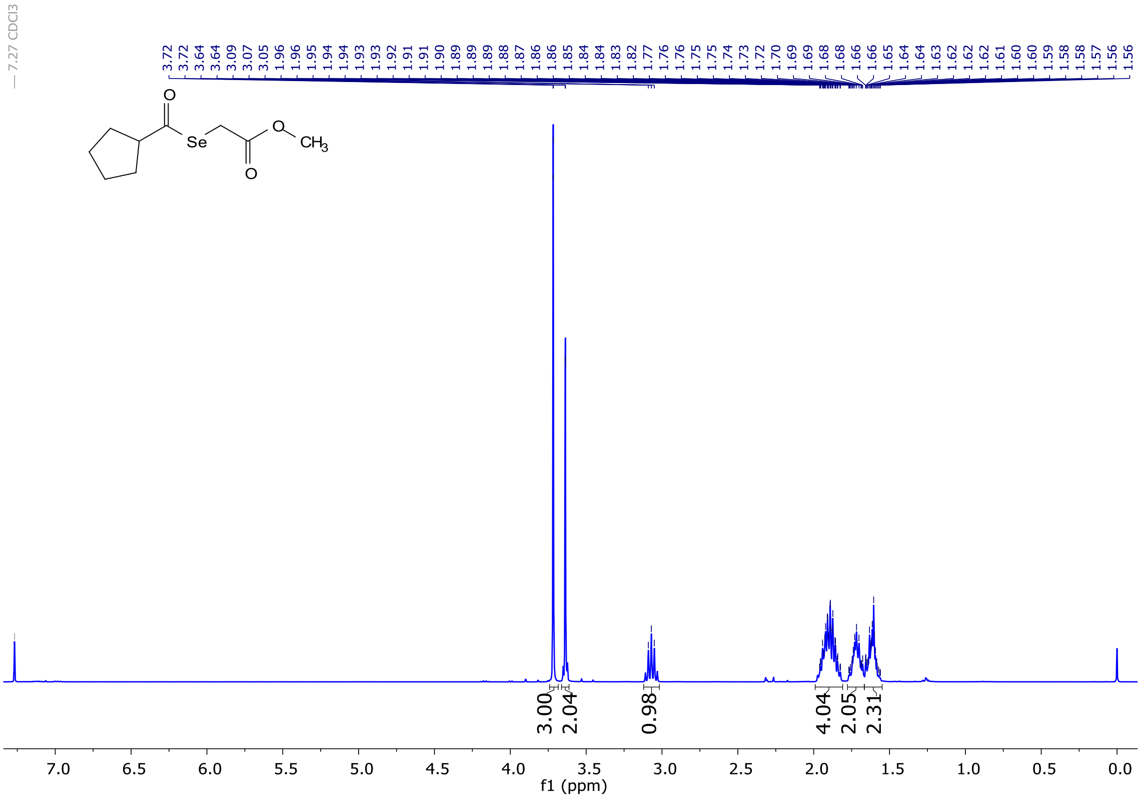

*Figure S59. ^{1}H-NMR spectrum of compound **C7.***

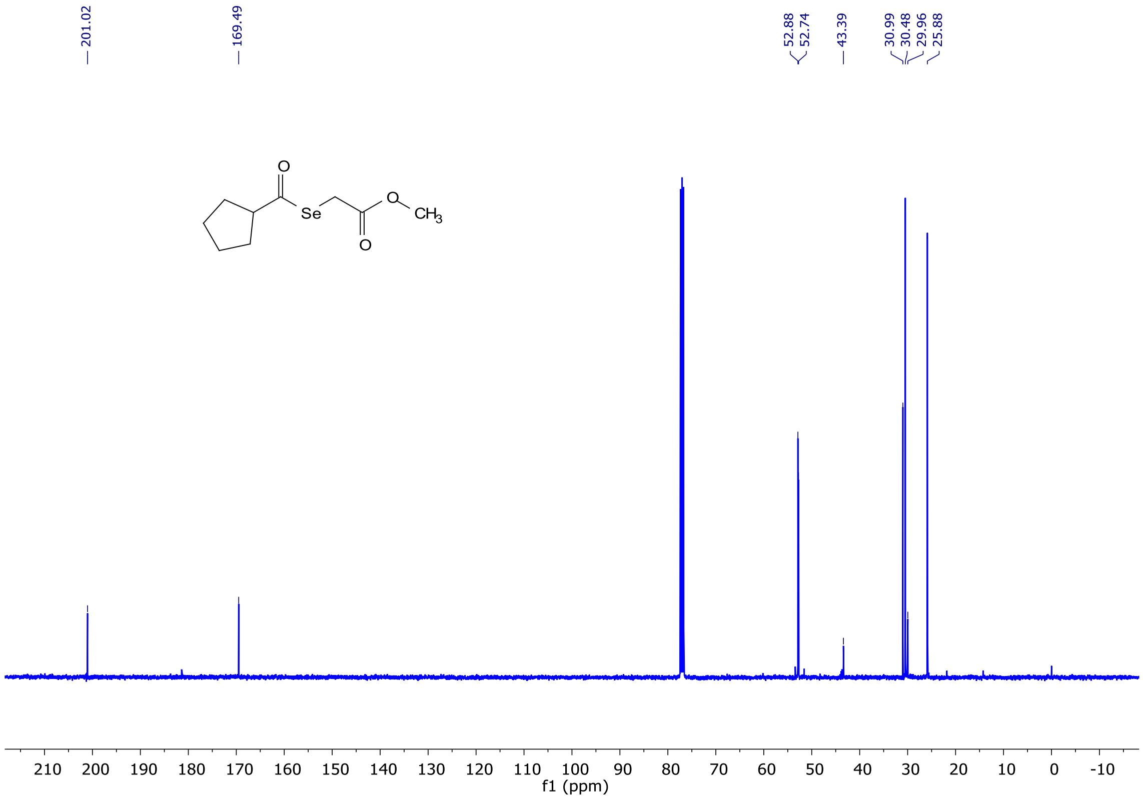

*Figure S60. ¹³C-NMR spectrum of compound **C7**.*

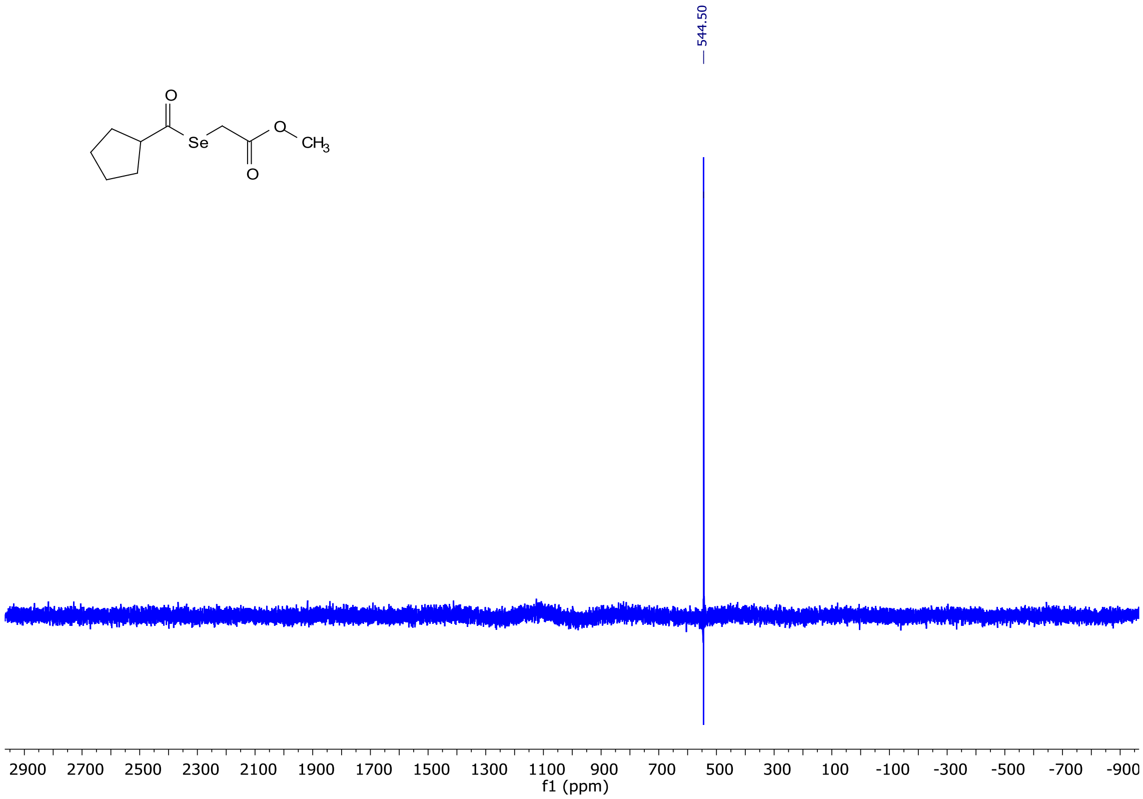

*Figure S61. ⁷⁷Se-NMR spectrum of compound **C7**.*

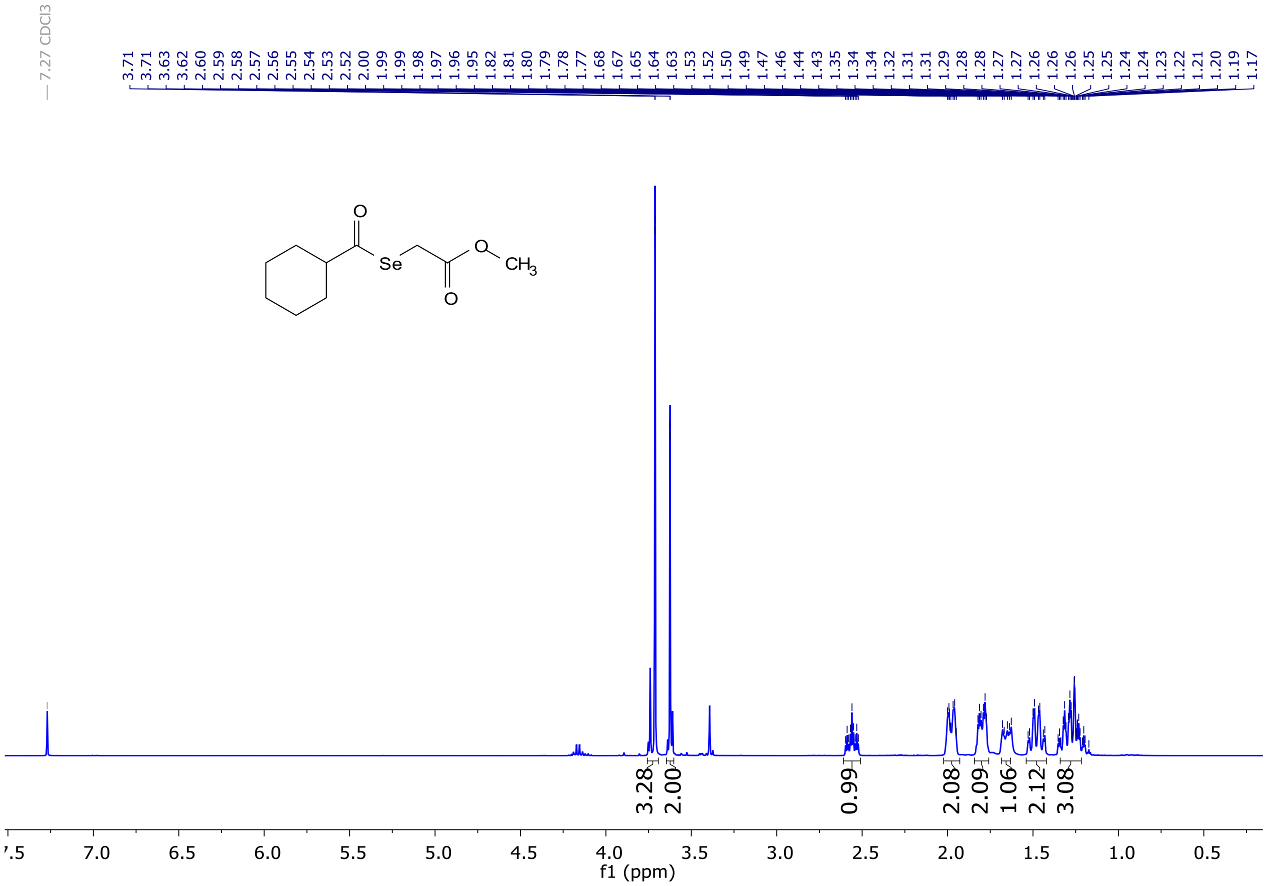

Figure S62. ^{1}H-NMR spectrum of compound ***C8.***

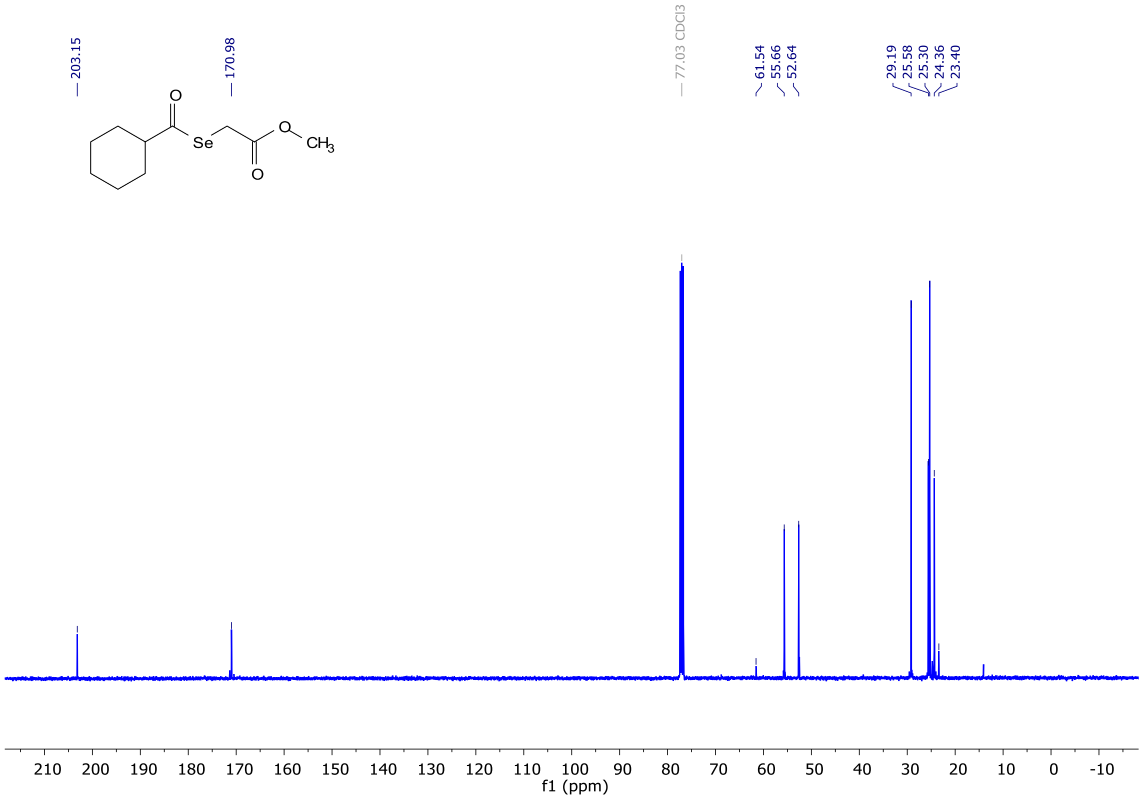

Figure S63. ^{13}C-NMR spectrum of compound ***C8***.

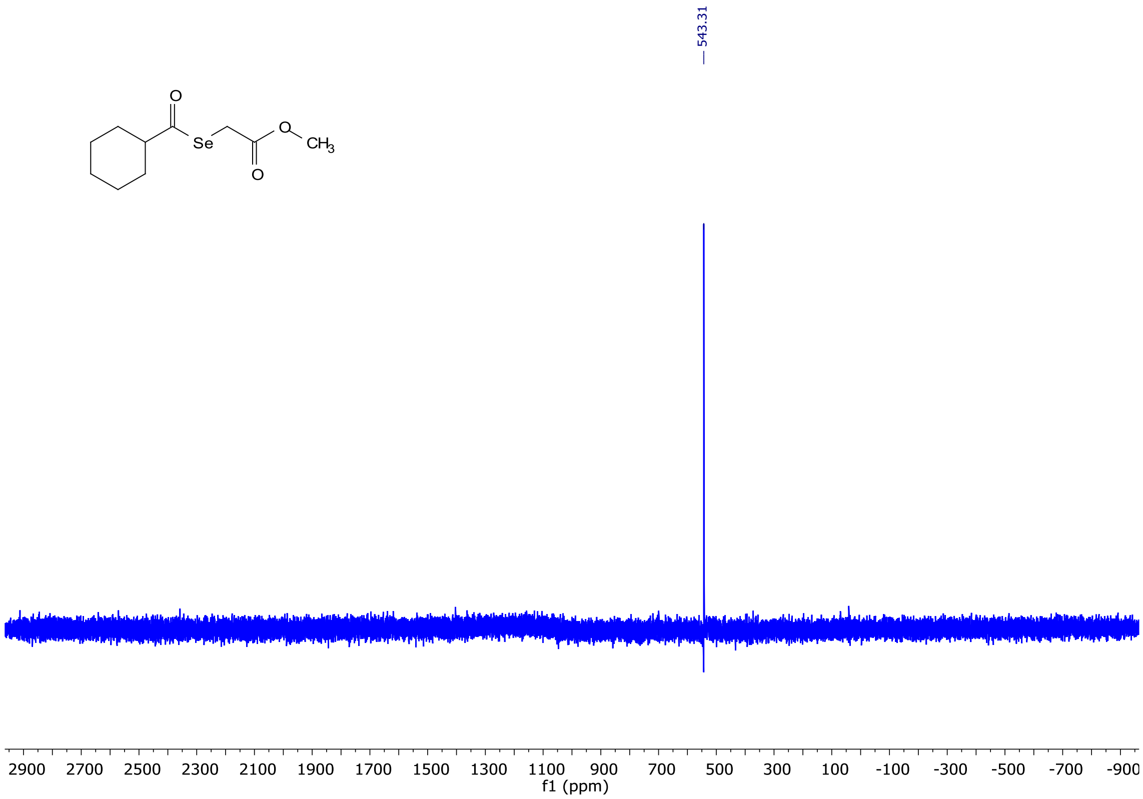

Figure S64. ^{77}Se-NMR spectrum of compound ***C8.***

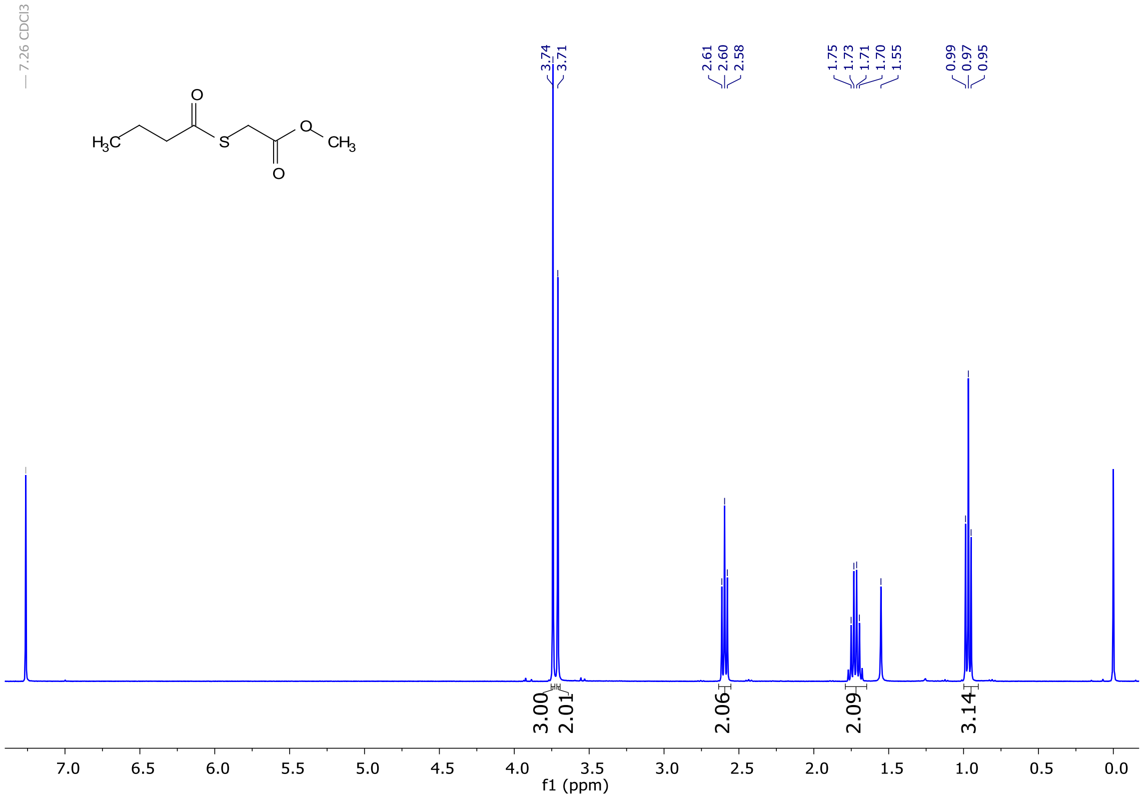

Figure S65. ^{1}H-NMR spectrum of compound ***D1.***

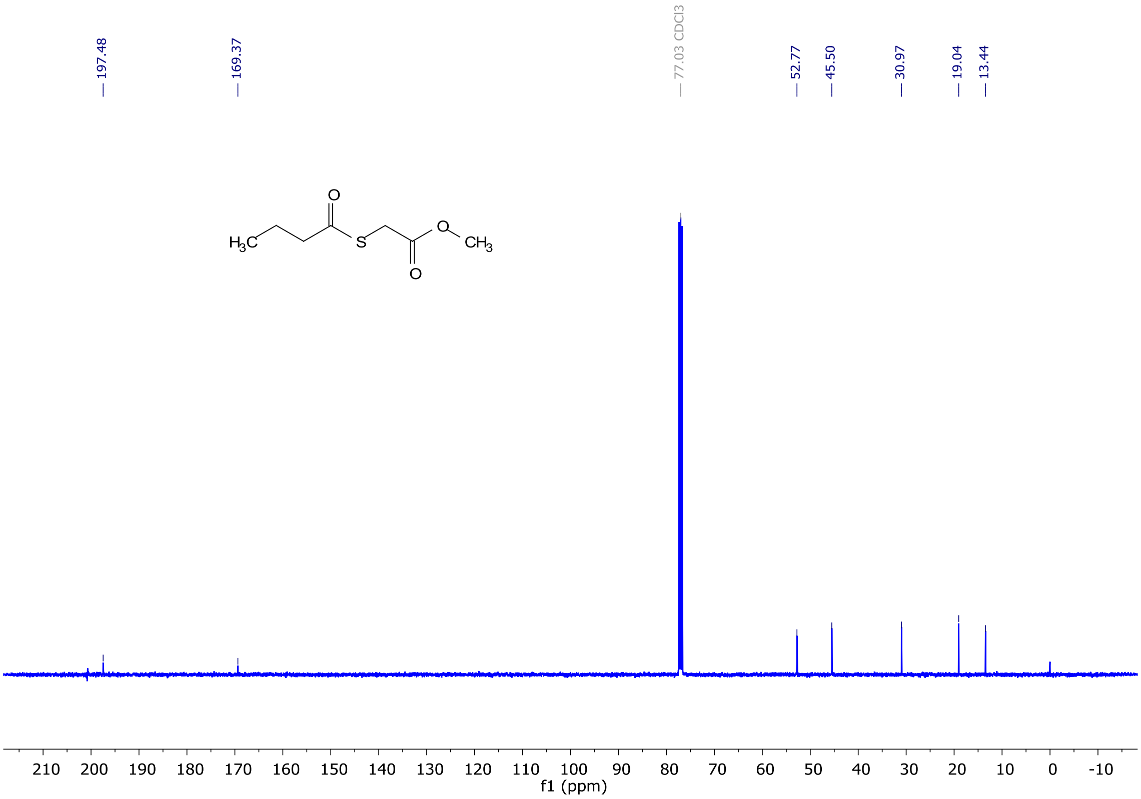

*Figure S66. ^{13}C-NMR spectrum of compound **D1**.*

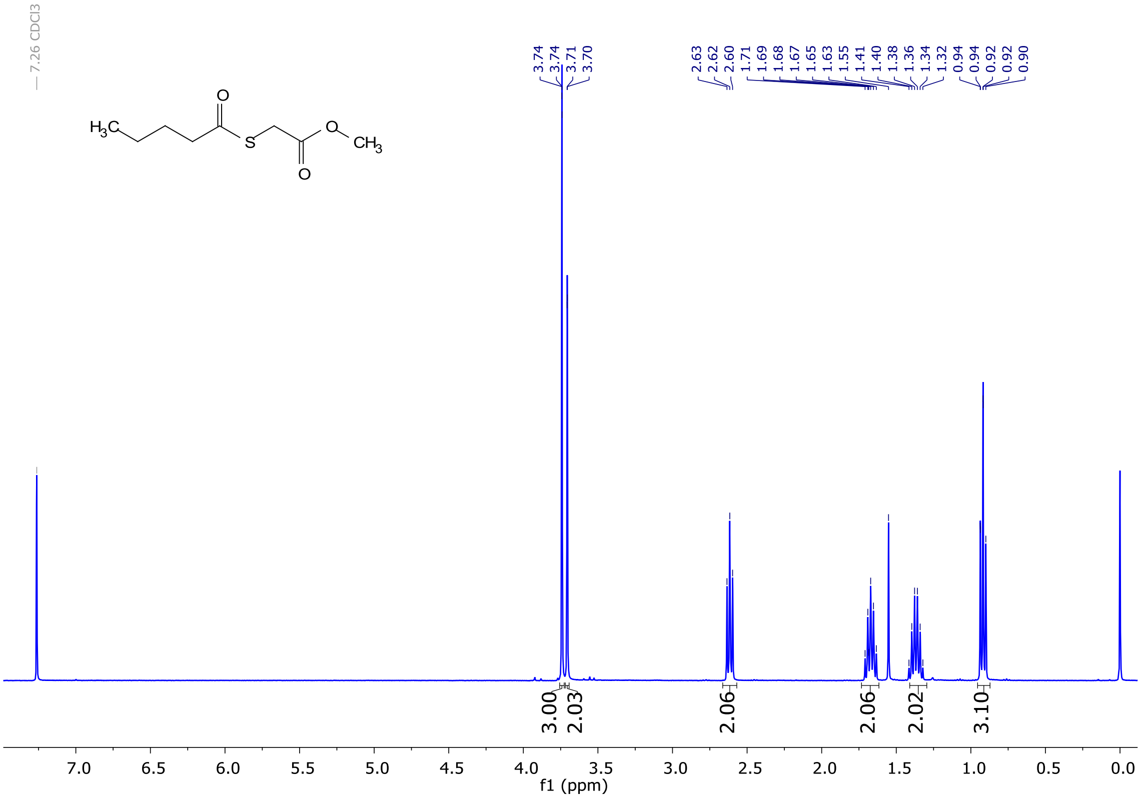

*Figure S67. ^{1}H-NMR spectrum of compound **D2.***

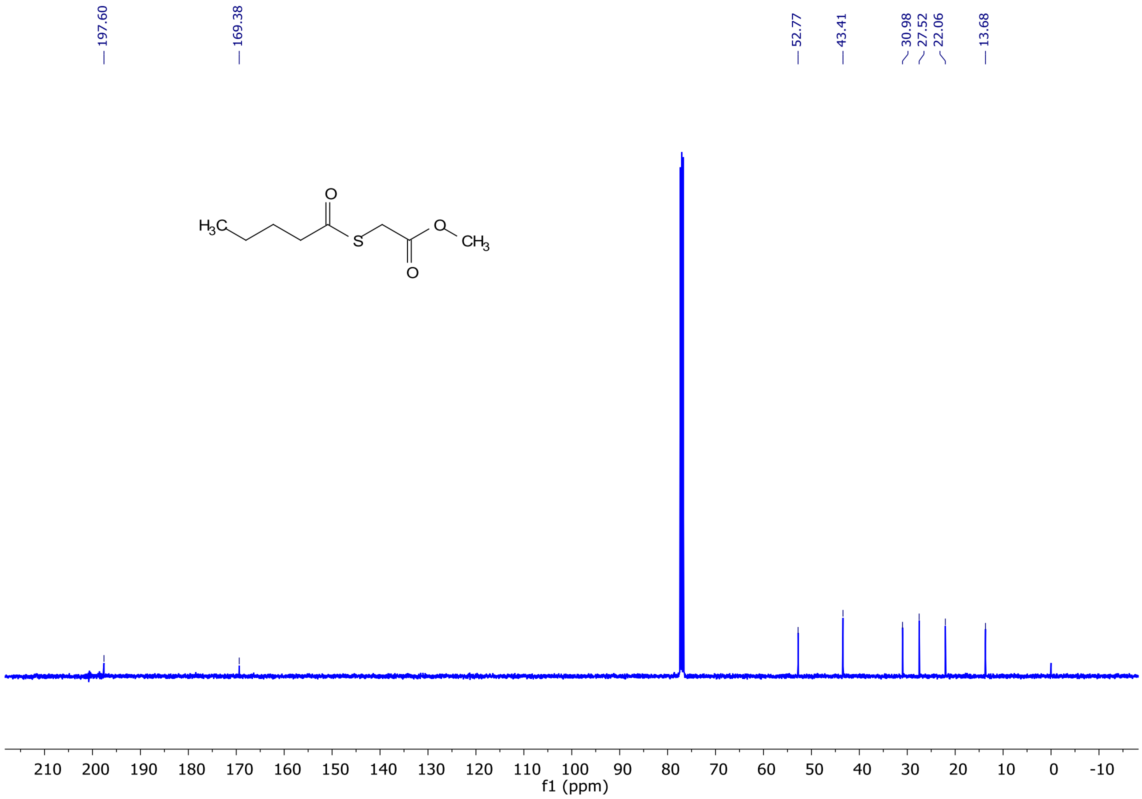

*Figure S68. ^{13}C-NMR spectrum of compound **D2**.*

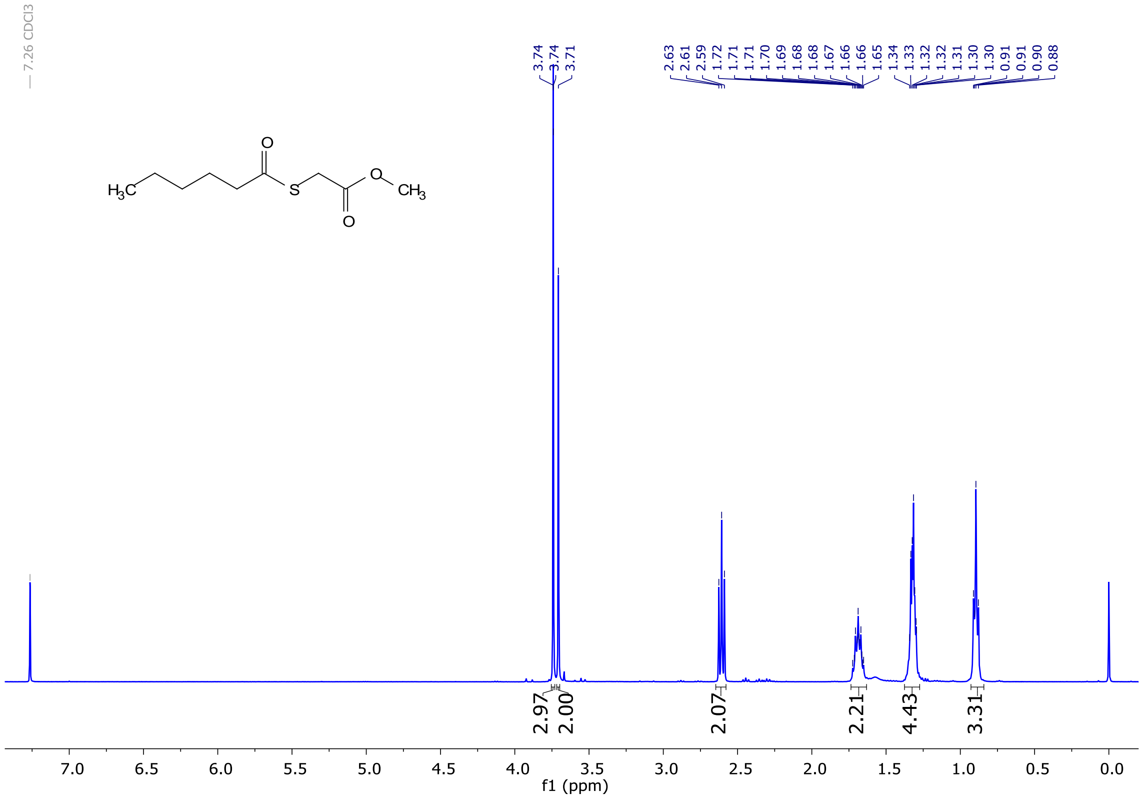

*Figure S69. ^{1}H-NMR spectrum of compound **D3.***

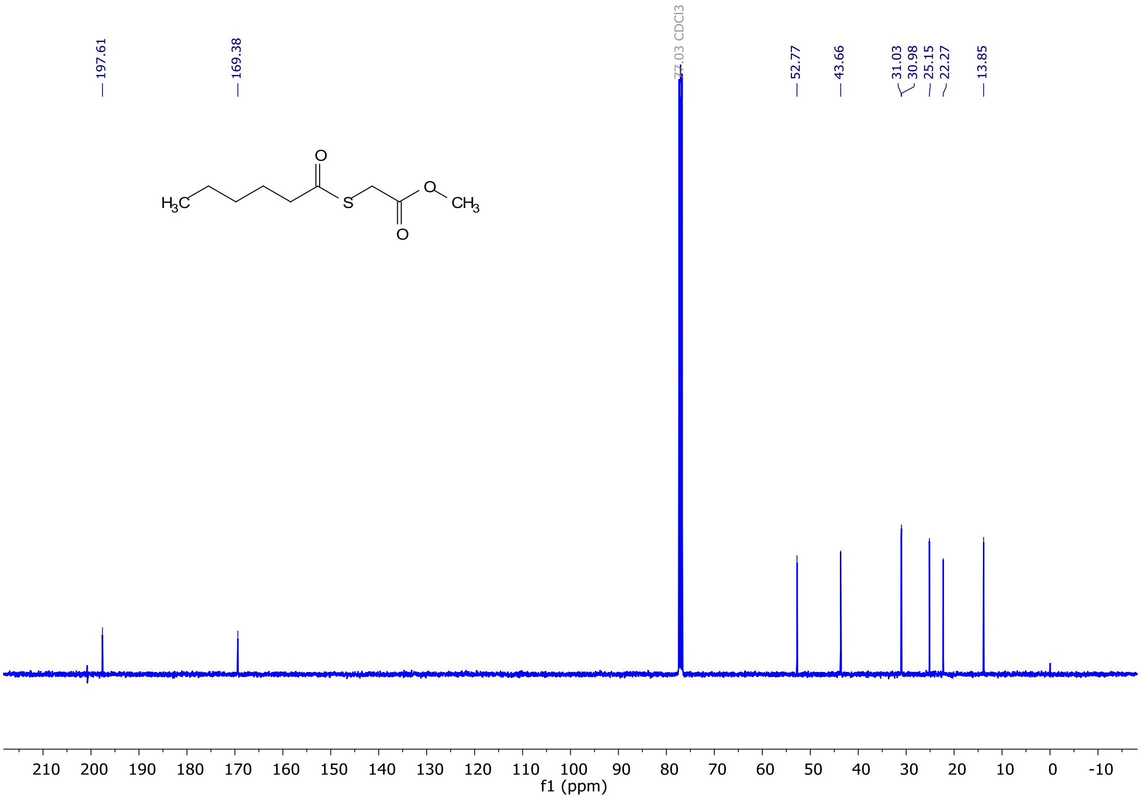

*Figure S70. ^{13}C-NMR spectrum of compound **D3**.*

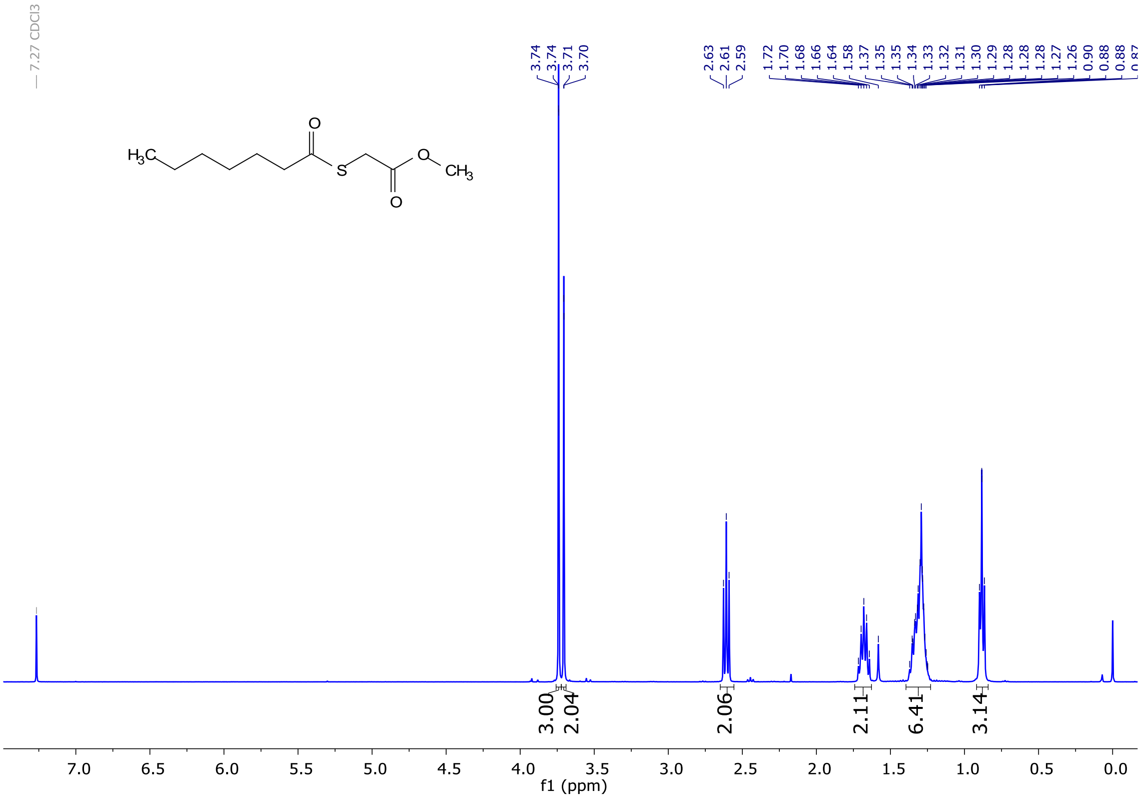

*Figure S71. ^{1}H-NMR spectrum of compound **D4.***

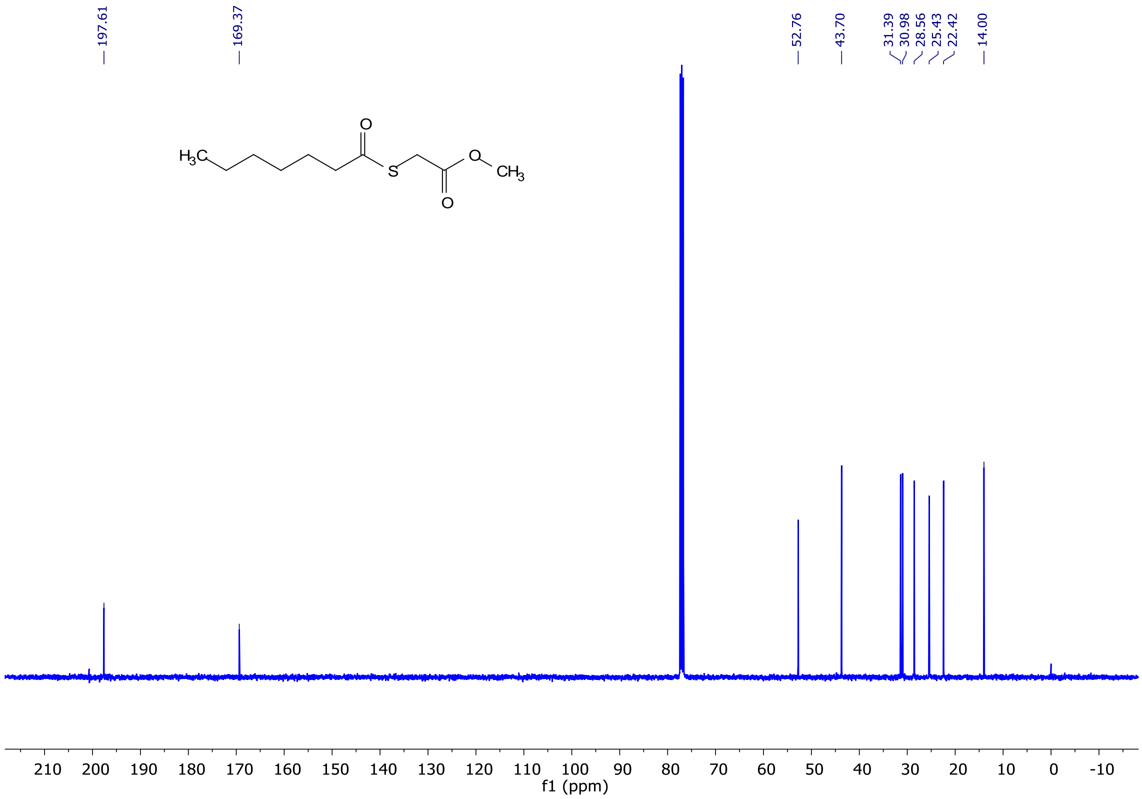

*Figure S72. ^{13}C-NMR spectrum of compound **D4**.*

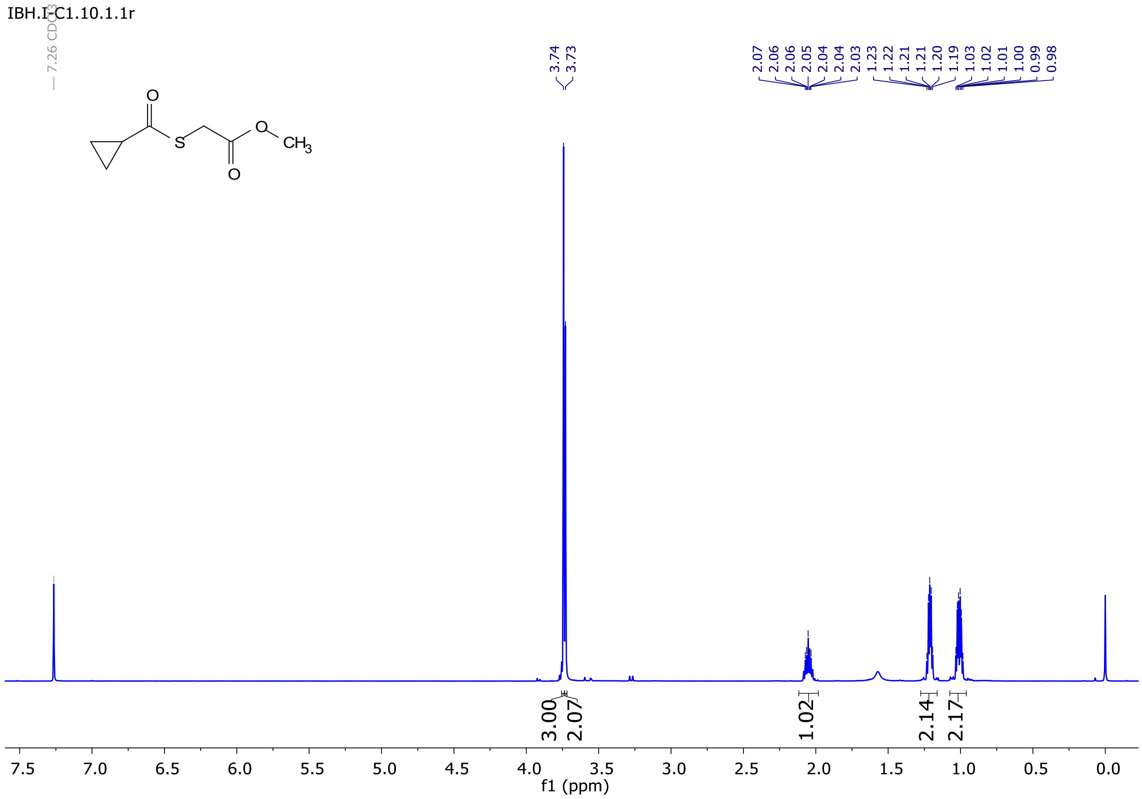

*Figure S73. ^{1}H-NMR spectrum of compound **D5.***

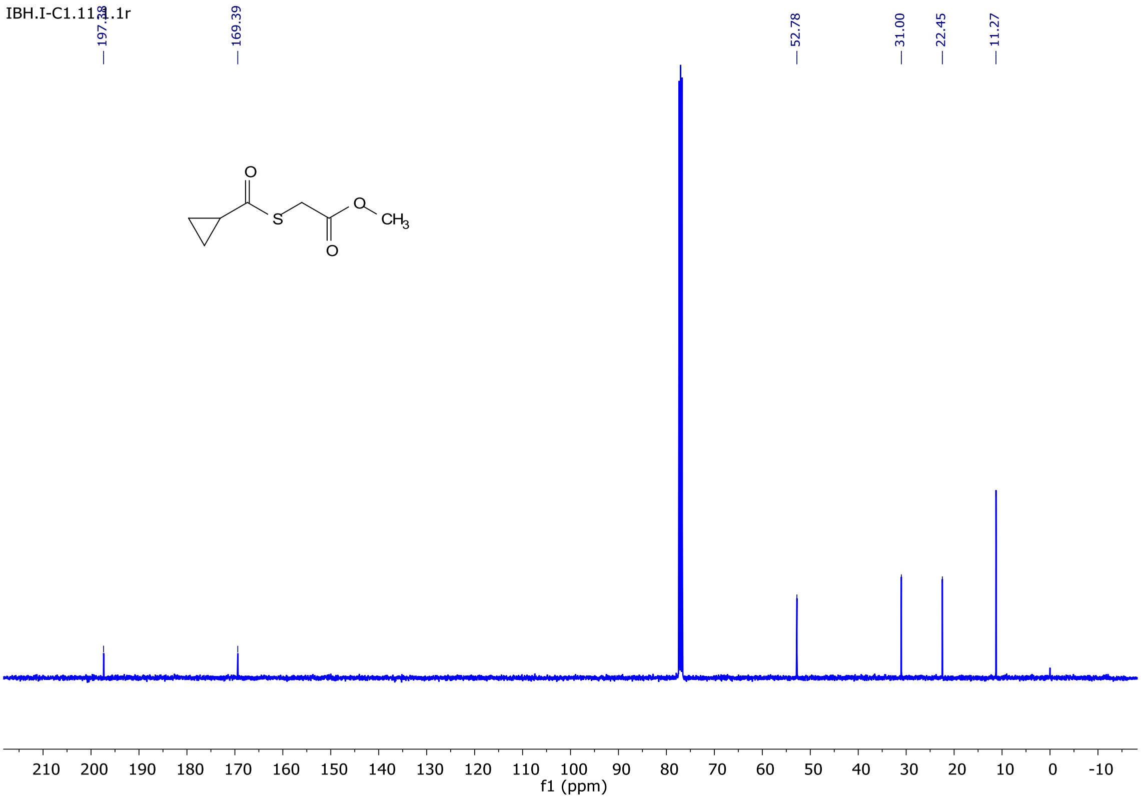

*Figure S74. ^{13}C-NMR spectrum of compound **D5**.*

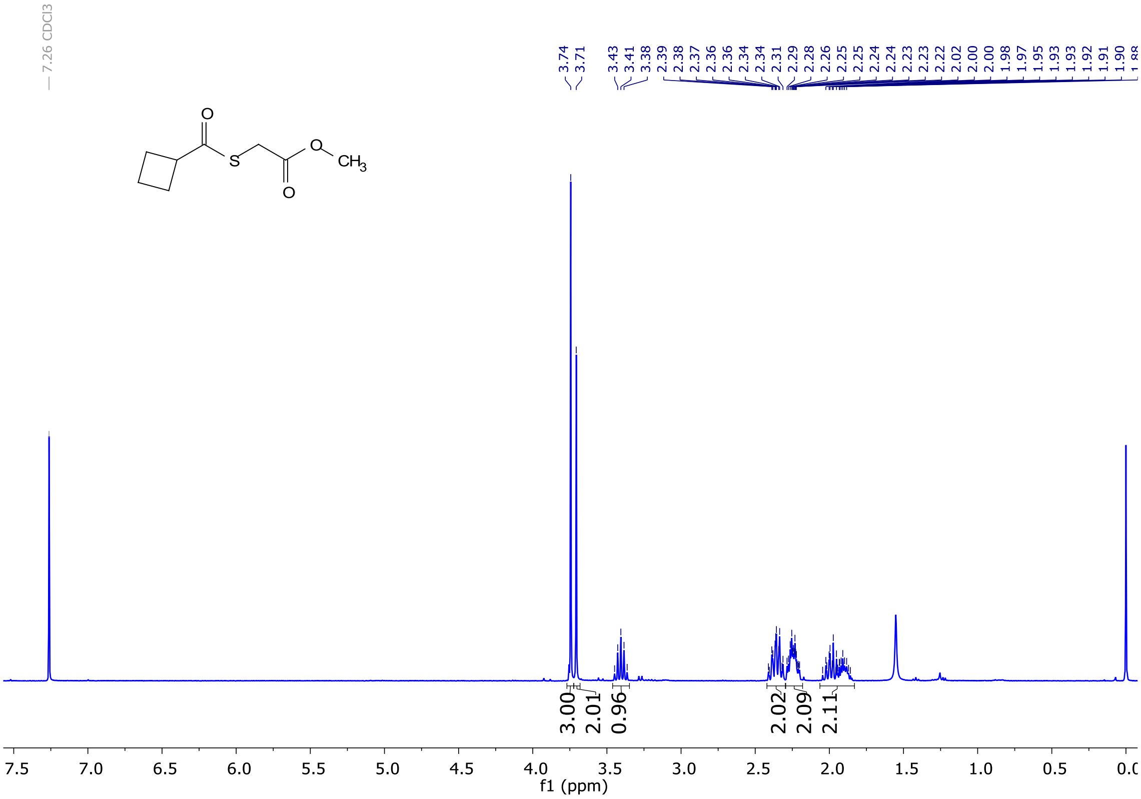

*Figure S75. ^{1}H-NMR spectrum of compound **D6.***

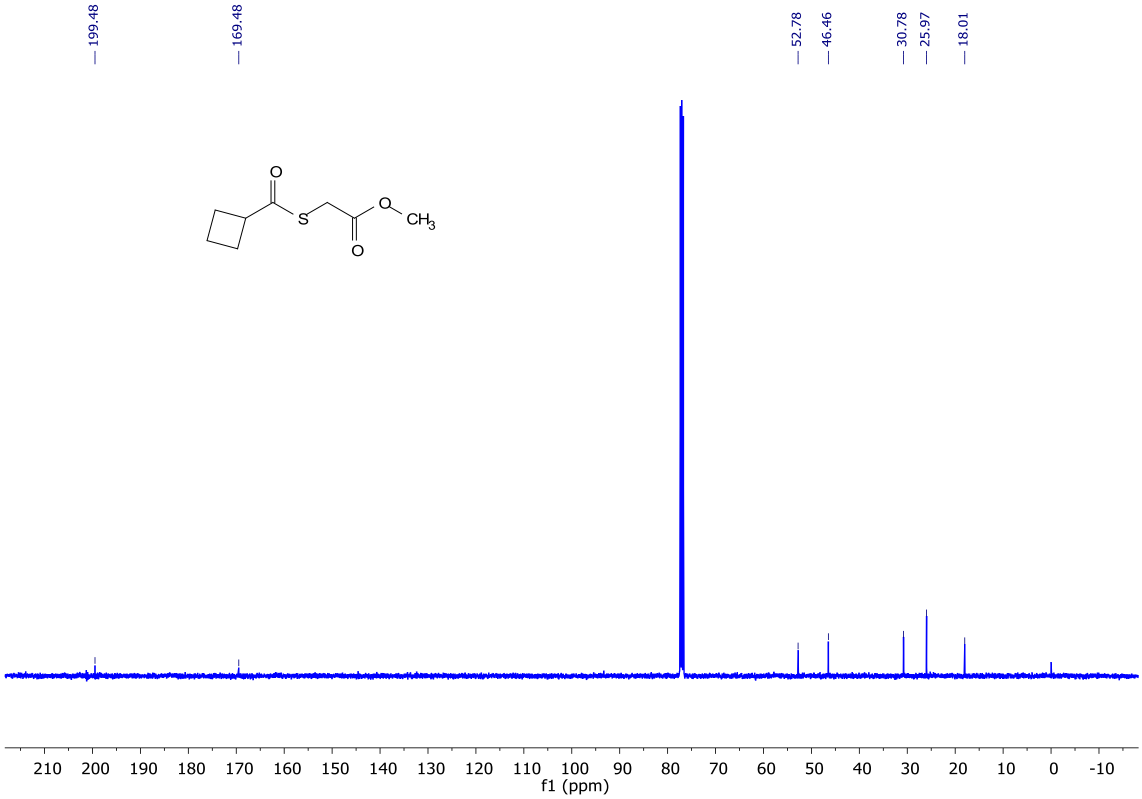

*Figure S76. ^{13}C-NMR spectrum of compound **D6**.*

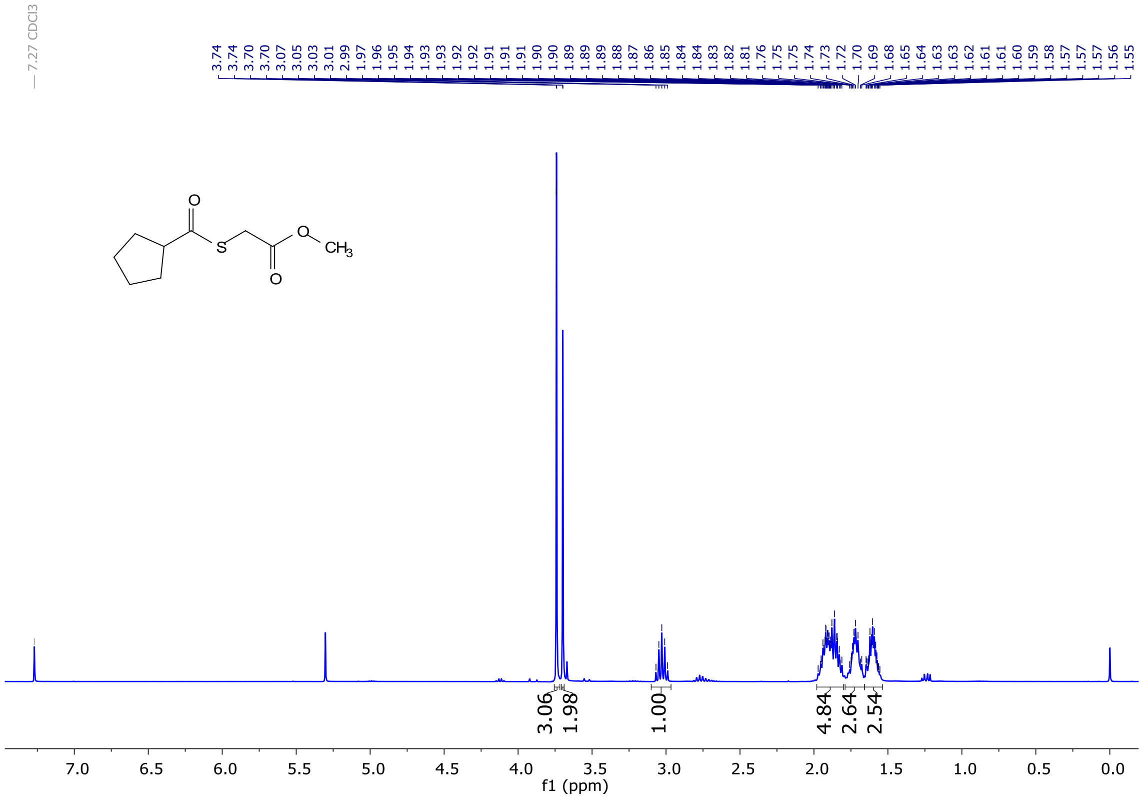

*Figure S77. ^{1}H-NMR spectrum of compound **D7**.*

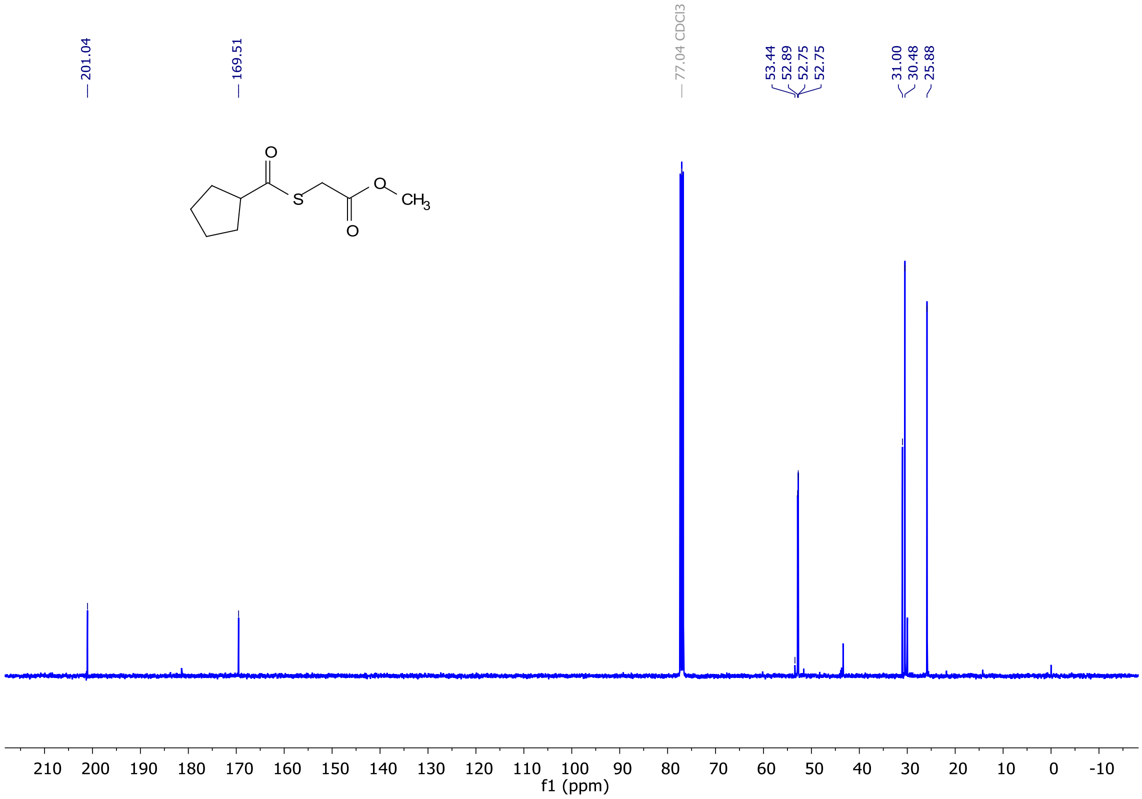

*Figure S78. ^{13}C-NMR spectrum of compound **D7**.*

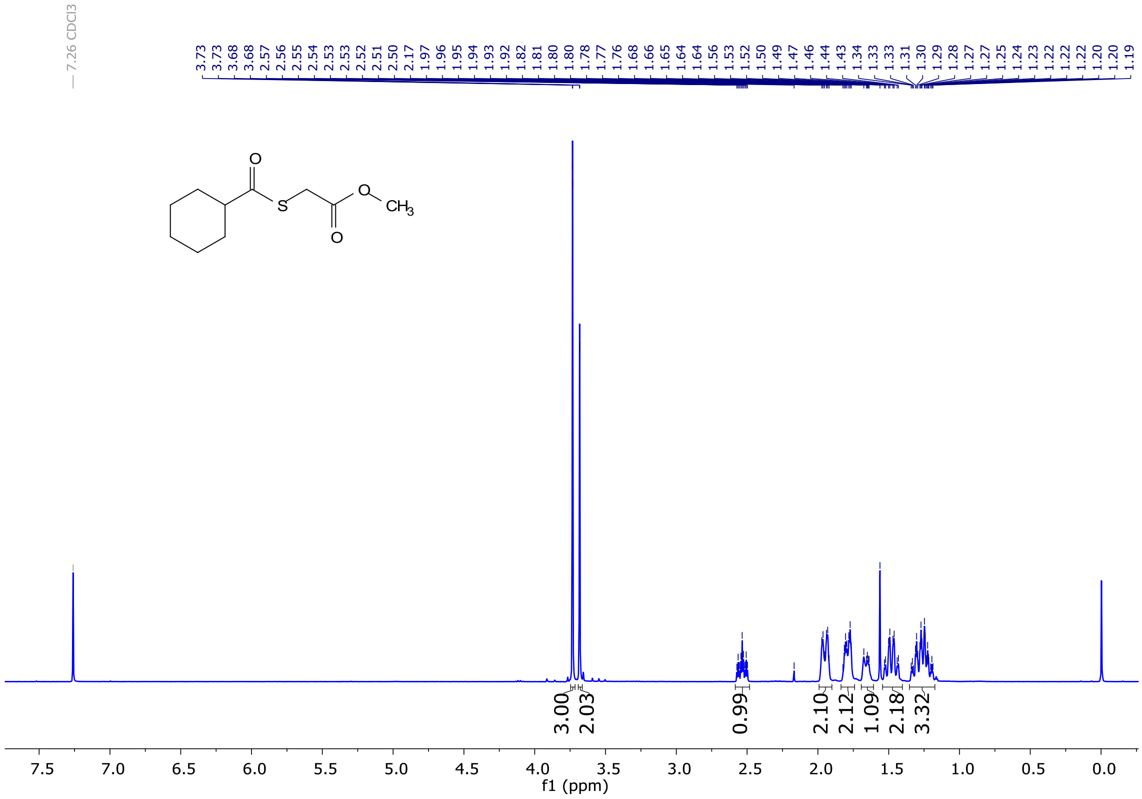

*Figure S79. ^{1}H-NMR spectrum of compound **D8.***

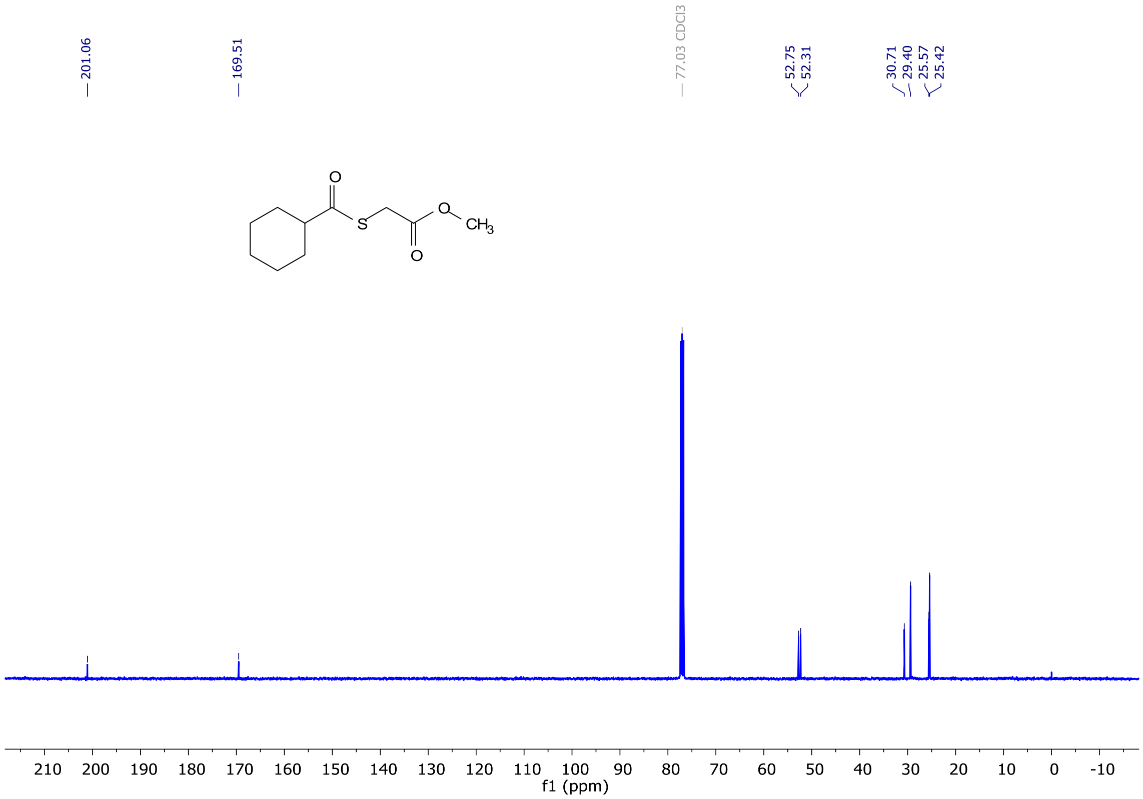

*Figure S80. ^{13}C-NMR spectrum of compound **D8**.*
